# An association between air pollution and daily most frequently visits of eighteen outpatient diseases in an industrial city

**DOI:** 10.1038/s41598-020-58721-0

**Published:** 2020-02-11

**Authors:** Tang-Tat Chau, Kuo-Ying Wang

**Affiliations:** 1 Department of Family Medicine, Landseed International Hospital, Tao-Yuan, Taiwan; 2 Department of Atmospheric Sciences, National Central University, Chung-Li, Taiwan

**Keywords:** Environmental impact, Epidemiology

## Abstract

Toxic effects of air pollutants were individually identified in various organs of the body. However, the concurrent occurrences and the connection of diseases in multiple organs arise from air pollution has not been concurrently studied before. Here we hypothesize that there exist connected health effects arise from air pollution when diseases in various organs were considered together. We used medical data from hospital outpatient visits for various organs in the body with a disease-air pollution model that represents each of the diseases as a function of the environmental factors. Our results show that elevated air pollution risks (above 40%) concurrently occurred in diseases of spondylosis, cerebrovascular, pneumonia, accidents, chronic obstructive pulmonary disease (COPD), influenza, osteoarthritis (OA), asthma, peptic ulcer disease (PUD), cancer, heart, hypertensive, diabetes, kidney, and rheumatism. Air pollutants that were associated with elevated health risks are particular matters with diameters equal or less than 2.5 *μ*m (*PM*_2.5_), nitrogen dioxide (*NO*_2_), ozone (*O*_3_), particular matters with diameters equal or less than 10 *μ*m (*PM*_10_), carbon monoxide (CO), and nitrogen oxide (NO). Concurrent occurrences of diseases in various organs indicate that the immune system tries to connectively defend the body from persistent and rising air pollution.

## Introduction

Air pollution is the most important environmental risk factor for health^1^. The most severe health risks associated with air pollution are the mortality rates, which have been studied the longest^2^. The 1952 London smog revealed an increase of 3000 mortality rates associated with continuous increases in sulfur dioxide (*SO*_2_) concentrations from 70 ppbv to 340 ppbv in 4 weeks^2^. Based on a sample of 8111 people for a period of 14 to 16 years (a total of 111,076 person-years), Dockery *et al*. found that *PM*_2.5_ contributes to excess mortality in six U.S. cities^3^. Hence an estimated latency period of mortality rates^4^ of weeks to years between causes (increased in air pollution levels) and effects (rising mortality rates in the population).

Before people got killed by diseases associated with air pollution, people were struck with various diseases associated with air pollution. The toxicity and pathophysiological changes in the organs resulting from air pollution exposure were best found in experimental studies^5^. Air pollutants induced 1.5 to 2 times heritable deoxyribonucleic acid (DNA) mutations in mice placed 1-km compared with mice placed 30-km downwind to two steel mills^6^. Deposited soots changed the physical outlook of bird specimens^7^. Elevated concentrations of selected persistent organic pollutants of polycyclic aromatic hydrocarbons (PAHs), o,p’-isomers of dichlorodiphenyltrichloroethane (DDT), metabolites, and *α*-hexachlorocyclohexane (HCH) were measured in the placenta and were associated with the increase in neural tube defects in pregnant women^8^. Elevated levels of insecticide p,p’-dichlorodiphenyldichloroethylene (p,p’-DDE) were measured in maternal serum specimens from early pregnancy and were associated with autism among offspring^9^. Elevated levels of DDT were measured in the maternal serum samples collected during the peak years (1959–1967) of DDT use in the United States compared with other samples of breast cancer collected in later decades, and found utero DDT exposure and risks of breast cancer in young women and a possible association with more aggressive tumors^10^.

Traffic-related air pollution has shown to trigger microglial activation and neuronal atrophy^11^, and affect embryonic and adult neurons through glutamatergic mechanisms in rodent models^12^. Structural brain magnetic resonance imaging scans revealed that older women with greater *PM*_2.5_ exposures had significantly smaller white matter present in frontal and temporal lobes and corpus callosum^5^. Combustion-derived iron-rich magnetite nanoparticles from airborne particulate matter pollution were found in the human brain^13^, showing that air pollution particles were directly transported and deposited in the human brain. Mice exposed to elevated levels of *NO*_2_ (2.5–5.0 mg/*m*^3^) were found to show the deterioration of spatial learning and memory, aggravated amyloid *β*_42_ accumulation, promoted pathological abnormalities, and cognitive defects related to Alzheimer’s disease^14^. Humans exposed to *NO*_2_ and *PM*_2.5_ were positively correlated with the incidence of dementia in London, England^15^. Toxins accompanying air pollutants into the body exert chronic activation of microglia, leading to direct neuronal damage and neuronal death in the central nervous system^16^ Delicate communication exists between the brain and the immune system^17,18^, and evidence that connects health and emotions through neural activity in the brain^19^. These pathophysiological findings reveal that air pollutants affected the central nervous system, leading to the decline of brain function^20^ and fatal diseases^18^.

Pathophysiological evidence convincingly showed pathways of various diseases arose from air pollution^21^. Hence, more diseases associated with air pollution remained to be discovered in the population^22^. Kampa and Castanas^23^ and Jay^24^ showed that diseases associated with air pollution were found in various parts of human organs: cardiovascular and circulatory system, digestive and excretory system, endocrine system, integumentary and exocrine system, lymphatic and immune system, muscular and skeletal system, nervous system, renal and urinary system, reproductive system, respiratory system, and sensory system. Darnall^25^ hypothesized a whole body approach to study pains, which are often associated with diseases. FitzGerald *et al*.^26^ proposed that humans are model organisms for future medicine for treating diseases.

Though air pollution concentrations have gradually declined in the developed country, air pollution levels have continuously increased in the developing countries^27^. Population in the industrial cities are particularly at risks of air pollution^2,3,28^. In this work, we hypothesize that hospital outpatient visits for diseases contain health signals from the entire body organs that are related to air pollution. Diseases associated with air pollution in various organs were individually studied before, but the concurrence of diseases from various organs that were associated with air pollution hasn’t been studied in an integrated and connected approach and perspective. To prove this hypothesis, in this work we studied 18 diseases that were recorded during the hospital outpatient visits. These 18 diseases represent the mostly visited diseases for the outpatients to the Taiwan Landseed Hospital during the period 2007–2011. Wang and Chau^29^ used daily outpatient visits to find an association between air pollution and daily outpatients of respiratory diseases. Chau and Wang^30^ found an association between temperatures and daily outpatients for accidents. Note that increased in temperature was found to deteriorate mental health^31^. In this work, we scaled up the analysis by Wang and Chau^29^ and Chau and Wang^30^ to investigate the association of daily hospital visits of 18 diseases with air pollution. These 18 diseases arise from various body organs that were impacted by air pollution^23,24^. A total of 1.7 million medical visits from 18 diseases during 2007–2011 were combined with half a million air pollution and meteorological data for big data analysis. We show that measurable data of medical visits and environmental measurements reveal diseases that arise from various organs are concurrently and connectively linked to air pollution.

## Data and Methods

### A disease-air pollution model

A multivariate disease-air pollution model used in this work were used in previous studies in finding an association between hospital outpatient visits for respiratory diseases and environmental factors^29^, and between hospital outpatient visits for accidents and environmental factors^30^. In this section, we describe detailed computational steps used in this work with the same multivariate model.

The time series of daily (*t*) medical records ${M}_{i,j}(t)$ from each disease *i* of the 18 diseases for each year *j* of 2007–2011 and each group of 3-age groups of outpatients (0–15, 16–65, and >65 years old) were calculated with respect to 12 time series of daily environmental parameters (7 air pollution parameters of *PM*_10_, *PM*_2.5_, *O*_3_, *CO*, *NO*_2_, *NO*, *SO*_2_; and 5 meteorological parameters of temperature, rainfall, wind direction, wind speed, and relative humidity):

1 $${M}_{i,j}(t)=(\begin{array}{l}{\beta }_{i,j,0}+\\ {\beta }_{i,j,1}{[P{M}_{10}(t)]}_{j}+\\ {\beta }_{i,j,2}{[P{M}_{2.5}(t)]}_{j}+\\ {\beta }_{i,j,3}{[{O}_{3}]}_{j}(t)+\\ {\beta }_{i,j,4}{[CO]}_{j}(t)+\\ {\beta }_{i,j,5}{[NO]}_{j}(t)+\\ {\beta }_{i,j,6}{[N{O}_{2}]}_{j}(t)+\\ {\beta }_{i,j,7}{[S{O}_{2}]}_{j}(t)+\\ {\beta }_{i,j,8}{[T]}_{j}(t)+\\ {\beta }_{i,j,9}{[P]}_{j}(t)+\\ {\beta }_{i,j,10}{[WD]}_{j}(t)+\\ {\beta }_{i,j,11}{[WS]}_{j}(t)+\\ {\beta }_{i,j,12}{[RH]}_{j}(t)\end{array})={\beta }_{i,j,0}+{x}_{j}{B}_{i,j}$$

where

2 $${B}_{i,j}=(\begin{array}{c}{\beta }_{i,j,1}\\ {\beta }_{i,j,2}\\ {\beta }_{i,j,3}\\ {\beta }_{i,j,4}\\ {\beta }_{i,j,5}\\ {\beta }_{i,j,6}\\ {\beta }_{i,j,7}\\ {\beta }_{i,j,8}\\ {\beta }_{i,j,9}\\ {\beta }_{i,j,10}\\ {\beta }_{i,j,11}\\ {\beta }_{i,j,12}\end{array}).$$

The transposed of the parameter array *x*_*i*_ is

3 $${({x}_{j})}^{t}=(\begin{array}{c}{[P{M}_{10}]}_{j}\\ {[P{M}_{2.5}]}_{j}\\ {[{O}_{3}]}_{j}\\ {[CO]}_{j}\\ {[NO]}_{j}\\ {[N{O}_{2}]}_{j}\\ {[T]}_{j}\\ {[P]}_{j}\\ {[WD]}_{j}\\ {[WS]}_{j}\\ {[RH]}_{j}\end{array}).$$

Here *i* represents each of the 18 diseases, *i* = 1, …, 18; *j* represents each year of 2007, …, 2011; *M* is the outpatient numbers; T is temperature; P is daily accumulation precipitation; WD is wind direction; WS is wind speed, and RH is relative humidity. In the application of Eq. 1, medical data ${M}_{i,j}$ and parameter arrays ${x}_{i}$ are known from real-world data. Hence, we solve Eq. 1 for finding the association coefficient matrix ${B}_{i,j}$.

Notice that time-series data of daily outpatient number ${M}_{i,j}$ of a disease, and 12 environmental factors are known from input data. The holidays and weekends were removed from the time-series data to avoid the artificial effect of low outpatients on these public holidays^29,30^. Except for the removal of medical and environmental data from public holidays, no other processes were applied to the input time-series data. All environmental data were tested for normal distribution using the Chi-square test^32^. Except for rainfall and wind directions, the environmental data resembles the patterns of normal distribution^29^. Test of the normal distribution of medical data also exhibit patterns of normal distribution^29,30^ (shown also in Supplementary Materials). All environmental data that were used as model variables were normalized by the maximum values of each variable to ensure that the computation were on the same unitless ground when making the comparison^33^.

**Figure 1:**
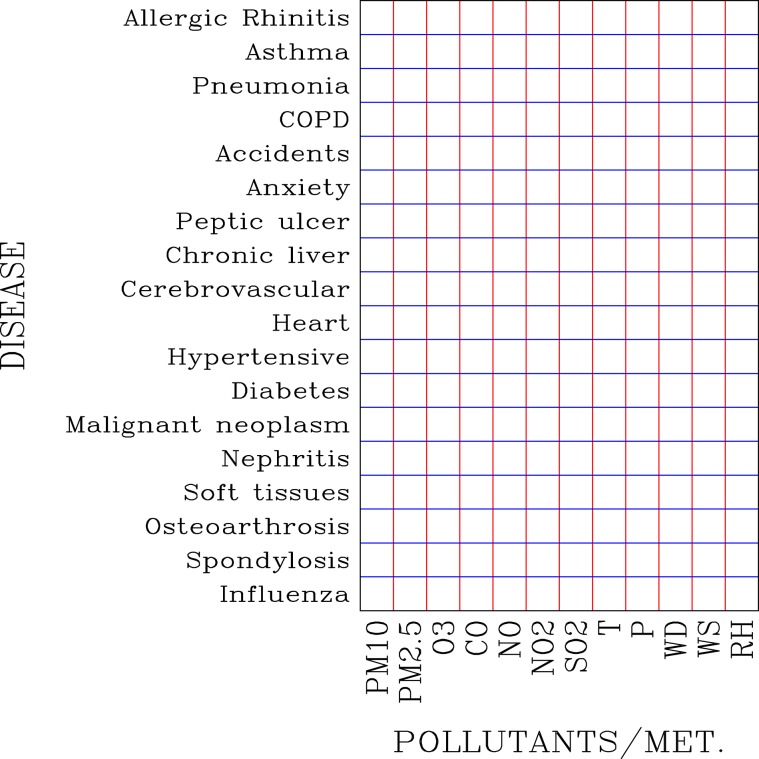
A schematic diagram showing the distribution of correlation coefficient matrix of diseases (y-axis) with respect to parameters of air pollutants and meteorological factors (x-axis). Each empty box represents a correlation coefficient to be determined from the disease-air pollution model shown in Eq. (1).

The environmental factors are $P{M}_{10}$, $P{M}_{2.5}$, *O*_3_, CO, NO, $N{O}_{2}$, $S{O}_{2}$, T, P, WD, WS, and RH. The daily outpatient visits were computed according to the 3 age groups of outpatients (0–15, 16–65, and higher than 65 years old as in Wang and Chau^29^ and Chau and Wang^30^. A spectrum of 18 diseases was sorted based on the International Classification of Diseases, 9th Revision (ICD-9) as shown in Table 1. These 18 diseases include the diseases of the respiratory system, such as allergic rhinitis (AR), asthma, pneumonia, and COPD; accidents; mental disorders (anxiety, dissociative, somatoform disorders); the digestive system, such as PUD, and chronic liver diseases and cirrhosis; circulatory system, such as cerebrovascular disease, heart disease, and hypertensive disease; diabetes mellitus; malignant neoplasm (cancer); genitourinary system (nephritis, nephritis, and nephrosis); musculoskeletal system, such as other disorders of soft tissues, OA and allied disorders, and spondylosis and allied disorders; and influenza. Figure 1 shows a schematic diagram of distribution of association coefficients *β*_*i*,*j*_ with respect to diseases and environmental factors.

**Table 1:** List The International Classification of Diseases, 9th Revision (ICD-9), For This Wok.

Disease	ICD-9 Codes
**Diseases of the respiratory system**	
Allergic Rhinitis	477
Asthma	493
Pneumonia	480–486
Chronic obstructive pulmonary disease	490–493
Accidents	800–949
**Mental disorders**	
Anxiety, dissociative and somatoform disorders	300
**Diseases of the digestive system**	
Peptic ulcer	533
Chronic liver disease and cirrhosis	571
**Diseases of the circulatory system**	
Cerebrovascular disease	430–438
Heart disease	390–392, 393–398, 410–414, 420–429
Hypertensive disease	401–405
**Endocrine disorders**	
Diabetes mellitus	250
Malignant neoplasm	140–208
**Diseases of the genitourinary system**	
Nephritis, nephrotic syndrome, and nephrosis	580–589
**Diseases of the musculoskeletal system and connective tissue**	
Other disorders of soft tissues	729
Osteoarthrosis and allied disorders	715
Spondylosis and allied disorders	721
Influenza vaccine injection

As the time-series patterns of outpatient visits to the hospital were shown to lag the time-series patterns of environmental factors, the association coefficients in Eq. 1 were solved for eight time-lag scenarios. These 8 scenarios are the hospital outpatient visits for diseases lag an environmental factor at 0-day, 1-day, 2-day, and so on, to 7-day.

### Numerical solutions of the disease-air pollution model

In order to compute disease air pollution risks and air pollution risks, we need to compute association coefficients shown in Eq. 1. For a disease *i*, we take partial directives of Eq. 1 with repect to each of the 12 environmental variables. Hence,

4 $$\frac{\partial {M}_{i,j}}{\partial {[P{M}_{10}]}_{j}}=(\begin{array}{l}{\beta }_{i,j,1}+\\ {\beta }_{i,j,2}\frac{\partial {[P{M}_{2.5}]}_{j}}{\partial {[P{M}_{10}]}_{j}}+\\ {\beta }_{i,j,3}\frac{\partial {[{O}_{3}]}_{j}}{\partial {[P{M}_{10}]}_{j}}+\\ {\beta }_{i,j,4}\frac{\partial {[CO]}_{j}}{\partial {[P{M}_{10}]}_{j}}+\\ {\beta }_{i,j,5}\frac{\partial {[NO]}_{j}}{\partial {[P{M}_{10}]}_{j}}+\\ {\beta }_{i,j,6}\frac{\partial {[N{O}_{2}]}_{j}}{\partial {[P{M}_{10}]}_{j}}+\\ {\beta }_{i,j,7}\frac{\partial {[S{O}_{2}]}_{j}}{\partial {[P{M}_{10}]}_{j}}+\\ {\beta }_{i,j,8}\frac{\partial {[T]}_{j}}{\partial {[P{M}_{10}]}_{j}}+\\ {\beta }_{i,j,9}\frac{\partial {[P]}_{j}}{\partial {[P{M}_{10}]}_{j}}+\\ {\beta }_{i,j,10}\frac{\partial {[WD]}_{j}}{\partial {[P{M}_{10}]}_{j}}+\\ {\beta }_{i,j,11}\frac{\partial {[WS]}_{j}}{\partial {[P{M}_{10}]}_{j}}+\\ {\beta }_{i,j,12}\frac{\partial {[RH]}_{j}}{\partial {[P{M}_{10}]}_{j}}\end{array})$$

and

5 $$\frac{\partial {M}_{i,j}}{\partial {[PM2.5]}_{j}}=(\begin{array}{l}{\beta }_{i,j,1}\frac{\partial {[P{M}_{10}]}_{j}}{\partial {[P{M}_{2.5}]}_{j}}+\\ {\beta }_{i,j,2}+\\ {\beta }_{i,j,3}\frac{\partial {[{O}_{3}]}_{j}}{\partial {[P{M}_{2.5}]}_{j}}+\\ {\beta }_{i,j,4}\frac{\partial {[CO]}_{j}}{\partial {[P{M}_{2.5}]}_{j}}+\\ {\beta }_{i,j,5}\frac{\partial {[NO]}_{j}}{\partial {[P{M}_{2.5}]}_{j}}+\\ {\beta }_{i,j,6}\frac{\partial {[N{O}_{2}]}_{j}}{\partial {[P{M}_{2.5}]}_{j}}+\\ {\beta }_{i,j,7}\frac{\partial {[S{O}_{2}]}_{j}}{\partial {[P{M}_{2.5}]}_{j}}+\\ {\beta }_{i,j,8}\frac{\partial {[T]}_{j}}{\partial {[P{M}_{2.5}]}_{j}}+\\ {\beta }_{i,j,9}\frac{\partial {[P]}_{j}}{\partial {[P{M}_{2.5}]}_{j}}+\\ {\beta }_{i,j,10}\frac{\partial {[WD]}_{j}}{\partial {[P{M}_{2.5}]}_{j}}+\\ {\beta }_{i,j,11}\frac{\partial {[WS]}_{j}}{\partial {[P{M}_{2.5}]}_{j}}+\\ {\beta }_{i,j,12}\frac{\partial {[RH]}_{j}}{\partial {[P{M}_{2.5}]}_{j}}\end{array})$$

Here $\partial {M}_{i,j}$/$\partial {[P{M}_{10}]}_{j}$ means variations of outpatients ${M}_{i,j}$ for disease *i* with respect to the variations of ${[P{M}_{10}]}_{j}$ in year *j*; and $\partial {M}_{i,j}$/$\partial {[P{M}_{2.5}]}_{j}$ means variations of outpatients ${M}_{i,j}$ for disease *i* with respect to the variations of ${[P{M}_{2.5}]}_{j}$ in year *j*. For the terms on the right-hand side of Eg. 4, $\partial {[P{M}_{2.5}]}_{j}$/$\partial {[P{M}_{10}]}_{j}$ means the variations of ${[P{M}_{2.5}]}_{j}$ associated with the changes in ${[P{M}_{10}]}_{j}$, and so on for the rest of other terms. These terms represent changes with respect to ${[P{M}_{10}]}_{j}$, indicating the coupling effects between environmental factors.

Similarly, we take partial directives of ${M}_{i,j}$ for a disease *i* with respect to the rest of the other environmental variables. Hence, we write this system of 12 equations with respect to a disease *i* in *j* year as

6 $${A}_{i,j}={J}_{i,j}{B}_{i,j}.$$

where *A*_*i*,*j*_ is

7 $${A}_{i,j}=(\begin{array}{c}\frac{\partial {M}_{i,j}}{\partial {[P{M}_{10}]}_{j}}\\ \frac{\partial {M}_{i,j}}{\partial {[P{M}_{2.5}]}_{j}}\\ \frac{\partial {M}_{i,j}}{\partial {[{O}_{3}]}_{j}}\\ \frac{\partial {M}_{i,j}}{\partial {[CO]}_{j}}\\ \frac{\partial {M}_{i,j}}{\partial {[NO]}_{j}}\\ \frac{\partial {M}_{i,j}}{\partial {[N{O}_{2}]}_{j}}\\ \frac{\partial {M}_{i,j}}{\partial {[S{O}_{2}]}_{j}}\\ \frac{\partial {M}_{i,j}}{\partial {[T]}_{j}}\\ \frac{\partial {M}_{i,j}}{\partial {[P]}_{j}}\\ \frac{\partial {M}_{i,j}}{\partial {[WD]}_{j}}\\ \frac{\partial {M}_{i,j}}{\partial {[WS]}_{j}}\\ \frac{\partial {M}_{i,j}}{\partial {[RH]}_{j}}\end{array}).$$

The Jacobian *J*_*i*,*j*_ of this disease-air pollution model for a disease *i* is written as

8 $${J}_{i,j}=(\begin{array}{cccccc}1 & \frac{\partial {f}_{i,j,2}}{\partial {x}_{i,j,1}} & \frac{\partial {f}_{i,j,3}}{\partial {x}_{i,j,1}} & \cdot \cdot \cdot  & \frac{\partial {f}_{i,j,11}}{\partial {x}_{i,j,1}} & \frac{\partial {f}_{i,j,12}}{\partial {x}_{i,j,1}}\\ \frac{\partial {f}_{i,j,1}}{\partial {x}_{i,j,2}} & 1 & \frac{\partial {f}_{i,j,3}}{\partial {x}_{i,j,2}} & \cdot \cdot \cdot  & \frac{\partial {f}_{i,j,11}}{\partial {x}_{i,j,2}} & \frac{\partial {f}_{i,j,12}}{\partial {x}_{i,j,2}}\\ \frac{\partial {f}_{i,j,1}}{\partial {x}_{i,j,2}} & \frac{\partial {f}_{i,j,2}}{\partial {x}_{i,j,2}} & 1 & \cdot \cdot \cdot  & \frac{\partial {f}_{i,j,11}}{\partial {x}_{i,j,2}} & \frac{\partial {f}_{i,j,12}}{\partial {x}_{i,j,2}}\\ \cdot  & \cdot  & \cdot \cdot \cdot  & \cdot  & \cdot  & \cdot \\ \cdot  & \cdot  & \cdot \cdot \cdot  & \cdot  & \cdot  & \cdot \\ \frac{\partial {f}_{i,j,1}}{\partial {x}_{i,j,11}} & \frac{\partial {f}_{i,j,2}}{\partial {x}_{i,j,11}} & \frac{\partial {f}_{i,j,3}}{\partial {x}_{i,j,11}} & \cdot \cdot \cdot  & 1 & \frac{\partial {f}_{i,j,12}}{\partial {x}_{i,j,11}}\\ \frac{\partial {f}_{i,j,1}}{\partial {x}_{i,j,12}} & \frac{\partial {f}_{i,j,2}}{\partial {x}_{i,j,12}} & \frac{\partial {f}_{i,j,3}}{\partial {x}_{i,j,12}} & \cdot \cdot \cdot  & \frac{\partial {f}_{11}}{\partial {x}_{i,j,12}} & 1\end{array}).$$

Here ${f}_{i,j,1}={[P{M}_{10}]}_{j}$, ${f}_{i,j,2}={[P{M}_{2.5}]}_{j}$, ${f}_{i,j,3}={[{O}_{3}]}_{j}$, ${f}_{i,j,4}={[CO]}_{j}$, ${f}_{i,j,5}={[NO]}_{j}$, ${f}_{i,j,6}={[N{O}_{2}]}_{j}$, ${f}_{i,j,7}={[S{O}_{2}]}_{j}$, ${f}_{i,j,8}={[T]}_{j}$, ${f}_{i,j,9}={[P]}_{j}$, ${f}_{i,j,10}={[WD]}_{j}$, ${f}_{i,j,11}={[WS]}_{j}$, and ${f}_{i,j,12}={[RH]}_{j}$. Similarly, ${x}_{i,j,1}={[P{M}_{10}]}_{j}$, ${x}_{i,j,2}={[P{M}_{2.5}]}_{j}$, ${x}_{i,j,3}={[{O}_{3}]}_{j}$, ${x}_{i,j,4}={[CO]}_{j}$, ${x}_{i,j,5}={[NO]}_{j}$, ${x}_{i,j,6}={[N{O}_{2}]}_{j}$, ${x}_{i,j,7}={[S{O}_{2}]}_{j}$, ${x}_{i,j,8}={[T]}_{j}$, ${x}_{i,j,9}={[P]}_{j}$, ${x}_{i,j,10}={[WD]}_{j}$, ${x}_{i,j,11}={[WS]}_{j}$, and ${x}_{i,j,12}={[RH]}_{j}$.

We solve Eq. 6 for the unknown association coefficients ${\beta }_{i,j}$ for the disease-air pollution model Eq. 1 for a disease *i* in year *j*. Notice that the diagonal elements of the Jacobian matrix ${J}_{i,j}$ are one, while the non-diagonal elements represent confounding effects between the environmental factors. If the environmental factors are independent of each other, then the elements from the non-diagonal elements of the Jacobian matrix ${J}_{i,j}$ will become zero. This reduces ${J}_{i,j}$ to a simple unitary matrix.

We solved Eq. 6 for ${B}_{i,j}$ by matrix inversion of the Jacobian matrix ${J}_{i,j}$, followed by a matix multiplication,

9 $${B}_{i,j}={J}_{i,j}^{-1}{A}_{i,j}.$$

The elements of the matrix ${A}_{i,j}$ and the Jacobian matrix ${J}_{i,j}$ were calculated using the linear regression method^32^. The effectiveness of the model in the analysis of the relationship (matrix ${B}_{i,j}$) between air pollution and disease is evaluated against a set of analytical distribution of air pollution with known association coefficients. The results will be shown in the Results section.

### Disease air pollution risk *R*_*D*_

Starr^34^ measured the fatality risk of activity as total fatalities with respect to total person-hour of exposure to the activity considered. This method leads to a fatality risk as to the statistical probability of fatalities per person-hour of exposure. In this work, we follow Starr^34^ to compute the risk of increased visits for a disease as the statistical probability of the occurrences of positive association coefficients with respect to all association coefficients.

From Eq. 9, we compute and plot distribution of association coefficients ${\beta }_{i,j}$ for each of the 18 diseases over 5 years and 3 age groups of patients. For each disease and each age group of outpatients, there are 35 association coefficients ${\beta }_{i,j}$ (5 years × 7 air pollution factors) for each age group of outpatients. The statistical significance of the association coefficients are tested using the Student’s *t* test^29,32^. The detailed distribution of the values of the association coefficients ${\beta }_{i,j}$ and the P values of the association coefficients for each disease *i* and each age group of outpatients is shown in the Supplementary Materials.

A positive association coefficient ${\beta }_{i,j}$ represents an increase in ${M}_{i,j}$ disease outpatients with respect to an increase in a specific air pollution factor. Hence, the occurrences of total positive and statistically significant association coefficients ${\beta }_{i,j}$ (P-value < 0.05) with respect to all 35 association coefficients ${\beta }_{i,j}$ indicates the probability of increases in the outpatient visits for a disease with respect to all pollutants. We use this probability to describe the risk of disease associated with air pollutants, and is computed as followed:

10 $${R}_{D}=\frac{Total\,number\,of\,occurrences\,of\,positive\,{\beta }_{i,j}\,for\,a\,disease}{Occurrences\,of\,all\,{\beta }_{i,j}\,for\,a\,disease}$$

**Figure 2:**
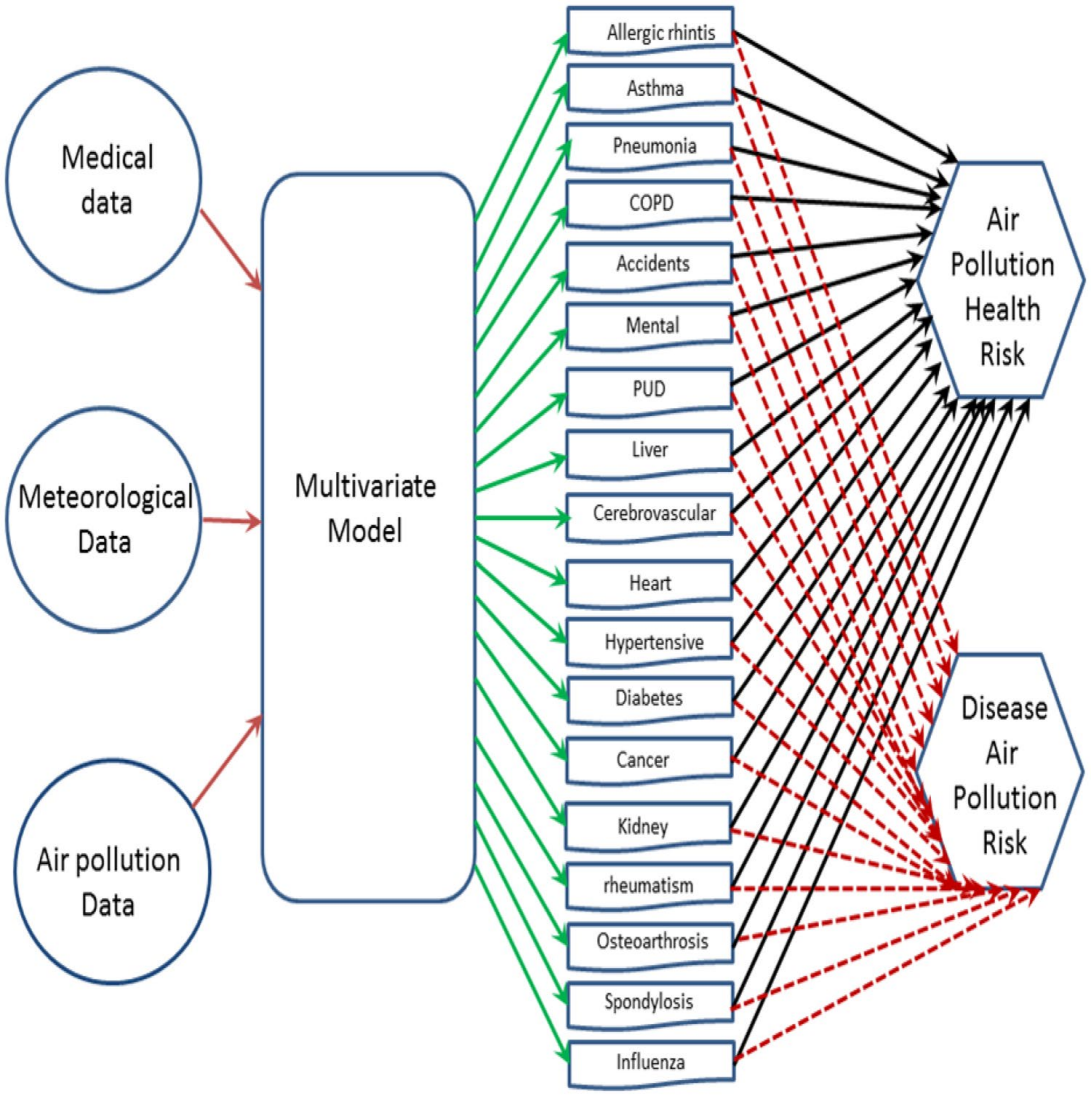
Calculation of the disease air polltion risks (${R}_{D}$), and the air pollution health risks (${R}_{A}$) in this work. The inputs are medical data from Taiwan Landseed Hospital, and air pollution and meteorological data from EPA ambient air monitoring station in Chung-Li. The multivariate model is the diseases-air pollution model used in this work.

Note that the risk in the context of Eq. 10 essentially means the probability associated with an event of interest–for example, the probability of increased outpatient visits for a disease^35^. Figure 2 shows a flowchart of air pollution risks computed in this work.

### Air pollution health risk *R*_*A*_

When Eq. 1 is applied through 18 diseases over 5 years, we compute the probability of statistically significant and positive association coefficients ${\beta }_{i,j}$ (P < 0.05) for each air pollutant with respect to all diseases. Following equation gives air pollution health risk with respect to each air pollutant.

11 $${R}_{A}=\frac{Total\,number\,of\,occurrences\,of\,positive\,{\beta }_{i,j}\,from\,a\,{pollutant}}{Occurrences\,of\,all\,{\beta }_{i,j}\,from\,a\,{pollutant}}$$

### Daily outpatients, air pollution, and meteorological data

The left-hand side of Eq. 1 requires the input of daily outpatient visits for each of the 18 diseases. The data of daily outpatient visits for the 18 diseases from the Taiwan Landseed Hospital were used in this work^29,30^. Each medical record contains the residential location of the outpatient. These residential locations were converted to geographical locations (in longitudes and latitudes) for computing the distances between the residential places of the outpatients to the emission sources. The diseases, coded according to the ICD-9 codes (Table 1), during 2007–2011 were used in this study. The hourly air pollution and meteorological data were obtained from the ambient air monitoring station in Chung-Li operated by the Taiwan Environmental Protection Administration (EPA^36^). The ambient air pollution and meteorological variables were measured at an hourly frequency. The instruments used to make air pollution measurements were calibrated once per day, at the midnight of each day of measurements. The calibration gases were traceable to the United States (US) National Institute of Standards and Technology (NIST). Taiwan EPA strictly follows methods and references set up by the US EPA^37^ to operate a network of 84 automatic ambient air stations that continuously monitor ambient air pollution in Taiwan. A professional instrument company in Taiwan has operated all 84 automatic ambient air stations. Taiwan EPA has also set up another independent procedure to conduct the audit of all instruments and data quality for each of the ambient air stations by another professional instrument company. This mechanism, an independent operator of the instruments and another independent auditor of the instruments, ensure the data quality, references, and the monitoring methods meet the requirements all the time. All monitoring data are calibrated, online, and openly accessible on the English website (click on Data Service on the left column of https://taqm.epa.gov.tw/taqm/en/default.aspx to obtain hourly monitoring data). Taiwan EPA has been widely used in international studies^38^.

These hourly data were used to computed daily maximum values for each variable used in this work. The time-series plots of daily air pollution measurements $P{M}_{10}$, $P{M}_{2.5}$, *O*_3_, CO, $N{O}_{2}$, NO, and $S{O}_{2}$; and temperature data were shown in Wang and Chau^29^. Tests of normality for air pollutants and meteorological variables were also shown in Wang and Chau^29^. Tests of normality for data from 18 diseases are shown in the Supplementary Materials.

## Results

### Verification of the disease-air pollution model

Equation 1 is the core to the air pollution health impact assessment. Hence, we design a set of analytical solutions to test Eq. 1. Following is a list of equations used in this analytical test:

12 $$\begin{array}{rcl}{[P{M}_{10}]}_{k} & = & 30+k\\ {[P{M}_{2.5}]}_{k} & = & 250+k\times \,\sin (2k)\\ {[{O}_{3}]}_{k} & = & 250+k\times \,\sin (3k)\\ {[CO]}_{k} & = & 250+k\times \,\sin (4k)\\ {[NO]}_{k} & = & 250+k\times \,\sin (5k)\\ {[N{O}_{2}]}_{k} & = & 250+k\times \,\sin (6k)\\ {[S{O}_{2}]}_{k} & = & 250+k\times \,\sin (7k)\\ {[T]}_{k} & = & 250+k\times \,\sin (8k)\\ {[P]}_{k} & = & 250+k\times \,\sin (9k)\\ {[WD]}_{k} & = & 250+k\times \,\sin (10k)\\ {[WS]}_{k} & = & 250+k\times \,\sin (11k)\\ {[RH]}_{k} & = & 250+k\times \,\sin (12k)\end{array}$$

Here *k* represent Julian days.

By substituting Eq. 12 into Eq. 1, and let association coefficients ${\beta }_{i,j}$ with respect to each parameter equal to unity, we can write time-series outpatients (OP) for a disease as a fundtion of 12 environmental factors:

13 $$\begin{array}{rcl}O{P}_{i,k} & = & {[P{M}_{10}]}_{i,k}+{[P{M}_{2.5}]}_{i,k}+{[{O}_{3}]}_{i,k}+{[CO]}_{i,k}+{[NO]}_{i,k}\\  &  & +\,{[N{O}_{2}]}_{i,k}+{[S{O}_{2}]}_{i,k}+{[T]}_{i,k}+{[R]}_{i,k}+{[WD]}_{i,k}+{[WS]}_{i,k}+{[RH]}_{i,k}\end{array}$$

**Figure 3:**
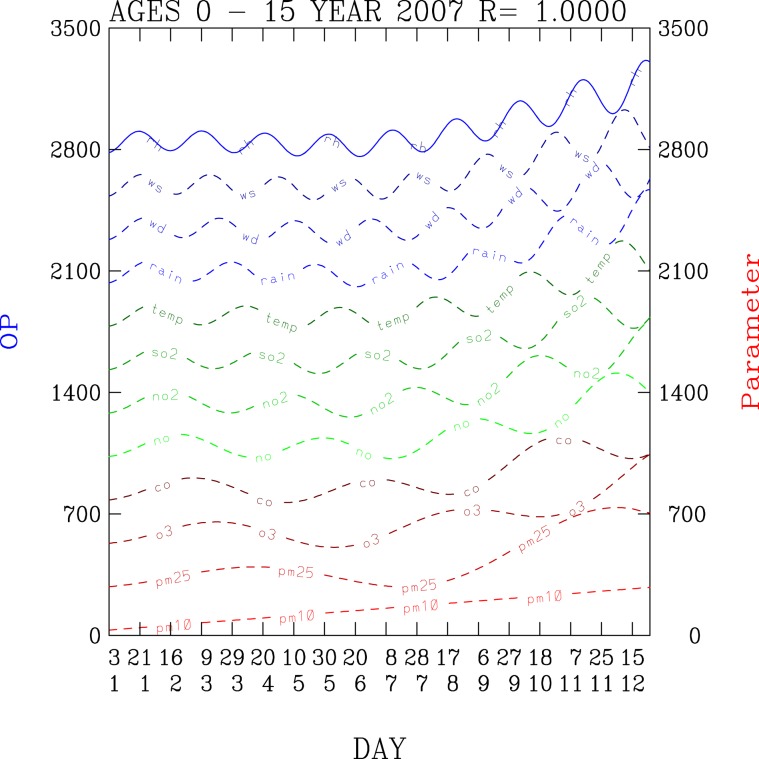
Time-series distribution of 12 environmental factors and total outpatients for a disease. The magnitudes of the OP are increased as the contribution from each of the environmental factors are gradually added.

Hence, Eq. 13 shows an analytical distribution of daily outpatient numbers OP with respect to the analytical environmental factos given by Eq. 12. Figure 3 shows time series distribution of outpatients computed according to the analytical solutions, shown in Eq. 13, as each of the 12 analytical functions is included in the outpatient equation for a given year (here shown as 2007).

With the time series OP (from Eq. 13) and 12 parameters (Eq. 12), we compute association coefficients ${\beta }_{i,j}$ as shown in Eq. 1. Table 2 shows results of calculated associate coefficients, and comparisons with the analytical results. The calculated and analytical results are almost the same, verifying the algorithms we use to compute association coefficients ${\beta }_{i,j}$.

**Table 2:** List Computed and Analytical Association Coefficients *β*.

*β*	Computed	Analytical Solution
*β*_12_	1.00007451	1
*β*_11_	1.00012779	1
*β*_10_	1.00016189	1
*β*_9_	1.00018036	1
*β*_8_	1.00018334	1
*β*_7_	1.00016534	1
*β*_6_	1.00014186	1
*β*_5_	1.00013232	1
*β*_4_	1.00010610	1
*β*_3_	1.00005054	1
*β*_2_	1.00002706	1
*β*_1_	1.00000393	1
*β*_0_	−0.33797091	0

**Figure 4:**
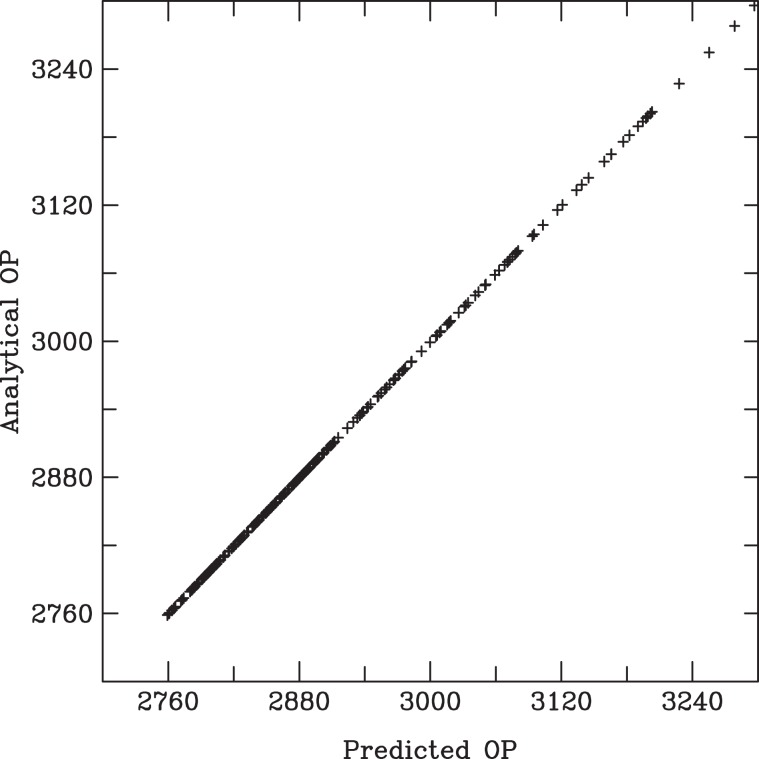
A scattered plot distribution of predicted OP (x-axis) versus analytical OP (y-axis).

The association coefficients $\beta$ shown in Table 2 were calculated from the daily analytical outpatient numbers given by q. 12. These $\beta$ can then be used in Eq. 1 to predict daily time-series outpatient numbers. Figure 4 compares predicted daily outpatient numbers with analytic outpatient numbers. The comparisons show a good agreement between the predicted and analytical outpatient numbers. These results verify the algorithms used in computing the association coefficients from given input daily outpatients numbers and air pollution and meteorological data.

### Air pollution source burden per outpatient

**Figure 5:**
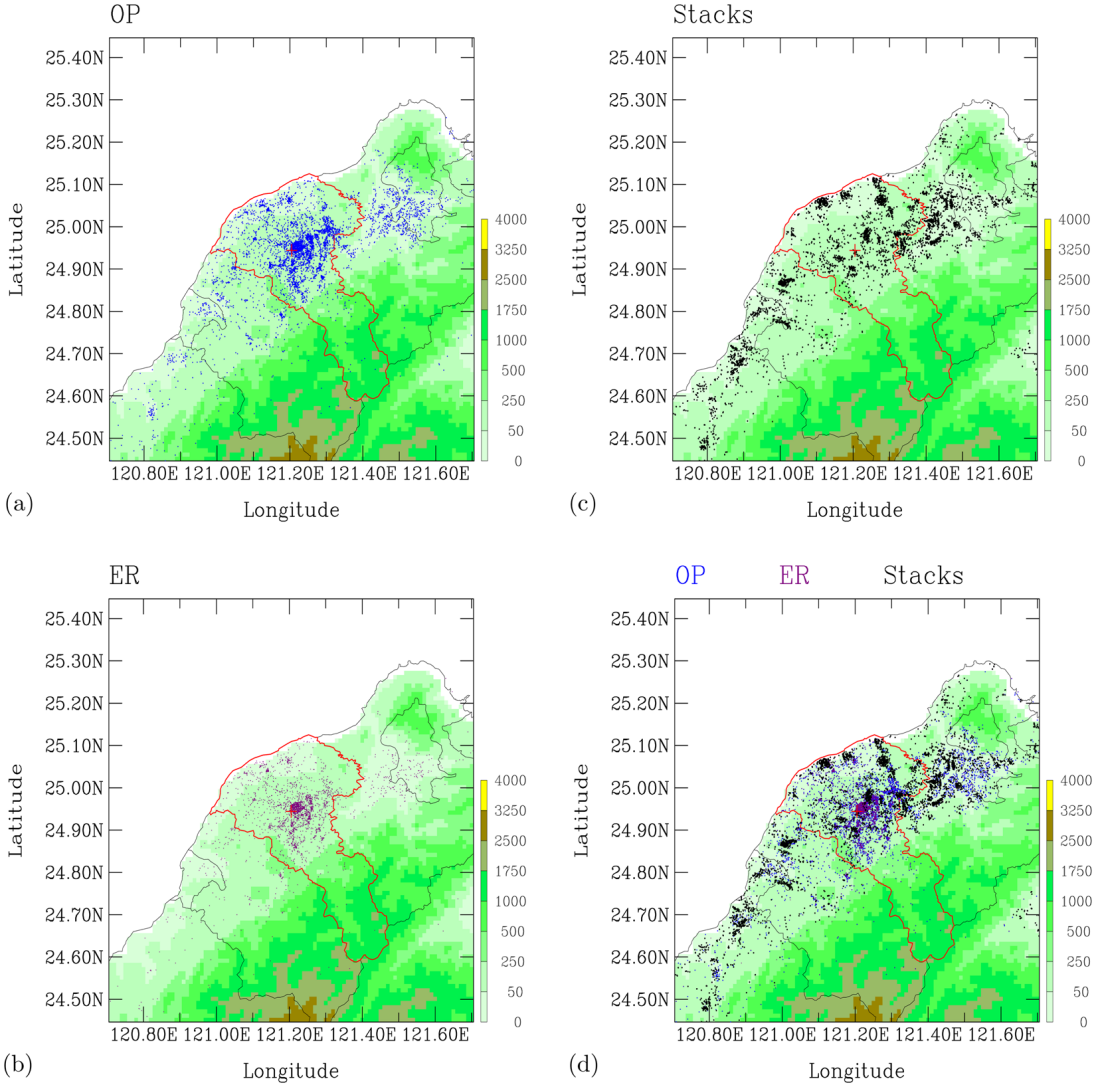
Spatial distribution of outpatients for the respiratory diseases and industrial emission source during 2007–2011. (**a**) Outpatients. (**b**) Outpatients from emergency room visits. (**c**) Registered industrial source emissions. (**d**) Composite of outpatients and industrial emission sources. Shaded colors indicate terrain heights, in units of meters.

**Figure 6:**
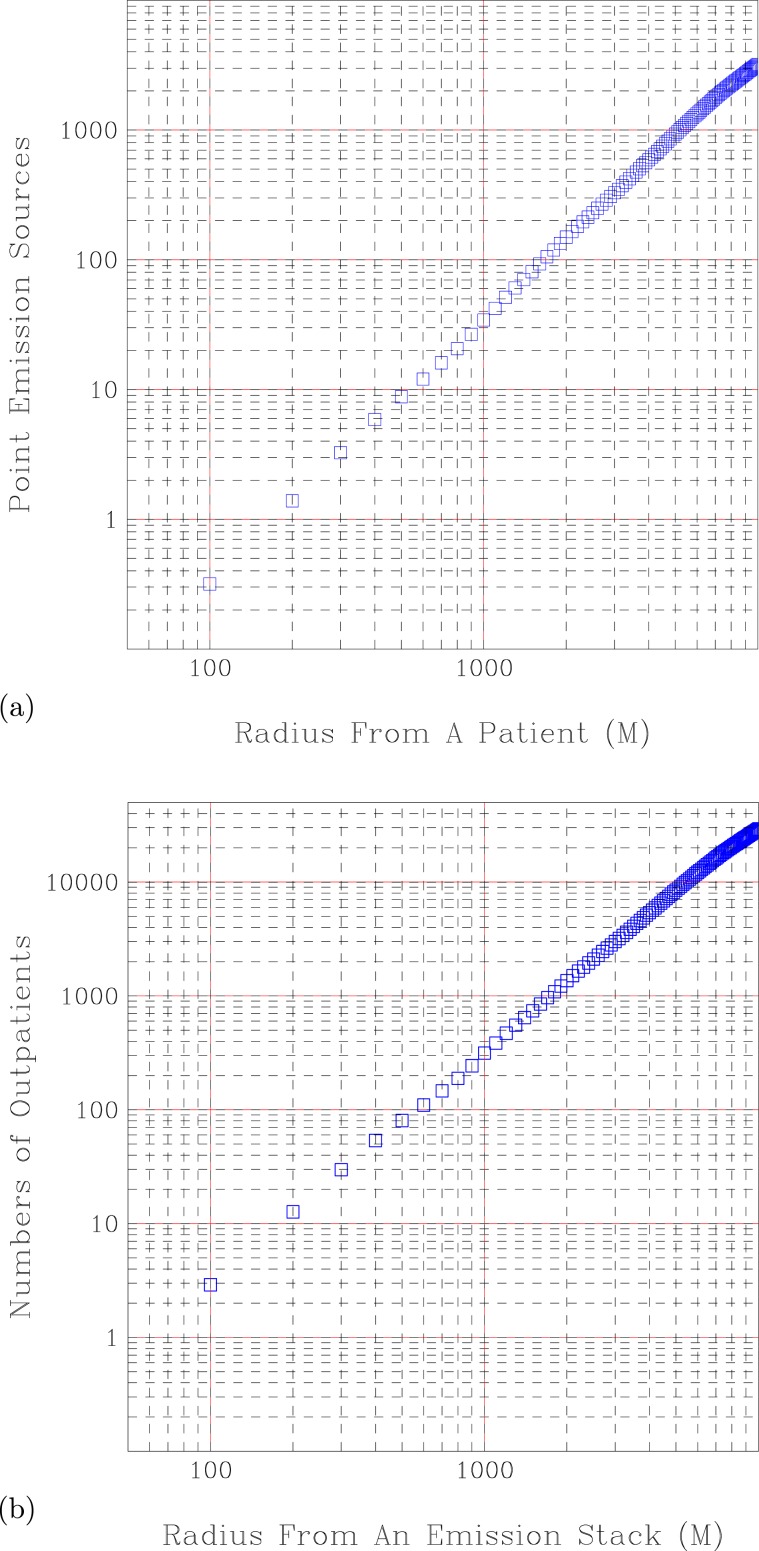
A scattered plot analysis of spatial closeness between emission sources and outpatients during 2007–2011. (**a**) Averaged number of industrial emission sources versus distance from an outpatient. (**b**) Averaged number of Outpatients versus distance from an industrial emission source.

The environmental burden of pollution sources in the studied area can be appreciated by examing the spatial distribution between the emission sources and outpatients for diseases. Figure 5(a) shows the spatial distribution of outpatients for the diseases of the respiratory system during the period 2007–2011, while Fig. 5(b) shows a distribution of outpatients with emergency visits. These two figures show a total of 239,610 of outpatients for the respiratory diseases during 2007–2011. Figure 5(c) shows the spatial distribution of the registered industrial emission sources registered on the online governmental system Taiwan Emissions Data Set^39^. There are 26,132 industrial emissions shown in this figure. Figure 6(d) overlaps emission sources with all outpatients (normal and emergency room visits). It is clear that the spatial distribution of outpatients and air pollution sources are very close to each other.

How close are the emission sources to the location of the residency of outpatients? We calculated the number of registered industrial emission sources with respect to the distance to each outpatient. Figure 6(a) shows a log distribution between the averaged number of industrial emission sources and the distance to an outpatient. On average, there is an industrial emission source within 180 meters of an outpatient. The industrial emissions for an outpatient are 0.334 at 100 meters, 36.135 sources at 1000 meters of distance, and 3370.175 emission sources at 10 km of distance. Figure 6(a) exhibits a log-linear relationship between the emission sources and distances to the patient. The following equation can approximate this relationship:

14 $$y={C}_{1}\times ({x}^{{r}_{1}})$$

Here *C*_1_ = 3.31 × 10^−5^, *r*_1_ = 2.002, *y* is the number of emission source, and *x* is the distance to an outpatient. Verification of this equation at *x* = 1000 meters, predicts *y* = 33.551 emission source. The real emission sources at 1000 meters are 36.135. Hence the errors from this equation are 7.2%.

On the other hand, how many outpatients are exposed to registered industrial emissions? Figure 6(b) shows a log distribution of averaged outpatient numbers with respect to the distance to an emission stack from 100 meters to 10 kilometers. Figure 6(b) also exhibits a log-linear relationship between the outpatient numbers and distance to the industrial emission source. There are about 3 outpatients at 100 distance from an industrial emission source, and 30,236 outpatients at 10 kilometers from an industrial emission source. The following equation can approximate this relationship:

15 $$y={C}_{2}\times ({x}^{{r}_{2}})$$

Here *C*_2_ = 0.0003, *r*_2_ = 2.002.

Hence, on average, there are 3 outpatients from an industrial emission source at 100 meters of distance; and one industrial emission source from an outpatient at 150 meters of distance.

### Disease distribution in outpatients: an age-based perspective

Table 3 shows a list of total outpatient visits according to disease and year during 2007–2011. A total of 1.78 million outpatient visits during this period. The most substantial 26.46% of outpatients were resulting from the diseases of the circulatory system (7.63% from the cerebrovascular diseases, 7.76% from the heart diseases, and 11.07% from the hypertensive diseases). The second largest outpatients of 19% were from the diseases of the musculoskeletal system and connective tissue. Followed by 13.80% of outpatients from the diseases of the genitourinary system, and 10.32% from the accidents. A 10.12% from the diseases of the respiratory system, and 9.31% from the diseases of endocrine disorders (diabetes mellitus). A 4.95% from the diseases of the digestive system, and 4.68% from the diseases of mental disorders (anxiety, dissociative and somatoform disorders). A 2.95% from the diseases of malignant neoplasm (cancers), and 0.89% from influenza.

**Table 3:** List of Total Outpatient Visits According to Disease and Year.

Disease	2007	2008	2009	2010	2011	Total	Percent
Respiratory system							10.12
Allergic Rhinitis	5118	6803	8413	9041	9021	38396	2.15
Asthma	7779	7475	7910	8988	12458	44610	2.50
Pneumonia	7878	7036	7066	6989	9051	38020	2.13
COPD	11061	11225	12159	12082	13076	59603	3.34
Accidents	37228	36703	34782	37147	38264	184124	10.32
Mental disorders	15437	15078	16770	16776	19490	83551	4.68
Digestive system							4.95
Peptic ulcer	9106	7839	7817	6453	6807	38022	2.13
Chronic liver/cirrhosis	11222	10309	10247	9310	9239	50327	2.82
Circulatory system							26.46
Cerebrovascular disease	22024	25963	28388	29434	30334	136143	7.63
Heart disease	24151	26173	28107	26365	33671	138467	7.76
Hypertensive disease	38511	42048	42591	40240	34089	197479	11.07
Diabetes mellitus	28662	29382	32705	35325	39906	165980	9.31
Malignant neoplasm	9219	10103	9457	10098	13655	52532	2.95
Genitourinary system	44595	50552	50367	49677	50965	246156	13.80
Musculoskeletal system							19.00
Other disorders of soft tis.	11440	11814	19203	22400	28963	93820	5.26
Osteoarthrosis/allied dis.	25645	26063	22152	26545	32890	133295	7.47
Spondylosis/allied dis.	16355	21102	23518	24707	26075	111757	6.27
Influenza vaccine injection	164	54	11836	2334	1507	15895	0.89
Yearly Total	317816	338247	365578	364923	397003	1780070	100.00

These statistics reveal that the diseases of the circulatory systems, connecting tissues in bones, and genitourinary systems account for nearly 60% of all outpatients; and account for 80% of all outpatients when outpatients of accidents and respiratory systems are added.

**Figure 7:**
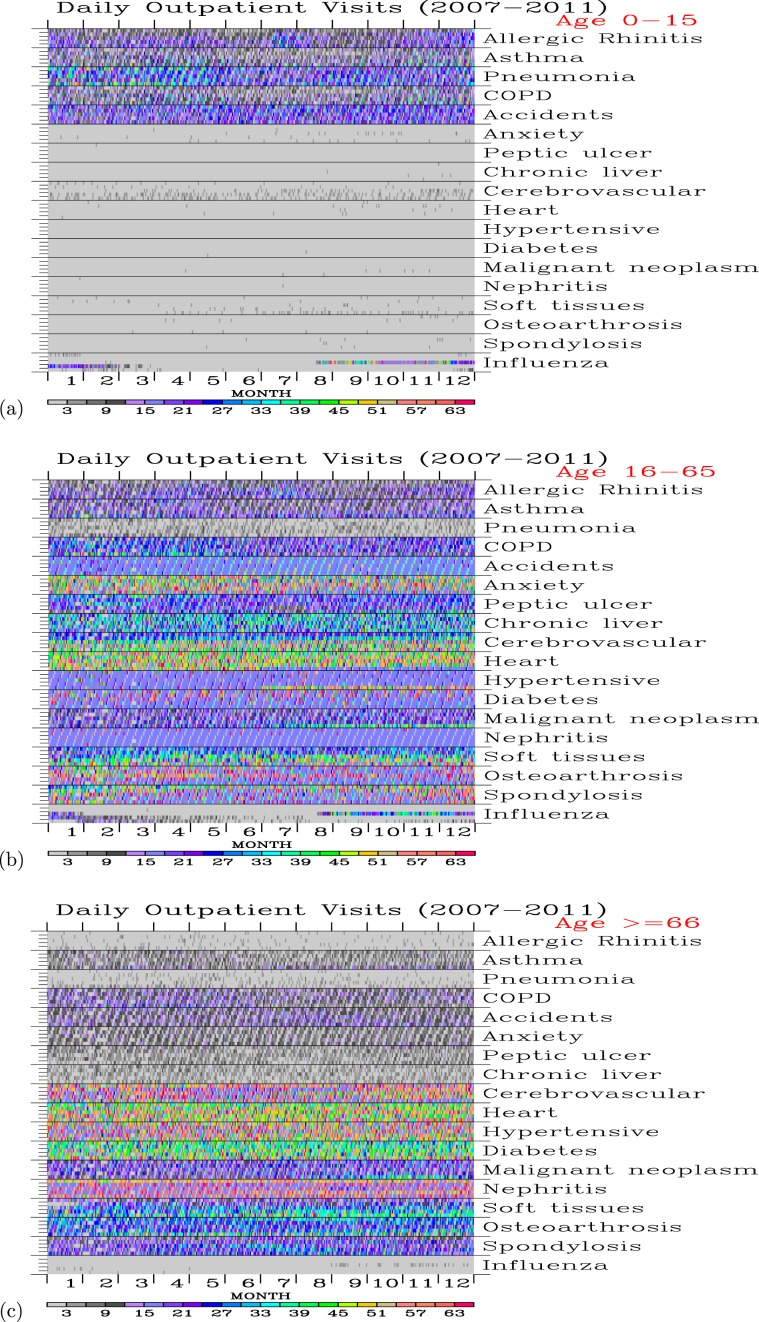
Daily counts of outpatient visits (shaded colors) for the (**a**) 0–15, (**b**) 16–65, and (**c**) above 65 year-old age group of people for each of the ICD-9 diseases during the 2007–2011 period.

Figure 7 shows daily outpatients for the 18 diseases from 2007 to 2011 and each age group of outpatients. The youngest group outpatients (0–15 years old) were mostly suffered from the diseases of respiratory systems and accidents. The leading causes of diseases of the respiratory systems were allergic rhinitis and pneumonia than, followed by asthma and the COPD. There were few visits for the young bodies from the diseases of mental disorders, digestive system, circulatory system, diabetes, cancer, genitourinary system, musculoskeletal system.

For the 16–65 years old outpatients, the diseases have spread to the entire body. The leading causes of diseases were resulting from mental disorders (anxiety, dissociative, and somatoform disorders), the circulatory system, and the musculoskeletal system. The leading causes of the diseases of the respiratory system are allergic rhinitis, asthma, and COPD. Pneumonia is not a significant cause of the respiratory disease as the 0–15 years old outpatients.

For the most senior group of the outpatients (higher than 65 years old), the leading causes of diseases were resulting from the circulatory system, diabetes, genitourinary system, and musculoskeletal system. Diseases of the digestive system and respiratory system, accident, and mental disorder are comparatively low. Apparently, for those who can survive into old ages, their mental disorders are less a problem than the physical functionality of a body.

### Disease air pollution risks

**Figure 8:**
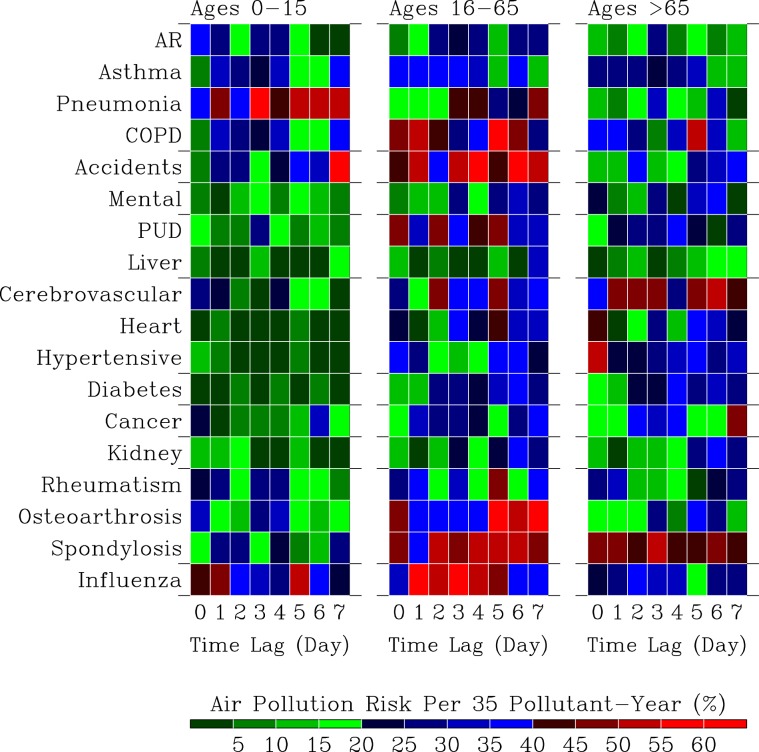
Disease air pollution risks ${R}_{D}$. Distribution of ${R}_{D}$ with respect to a spectrum of 18 diseases and 8 time-lags scenarios in 3 age groups of outpatients.

Figure 8 shows computed the distribution of disease air pollution risks *R*_*D*_ with respect to a spectrum of 18 diseases and 8 time-lags scenarios between the hospital visits for diseases and environmental factors in 3 age groups of outpatients.

For the 0–15 years old patients, higher than 40% of air pollution risks are mostly associated with the diseases of the respiratory system (pneumonia), accidents, and influenza. Immediate air pollution risks are mostly associated with AR (35–40%), pneumonia (35–40%), and influenza (40–45%). One-day delay air pollution risks are associated with pneumonia (45–50%) and influenza (45–50%). Pneumonia and influenza are two diseases that exhibit the most persistent occurrence of risks that are mostly higher than 40%.

We note that the air pollution risks for the following diseases are below 20% for the 0–15 years old patients in all 8 scenarios: mental (no mental problem yet), chronic liver and cirrhosis (liver is still excellent), heart and hypertensive (heart working very good and blood vessels are very clean), diabetes (no abnormality in body sugar production, regulation, and accumulation), and kidney (the genitourinary system is in good shape).

The occurrences of elevated air pollution risks for ages 0–15 outpatients are ranked as pneumonia (6), influenza (3), and accidents (1). The most sensitive diseases for air pollution risks for young outpatients is influenza. A total of 3 diseases out of 18 diseases showing high air pollution risks (${R}_{D} > 40 \%$).

For 0–15 years old outpatients, diseases with low health risks (${R}_{D} < 20 \%$) are mostly from the mental, digestive system (PUD, liver), circulation system, and cancer, and kidney. Diseases with high health risks (${R}_{D} > 40 \%$) are mostly from the respiratory system and influenza. Key air pollutants are $P{M}_{2.5}$, *O*_3_, NO, $P{M}_{10}$, and $N{O}_{2}$.

For the 16–65 years old outpatients, there exist more widespread occurrence of air pollution risks to the entire body than the 0–15 years old outpatients. The model shows that diseases of the respiratory system (pneumonia and COPD), accident, one disease of the digestive system (PUD), two diseases of the circulatory system (cerebrovascular and heart), three diseases of the musculoskeletal system, and influenza all exhibit higher the 40% of air pollution risks. The immediate impacts of elevated air pollution risk (${R}_{D} > 40 \%$) occur in COPD, accidents, PUD, OA, and spondylosis. The 1-day delay impacts of elevated air pollution risks (${R}_{D} > 40 \%$) occur in COPD, accidents, and influenza. For 2-day to 6-day delay impacts of air pollution risks (${R}_{D} > 40 \%$), these are diseases mostly in pneumonia, COPD, accidents, PUD, cerebrovascular, heart, rheumatism, OA, spondylosis (2-day delay), and influenza. Up to 7-day delay of air pollution risks (${R}_{D} > 40 \%$) are seen in pneumonia, accidents, OA, and spondylosis.

The occurrences of elevated air pollution risk (${R}_{D} > 40 \%$) for the 16–65 years old outpatients are ranked as accidents (7), spondylosis (7), COPD (5), OA (4), influenza (5), pneumonia (3), PUD (4), cerebrovascular (2), heart (1), and rheumatism (1). This group of outpatients is most sensitive to air pollution risks for diseases of COPD, accidents, PUD, OA, and spondylosis. For the 16–65 years old outpatients, a total of 10 diseases out of 18 diseases analyzed here showing high air pollution risks (${R}_{D} > 40 \%$), exhibiting the highest distribution of disease air pollution risks.

For the outpatient with ages higher than 65 years old, elevated air pollution risks (${R}_{D} > 40 \%$) mainly distributed in the diseases of the respiratory system (COPD), diseases of the circulatory system (heart, hypertensive, and cerebrovascular diseases), cancer, and musculoskeletal system (spondylosis disease). Immediate impact (0-day delay) of elevated air pollution risks (${R}_{D} > 40 \%$) concentrate on the diseases of the circulatory system (heart and hypertensive), and spondylosis disease. The 7-day delayed effects of air pollutions risks (${R}_{D} > 40 \%$) appear in the diseases of cerebrovascular, cancer, and spondylosis. The most prevalent diseases with elevated air pollution risks (${R}_{D} > 40 \%$) are cerebrovascular diseases and spondylosis diseases.

The occurrences of elevated air pollution risks (${R}_{D} > 40 \%$) for the elderly outpatients are from diseases of spondylosis (8), cerebrovascular (6), cancer (1), hypertensive (1), heart (1) and COPD (1).

The diseases of the circulatory system (cerebrovascular) and the musculoskeletal system (spondylosis) of the elderly outpatients exhibit the most sensitive and the most persistent of up to 7 days of the air pollution risks. A total of 6 diseases show the occurrence of high air pollution risks (more than 40%) from 18 diseases.

### Air pollution health risks

**Figure 9:**
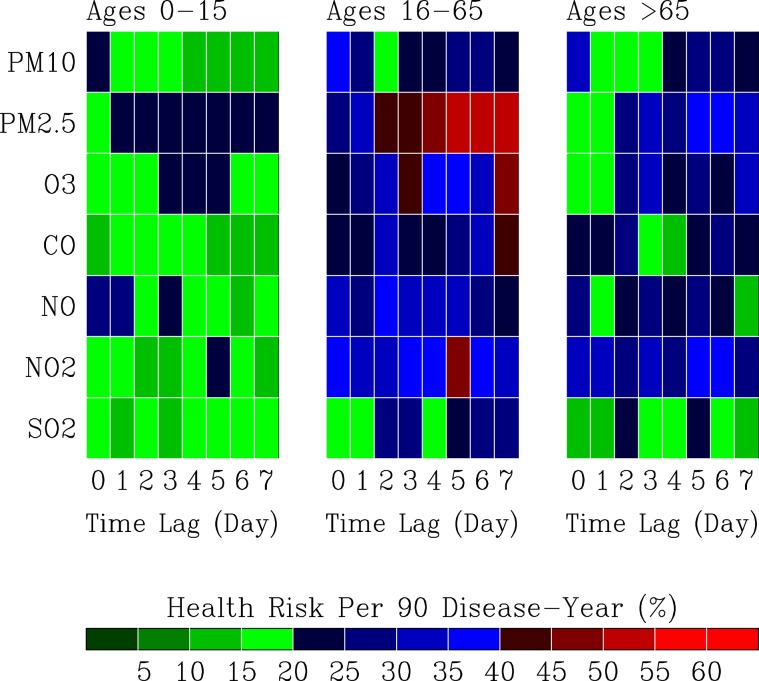
Air pollution health risks ${R}_{A}$. Distribution of ${R}_{A}$ with respect to 7 pollutants and 8 time-lag scenarios in 3 groups of outpatients.

Figure 9 shows air pollution health risks *R*_*A*_ with respect to 7 pollutants and 8 time-lag scenarios in 3 groups of outpatients. For young outpatients (0–15 years old), occurrences of the 20%–40% of health risks at time lags of 1–7 days are mostly associated with $P{M}_{2.5}$, NO, *O*_3_, and $N{O}_{2}$. The immediate impacts (0-day delay) on health risk (20%–40%) are associated with $P{M}_{10}$ and NO.

For the 16–65 years old outpatients, elevated health risks (${R}_{A} > 40 \%$) mostly occur at time lags of 1–7 days and are associated with $P{M}_{2.5}$, *O*_3_, $N{O}_{2}$, and CO. Occurrences of immediate impacts on health risks (*R*_*A*_ = 20%–40%) are associated with $P{M}_{10}$, $P{M}_{2.5}$, *O*_3_, CO, NO and $N{O}_{2}$.

For the elderly outpatients (>65 years old), immediate health risks (*R*_*A*_ = 20–40%) are mostly associated with $P{M}_{10}$, CO, NO, and $N{O}_{2}$. The health risks ${R}_{A}$ at time lags of 1–7 days are prevaingly associated with $N{O}_{2}$, followed by associations with $P{M}_{2.5}$, *O*_3_, CO, NO, $P{M}_{10}$, and $S{O}_{2}$.

Above results reveal that $P{M}_{2.5}$ is the most hazardous air pollutant from the analysis of outpatient data. The second most hazardous air pollutant is *O*_3_, followed by $N{O}_{2}$ and CO. Air pollutants $P{M}_{10}$ and $NO$ are most active than other air pollutants in producing immediate (0-day) delay health impacts (*R*_*A*_ = 20%–40%) for all outpatients.

### Health risks: a whole body perspective

**Figure 10:**
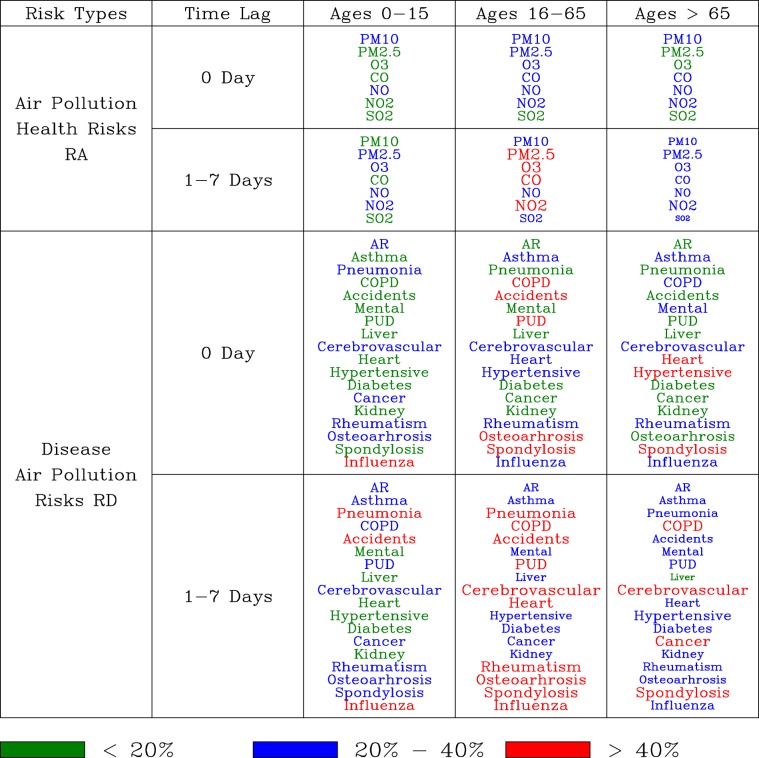
Distribution of air pollution health risks (${R}_{A}$) and disease health risks (${R}_{D}$) with respect to immediate (0 Day) and delayed (1–7 Days) time responses in 3 age groups of outpatients.

Our analysis revealed that the health risks of air pollution depends on types of air pollutants, physical ages of outpatients, and the response of the body to changing air pollution concentrations. Figure 10 summarizes the distribution of air pollution health risks (${R}_{A}$) and and disease health risks (${R}_{D}$) with respect to immediate (0 Day) and delayed (1–7 Days) time responses in 3 age groups of outpatients.

For the 0–15 years old outpatients, $P{M}_{10}$ and NO contribute to the immediate impact of the health risks, while $P{M}_{2.5}$, *O*_3_, NO, and $N{O}_{2}$ contribute to the time lags of 1–7 days of health risks. Diseases of immediate and with 20%–40% health risks are AR, pneumonia, cerebrovascular, cancer, rheumatism, OA, and influenza (${R}_{D} > 40 \%$). Diseases with time lags of 1–7 days and with the 20%–40% health risks are AR, asthma, pneumonia (high health risks), COPD, accidents (${R}_{D} > 40 \%$), PUD, cerebrovascular, cancer, rheumatism, OA, spondylosis, and influenza (${R}_{D} > 40 \%$). As the body response time increases from 0-day to 1–7 days, representative air pollutants for diseases change from $P{M}_{10}$ and NO to $P{M}_{2.5}$, *O*_3_, NO, and $N{O}_{2}$. Changes of diseases with increases in health risks and increases in body response times are asthma, pneumonia, COPD, accidents, and spondylosis. Hence, $P{M}_{2.5}$, *O*_3_, and $N{O}_{2}$ correspond to increases in health risks for asthma, pneumonia, COPD, accidents, and spondylosis.

For the 16–65 years old outpatients, air pollutants with the immediate health risks of 20%–40% are $P{M}_{10}$, $P{M}_{2.5}$, *O*_3_, NO, and $N{O}_{2}$. Diseases with immediate and health risks of 20%–40% are ahthma, COPD (${R}_{D} > 40 \%$), accidents (${R}_{D} > 40 \%$), PUD (${R}_{D} > 40 \%$), cerebrovascular, heart, hypertensive, rheumatism, OA (${R}_{D} > 40 \%$), spondylosis (${R}_{D} > 40 \%$), and influenza. The highest health risks (${R}_{A} > 40 \%$) are associated with the increases in time lags of 1–7 days and are associated with $P{M}_{2.5}$, *O*_3_, CO, and $N{O}_{2}$. The main changes of air pollutants with increasing health risks are $P{M}_{2.5}$, *O*_3_, CO, $N{O}_{2}$, and $S{O}_{2}$ (*R*_*A*_ = 20%–40%). Increases in time responses to time lags of 1–7 days resulting in the increases of health risks of diseases of AR, penumonia (${R}_{D} > 40 \%$), mental, liver, cerebrovascular (${R}_{D} > 40 \%$), heart (${R}_{D} > 40 \%$), diabetes, cancer, kidney, rheumatism (${R}_{D} > 40 \%$), and influenza (${R}_{D} > 40 \%$).

For the elderly outpatients, $P{M}_{10}$, CO, NO, and $N{O}_{2}$ are associated with the immediate health risk of 20%–40%, while all 7 seven air pollutants are associated with the 1–7 days delay of health risks (*R*_*A*_ = 20%–40%). Air pollutants $P{M}_{2.5}$, *O*_3_, and $S{O}_{2}$ have become imporant in the health risks (*R*_*A*_ = 20%–40%) as the time lags increase to 1–7 days. The corresponding changes of diseases with increases in health risks are AR, pneumonia, COPD (${R}_{D} > 40 \%$), accidents, PUD, cerebrovascular (${R}_{D} > 40 \%$), diabetes, cancer (${R}_{D} > 40 \%$), kidney, and OA.

Figure 10 shows that the most hazardous air pollutants for health risks of ${R}_{A} > 40 \%$ are $P{M}_{2.5}$, *O*_3_, CO, and $N{O}_{2}$. Diseases with ${R}_{D} > 40 \%$ of health risks are pneumonia, COPD, accidents, PUD, heart, hypertensive, cerebrovascular, heart, cancer, rheumatism, OA, spondylosis, and influenza. The time lags of 1–7 days of health risks are accompanied by more diseases and higher risks than the immediate health risks. The 16–65 years old outpatients are most susceptible to elevated health risks for more diseases across the body from air pollution than the other two groups of outpatients.

For the 0–15 years old outpatients, concurrent occurrences of elevated health risk of ${R}_{D} > 40 \%$ is influenza for immediate health risks; and diseases of pneumonia, accidents, and influenza for time lags of 1–7 days of health risks. For the 16–65 years old outpatients, concurrent occurrences of elevated health risk of ${R}_{D} > 40 \%$ are diseases of COPD, accidents, PUD, OA, and spondylosis for the immediate health risks; and diseases of pneumonia, COPD, accidents, PUD, cerebrovascular, heart, rheumatism, osteoarthrosis, spondylosis, and influenza for time lags of 1–7 days of health risks. For the elderly outpatients, concurrent occurrences of elevated health risk of ${R}_{D} > 40 \%$ are diseases of heart, hypertensive, and spondylosis for the immediate health risks; and diseases of COPD, cerebrovascular, cancer, and spondylosis for time lags of 1–7 days of health risks. Hence, Fig. 10 validates our hypothesis that air pollution has widespread and concurrent impact on diseases from various organs in the body.

## Discussion

Our results of the elevated health risks are consistent with previous findings that diseases of the respiratory and circulatory systems were found to be associated with the air pollution^23,40–42^. Long-term air pollution exposure was found to be an important risk factor for cancers^43,44^.

For the association of diseases of the digestive system with air pollution, studies showed that air pollution affects the gut microbiota and linked to the inflammatory disease^45–49^, and confirmed from experiments with mice^47,50,51^. Inhaled air pollutants are quickly cleared from the lungs and transported by the mucociliary into the intestine^52^. Cytotoxin substance produced by gut bacteria *Campylobacter pylori* were shown to present in patients with PUD than those with chronic gastritis only^53^. The human gut is populated with as many as 100 trillion microbes^54^. The human-gut microbiota interactions producing host-microbiota metabolism, signaling, and immune-inflammatory processes that physiologically connect the gut, liver, muscle, and brain^55^, affecting health and diseases^56,57^, and is now an innovative method for fighting against cancer^58^. Hence, elevated health risks of PUD found in this work concurs with evidence found in previous works.

For NO, which is the Molecule of the Year 1992^59^, participates in the widespread signaling of cellular and organ function in the body^60^. NO is a unique signaling molecule in the vascular system^61^ that touches nearly all areas of life^62^. Also, Maher *et al*.^13^ found that $N{O}_{2}$ and $P{M}_{2.5}$ were positively associated with the incidence of dementia in London, England.

For the links between air pollution and brain and accidents, studies showed the pathways for air pollutants to attack the brain central nervous system include (1) by entering the nose and travel through the olfactory bulb to enter the brain; (2) by affecting the lining of the nasal epithelium, causing the release of the inflammatory cytokines that damage the brain; (3) by entering the lung, activating the release of the proinflammatory cytokines that transported by the blood to the brain area to cause adverse brain effects^20^. As such, exposure to air pollution is a contributor to the decline of cognitive function^63^. Functional near-infrared spectroscopy (fNIRS) measurements of brain activity of train drivers confirm variations of brain waves during various stages of driving processes^64^. Neuropsychological tests showed that exposure to CO leads to dysfunctions in memory, attention and concentration, tracking skills, visuomotor skills, visuospatial planning and processing^65^. CO exposure was tested to show a highly significant deficit in careful driving skills^66^. Experiments have confirmed that abnormally low numbers of hypocretin-producing neurons in the brain, resulting in the reduced levels of protein hypocretin^67^, which in turns signal the bone marrow to increase levels of white blood cells that can migrate to arterial walls, leading to the formation of plaque and atherosclerosis^68^.

Immune pathways that link fever and gut immunity are shown in a study demonstrating trafficking of immune cells to enhance immune response and fight infection^69^. T lymphocyte cell receptor called *α*-4 integrins when combined to the heat-shock proteins (hsps), produced by T cell during the fever condition, was able to pull together hsps activated T cells and stick on the blood-vessel walls to infection sites^69^. This *α*-4 integrins-related pathway could have important roles in inflammation and gut immunity.

Our analysis shows that the most frequent occurrences of diseases associated with elevated health risks of ${R}_{D} > 40 \%$ are diseases of spondylosis (in bones), COPD (in the lung), accidents (in the brain), cerebrovascular (in blood vessels), and influenza (brain linked to raising body temperature to kill invaded pathogen associated with air). The concurrence of these diseases in various organs of the body indicate the connections that had occurred within the body.

What mechanisms that can cause such a wide spread of impacts on diseases with increasing air pollution levels? Why the concurrent increase in the diseases of the musculoskeletal system, digestive system, and influenza? In a whole body perspective, the widespread distribution of diseases in various part of body organs indicate that the body immune system and the brain has been affected by the increased in concentrations of air pollution. In the context of the immune system^70^, the neurogenic inflammation causes the diseases of the musculoskeletal system triggered by the immune system’s response to the foreign toxins from inhaled air pollutants^71^. Air pollution caused oxidative damage and inflammation and resulted in the cytotoxic responses in the body^72–76^. Air pollution was found to cause inflammation, leading to accelerated bone loss and increased risk of bone fracture and osteoporosis^77^. Exposure to air pollution is associated with oxidative damage to DNA and lipids in humans^78^.

Hence, the inflammation processes are the responses of the immune system to external air pollution. Cytokines, mediators (messengers) involved in cell growth and activation, are produced during the processes of inflammation and immunity responses in the body^79,80^. Concentrations of pro-inflammatory cytokines such as tumor necrosis factor (TNF)-*α* ($TNF-\alpha$), interleukin (IL)-6 (IL-6), chemokine macrophage inflammatory protein 2 (MIP-2), and NO were measured in the cell culture where macrophages of mice were exposed to $P{M}_{2.5}$ and $P{M}_{10}$^73^. Pro-inflammatory cytokines $TNF-\alpha$, IL-1, IL-6, granulocyte macrophage colony stimulating factor (GM-CSF), and chemokines IL-8 are abundant in patients with rheumatoid arthritis (RA)^79^. The cardiovascular diseases, resulting from the plaque accumulation on the vascular wall, are associated with the active IL-1*β* produced by vascular wall cells and lesional leukocytes^81^. Cytokines IL-1, IL-6, IL-8, IL-17, leukocyte inhibitory factor (LIF), TNF-*α*, interferon (IFN)-*γ* (INF-*γ*), produce cartilage destruction^82^. Hence, chemicals that are able to inhibit the pro-inflammatory cytokines IL-1, TNF-*α*, interferon (IFN)-*γ* (INF-*γ*), IFN-*β*; to stimulate the production of anti-inflammatory cytokines IL-4, IL-6, IL-10, IL-13, transforming growth factor (TGF)-*β* and insulin-like growth factor (IGF)-1 are potentially good for OA therapy^82^. Since IL-1 caused an increase in DNA damage of chondrocytes in OA cartilage^83^, inhibition of IL-1 is proposed as a strategy for the treatment of RA^84^. Impacts of inhaled toxins such as smoke were found to be associated with the loss of bone mineral density and risk of hip fracture^85,86^.

Therefore, the first line of defense against air pollution is skin and mucous membranes^87–89^. The second line of defense against air pollution is phagocytes, antimicrobial substances, natural killer cells (NK cells), inflammation, and fever. The third line of defense against invading air pollution are B lymphocytes, T lymphocytes, and Antigen-presenting cells (APCs)^70^. If further studies confirm our results shown here, then the degradation of the immune system are severe health risks that were overlooked before. Indeed, studies have shown excessive antibiotic usage in Taiwan^90^, consistent with elevated pollution combined with high density of the population.

In the context of quality of air means the quality of life^91^, the closeness of outpatients and industrial emission sources reveal very groom pictures on the quality of health for people living in an industrial city, as shown in this work. This work was deeply inspired by the seminal work of John Snow on the analysis of the 1854 London cholera outbreak^92,93^. The impacts of air pollutants in biological organisms are profound and heritable, as shown from mice placed 1-km downwind of steel mills produced 1.5- to 2.0-fold of heritable DNA mutations in the pups^6^, and other pathophysiological studies reviewed in this work. The strength of this work is that we can learn more about the impacts of industrial emissions on public health if we study all diseases associated with people as they exposed, breathed, and aged^22,25,26^.

The limitation of this study is that more physiological evidence is needed to further understand the mechanisms hidden in the mountain of health and environmental data. New milestones in the health risk assessment of air pollution have been achieved in recent years. These milestones were propelled by the advancements in new tools such as computational hardware (e.g. GPU computers), diversified observational data^7^, non-conventional environmental data obtained and powered by the development of open-source smart sensors^94^, experimental methods^6^, artificial intelligence^95^, and big data analysis for public health^93^. Application of these new tools can better quantify the health impact of air pollution on a finer spatial-temporal resolution than the environmental data currently available by sparse measurements.

## Conclusions

In this work, we showed a very polluted industrial city, characterized by between 3 outpatients per 100 meters of an industrial emission source and an industrial emission source per 180 meters to an outpatient. A total of 1.78 million outpatients over a spectrum of 18 diseases had visited the Landseed Hospital for medical services during 5 years of 2007–2011. We calculated that the most hazardous air pollutants are (in health risk order) $P{M}_{2.5}$, $N{O}_{2}$, $P{M}_{10}$, *O*_3_, CO, and NO.

In the whole body perspective, the diseases of the respiratory system, the musculoskeletal system, and the circulatory systems exhibit the highest air pollution risks. Diseases of the musculoskeletal system reveal the degradation of the immune system and central nervous systems that have been constantly bombarded by the imported toxic materials as people continuously exposed and breathed in a very polluted atmospheric environment. This impact of air pollution on the body immune system is an important finding of this work.

### Accession codes

All data used in this work are openly available at 10.6084/m9.figshare.7217459.

## Supplementary information

Supporting information

Supporting information 2

Supporting information 3
