# Supplementary material for: An association between air pollution and daily most frequently visits of eighteen outpatient diseases in an industrial city
Source: Sci Rep. 2020 Feb 11;10:2321. doi: 10.1038/s41598-020-58721-0 (PMC7012860; doi:10.1038/s41598-020-58721-0)
Supplement: Supplementary file 1 — Supporting information [file 41598_2020_58721_MOESM1_ESM.pdf]

An association between air pollution and daily most frequently visits of eighteen outpatient diseases in an industrial city: Supporting information S1 Appendix

Tang-Tat Chau
Department of Family Medicine, Taiwan Landseed Hospital, Ping-Jen, Taiwan

Kuo-Ying Wang
Department of Atmospheric Sciences, National Central University, Chung-Li, Taiwan

Short title: AIR POLLUTION HEALTH RISKS

Abstract. In this S1 Appendix, we present tests of normality of outpatient data for diseases of mental disorders, the circulatory system, diabetes mellitus, malignant neoplasm, The genitourinary system, musculoskeletal system, and influenza.

Mental Disorders

Figure 1.

Outpaitent for mental disease are normally occur for people with ages older than 16 years old. The frequency distribution of patient visit shows good agreement with the normal distribution (Figure 1).

Diseases of the Digestive System

Figure 2.

Frequency of outpatient visits for peptic ulcer are close to the normal distribution for 16-65 and over 66 years-old group of people (Figure 2).

Figure 3.

Frequency distribution for outpatients with chronic liver and cirrhosis disease show close resemblence to normal distribution for 16-65 and over 66 year-old outpaitents (Figure 3).

Diseases of The Circulatory System

Figure 4.

Frequency distribution for cerebrovascular outpatients are very close to normal distribution for 16-65 and over 66 years old of people (Figure **??**).

Figure 5.

Frequency distribution for outpatients also show good agreement with normal distribution for 16-65 and over 66 years old of people (Figure 5).

Figure 6.

Frequency distribution for hypertensive outpatients exhibit good agreement with normal distribution for 16-65 and over 66 years old of people (Figure 6).

Endocrine Disorders

Figure 7.

Frequency distribution for diabetes outpatients exhibit pattern with very close to normal distribution for 16-65 and over 66 years old of outpatients (Figure 7).

Malignant Neoplasm

Figure 8.

Normal distribution of outpatient visits also occur for cancer disease (Figure 8).

Diseases of The Genitorurinary System

Figure 9.

Frequency distribution for kindney disease appears to be dominated by a sigle peak, followed by two minor peaks of outpaitent visits (Figure‘9).

Diseases of The Musculoskeletal System And Connectiove Tissue

Figure 10.

Frequency distribution for soft tissue closely resemble to the pattern of normal distribution for 16-65 and over 66 years old of outpatients (Figure 10).

Figure 11.

The frequency distribution of outpaitents for bone and joint disease are remarkably close to the pattern of normal distribution for 16-65 and over 66 years old of people (Figure 11).

Figure 12.

Frequency distribution for spinal joint disease agree very well with the normal distribution for 16-65 and over 66 years old of people (Figure 12).

Influenza Vaccine Injection

Figure 13.

Figures

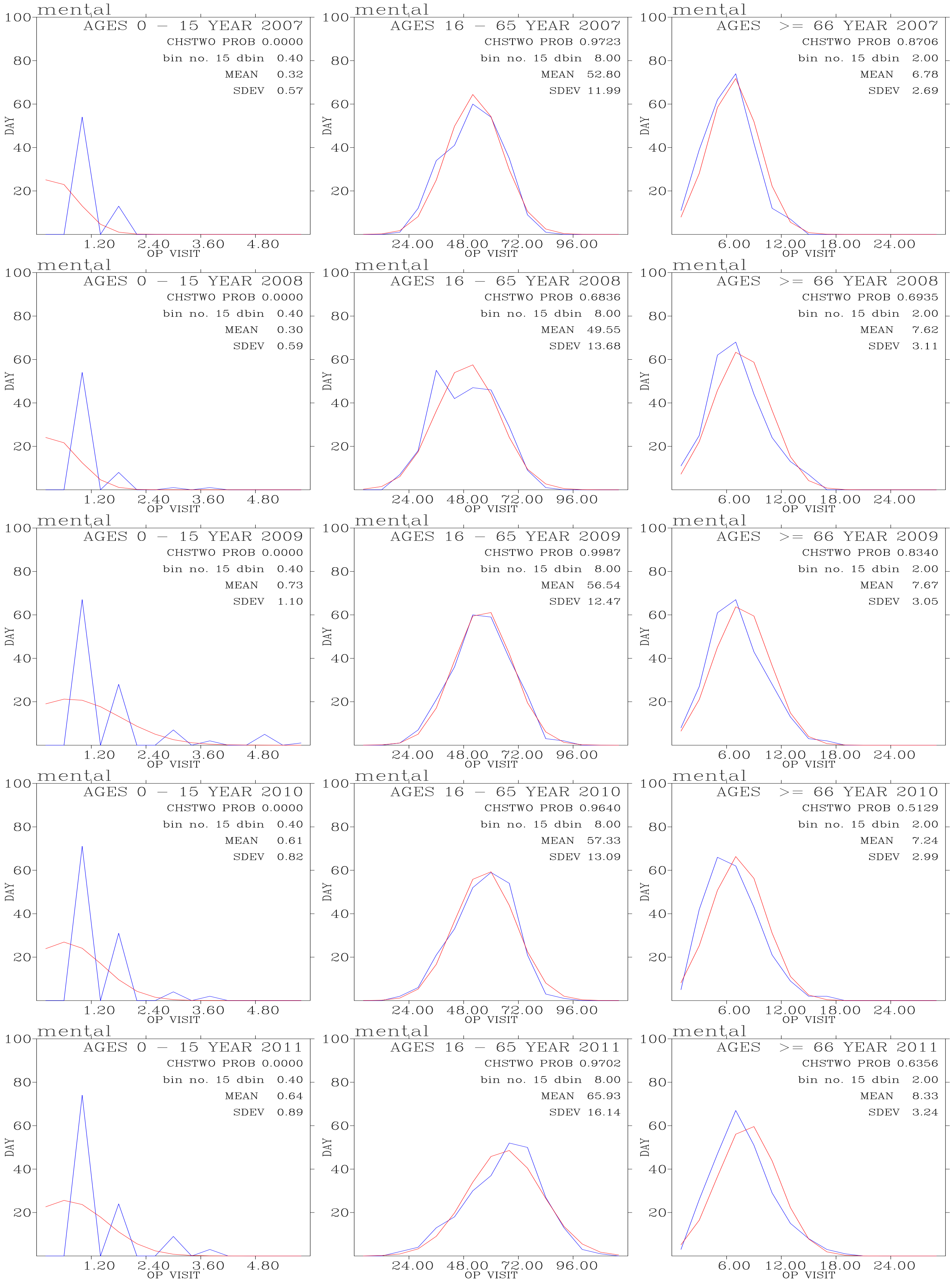

Figure 1. Test of normality distribution for anxiety op.

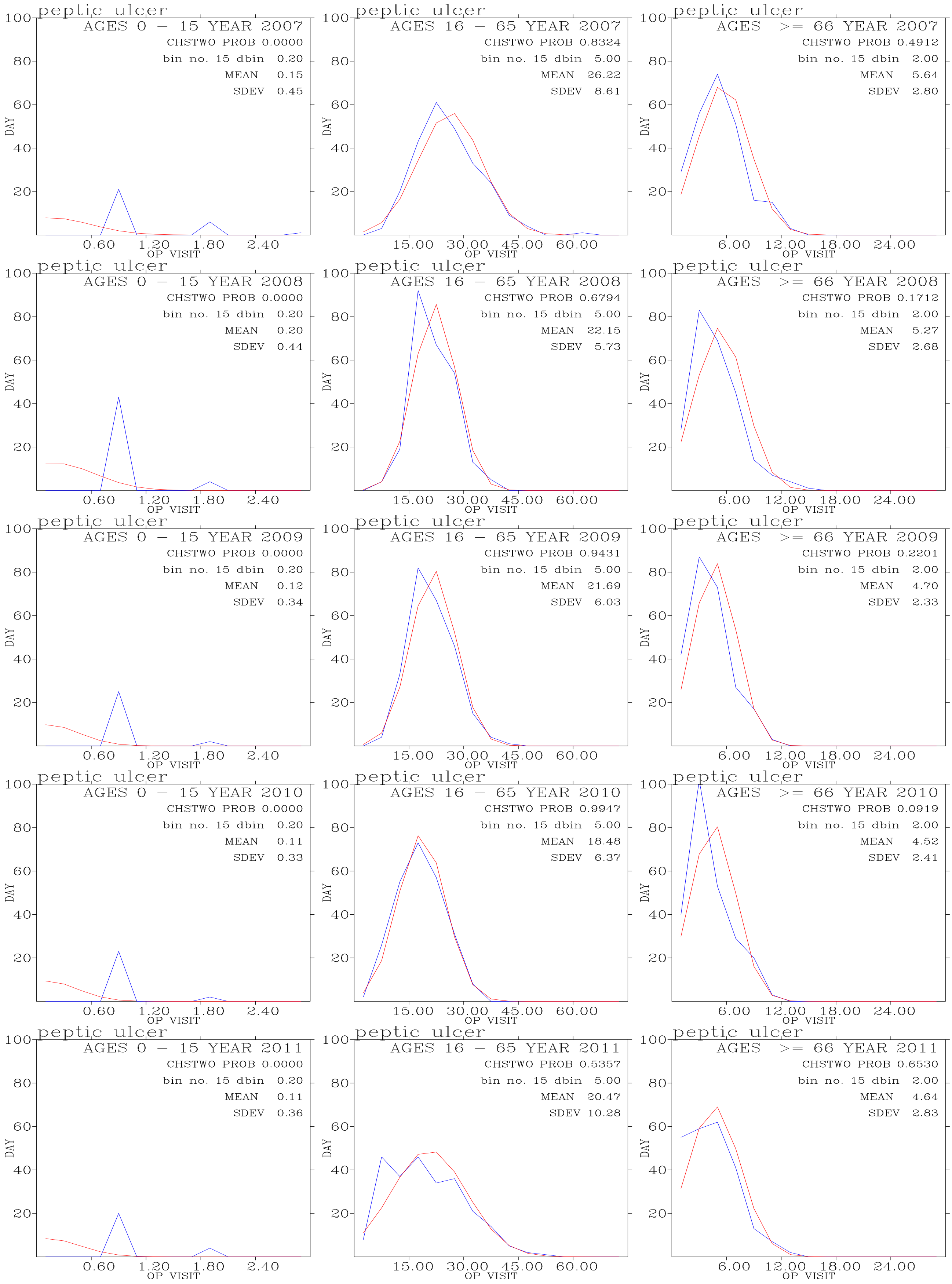

Figure 2. Test of normality distribution for peptic ulcer op.

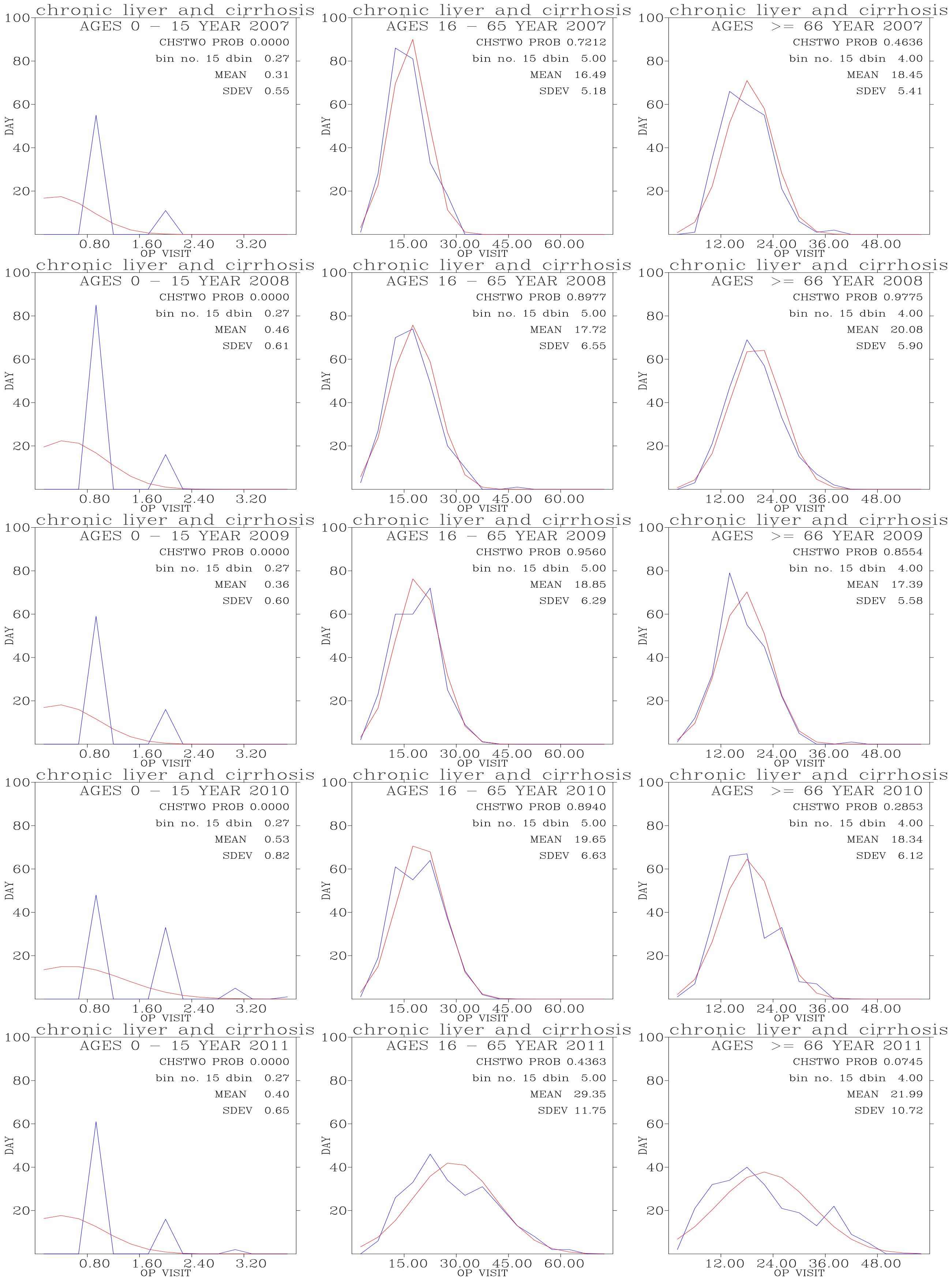

Figure 3. Test of normality distribution for chronic liver disease op.

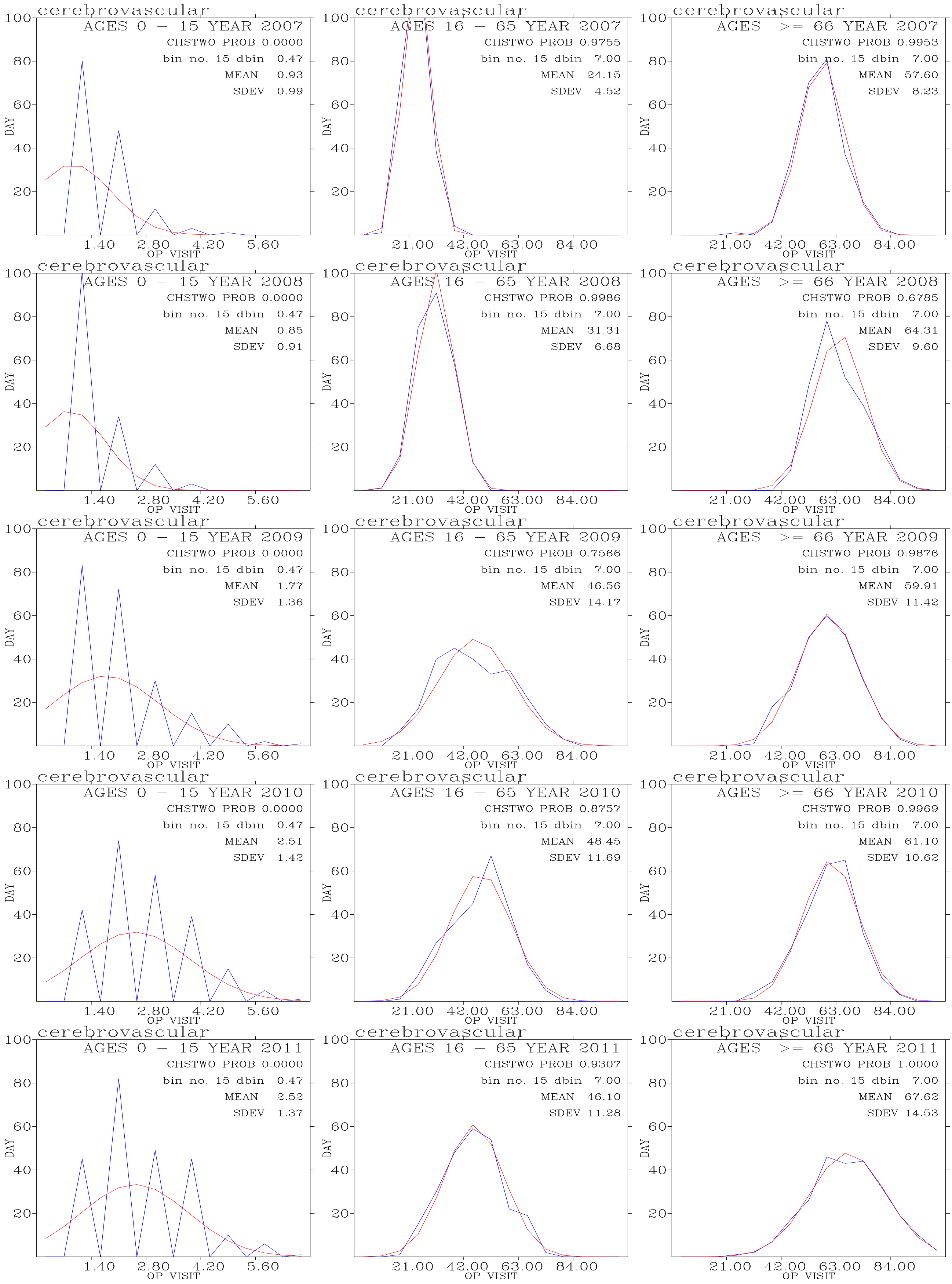

Figure 4. Test of normality distribution for cerebrovascular disease op.

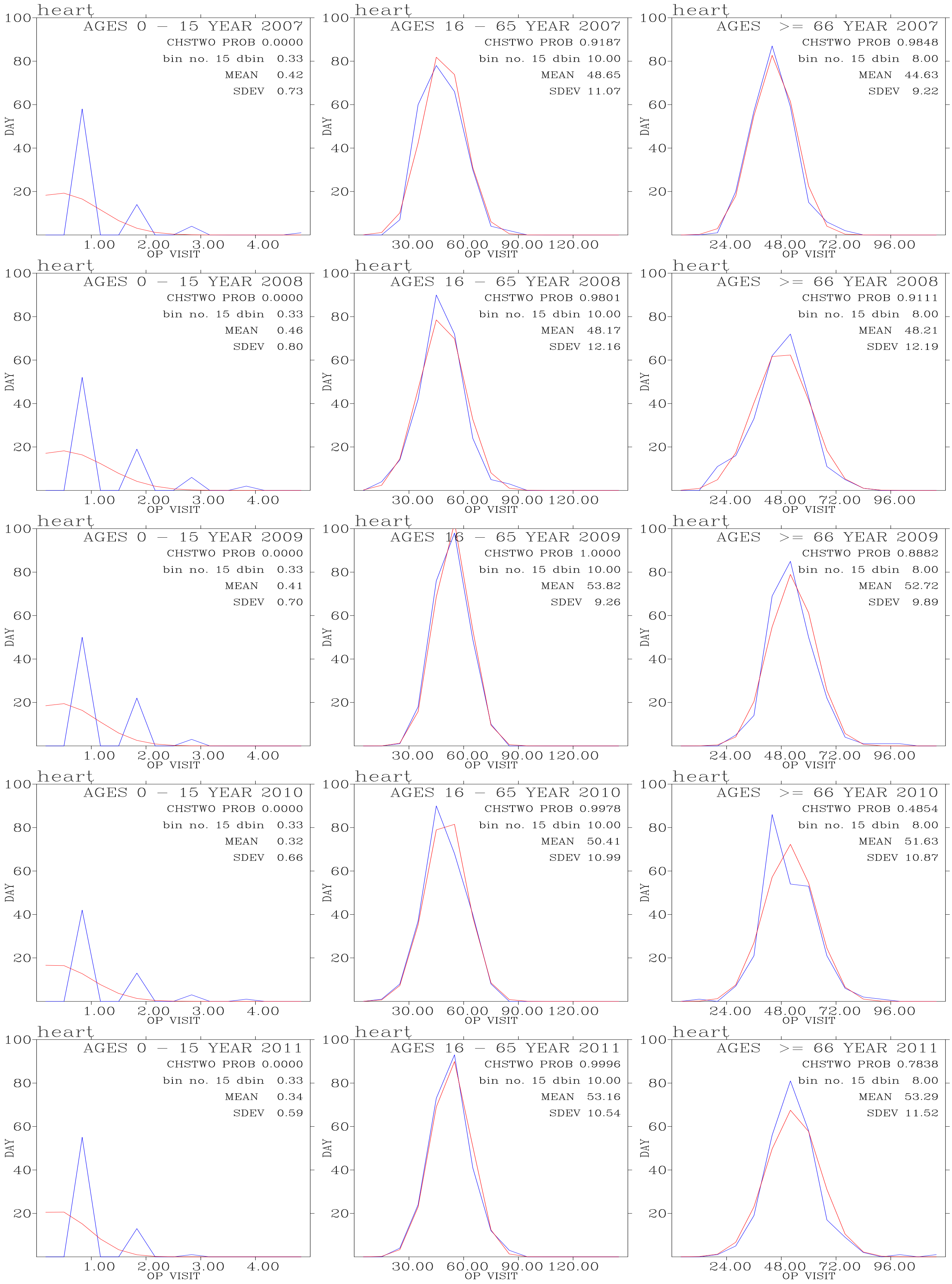

Figure 5. Test of normality distribution for heart disease op.

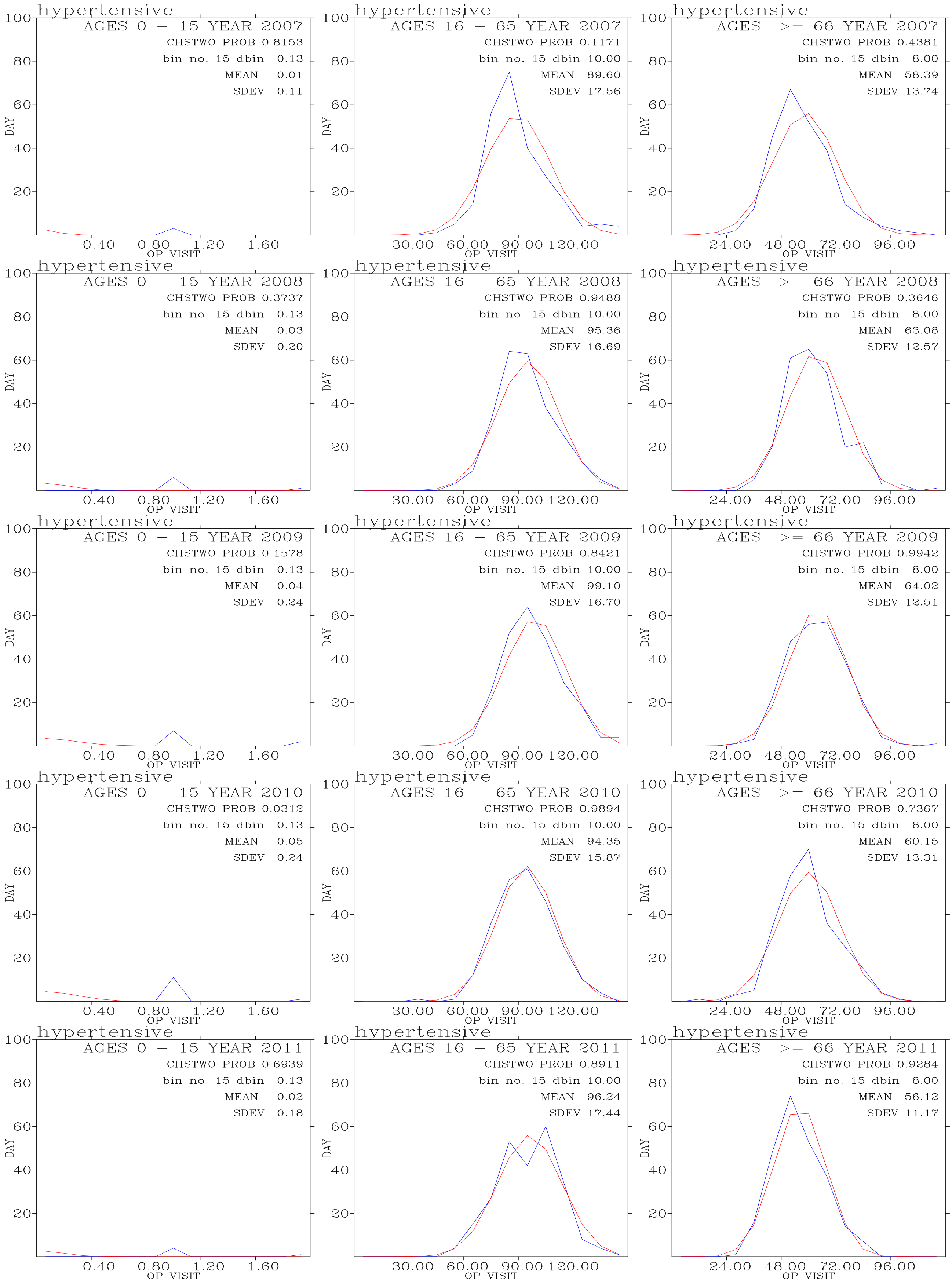

Figure 6. Test of normality distribution for hypertensive disease op.

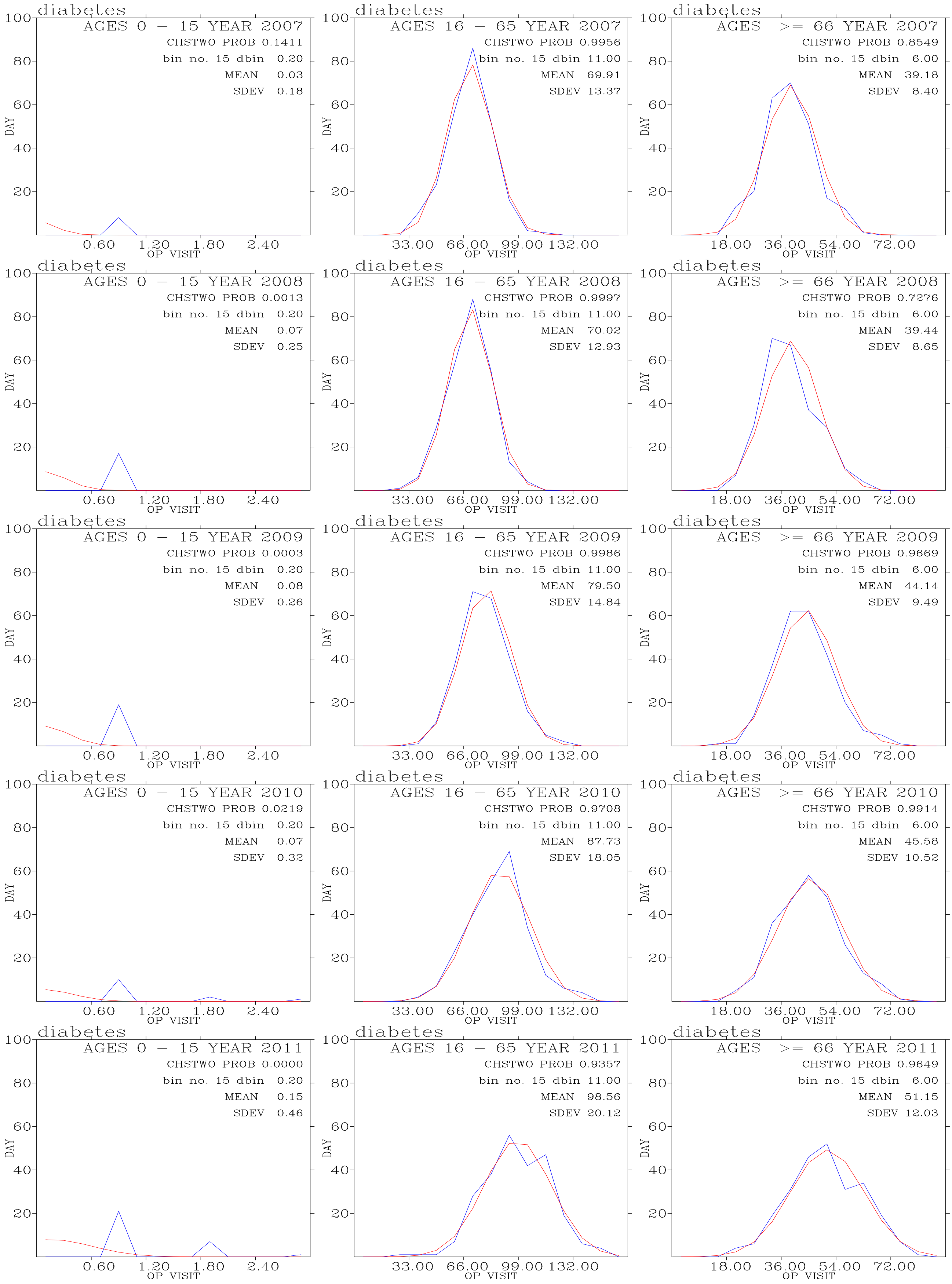

Figure 7. Test of normality distribution for diabetes disease op.

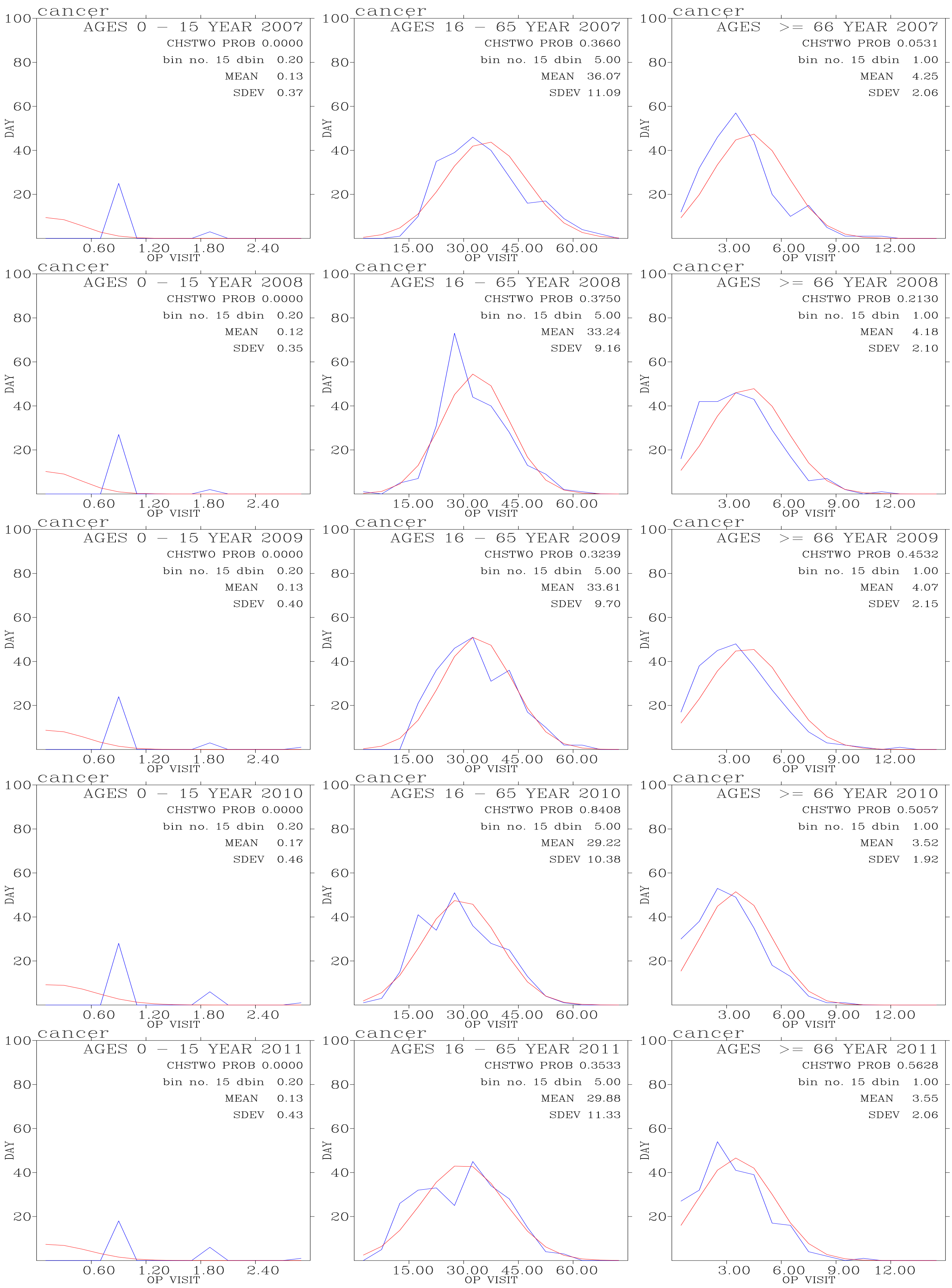

Figure 8. Test of normality distribution for cancer op.

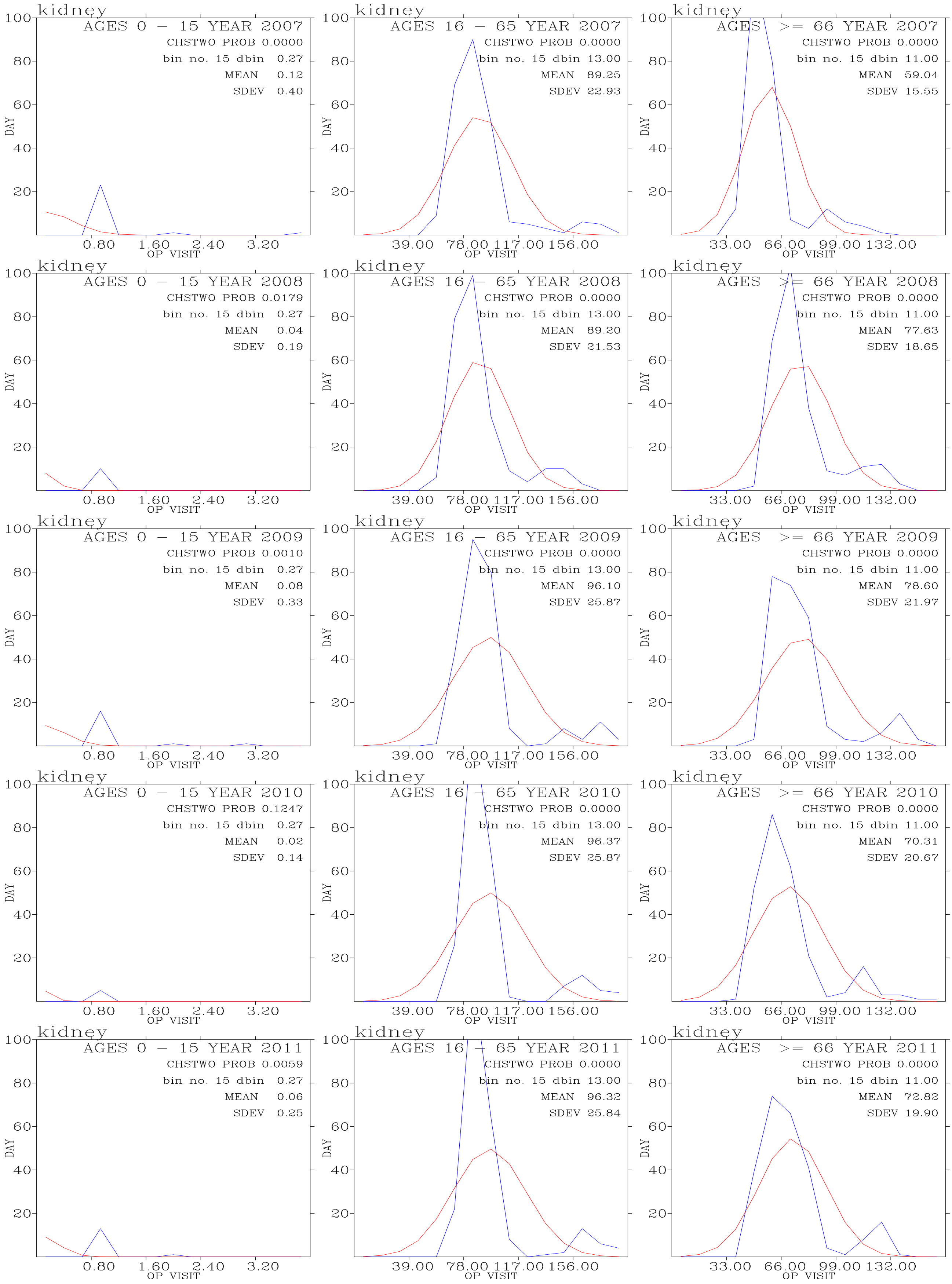

Figure 9. Test of normality distribution for nephritis disease op.

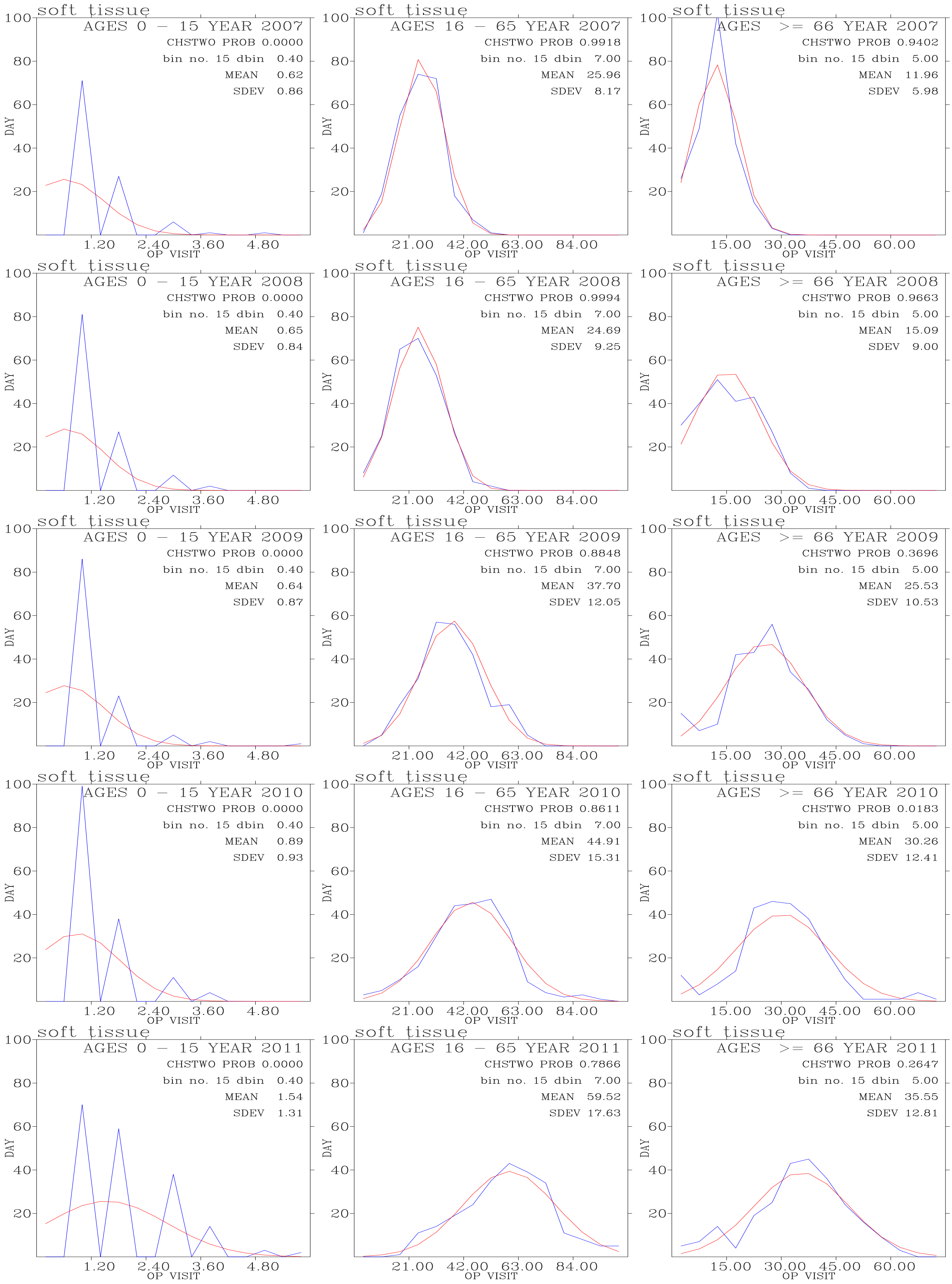

Figure 10. Test of normality distribution for soft tissue disease op.

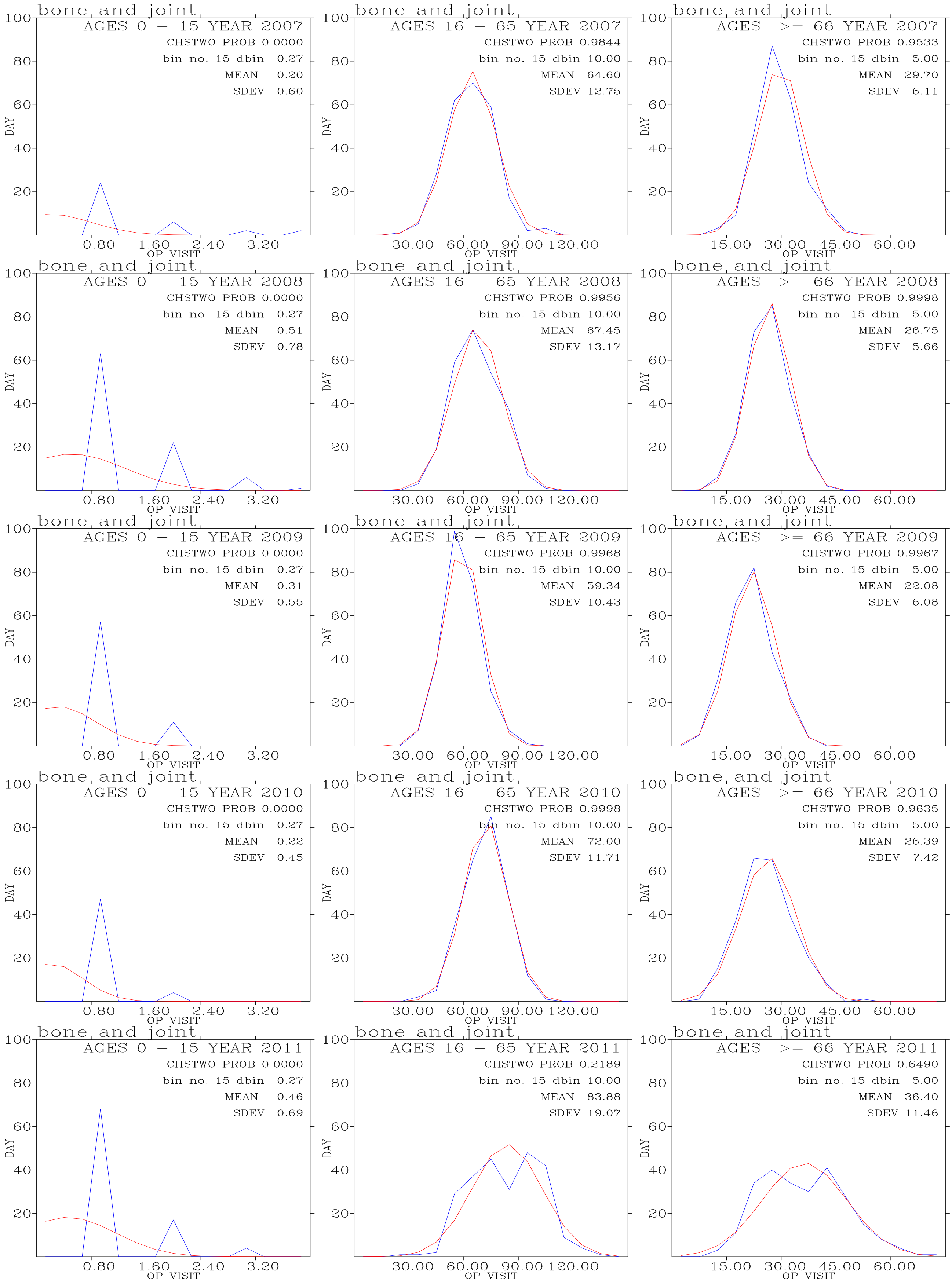

Figure 11. Test of normality distribution for osteoarthrosis disease op.

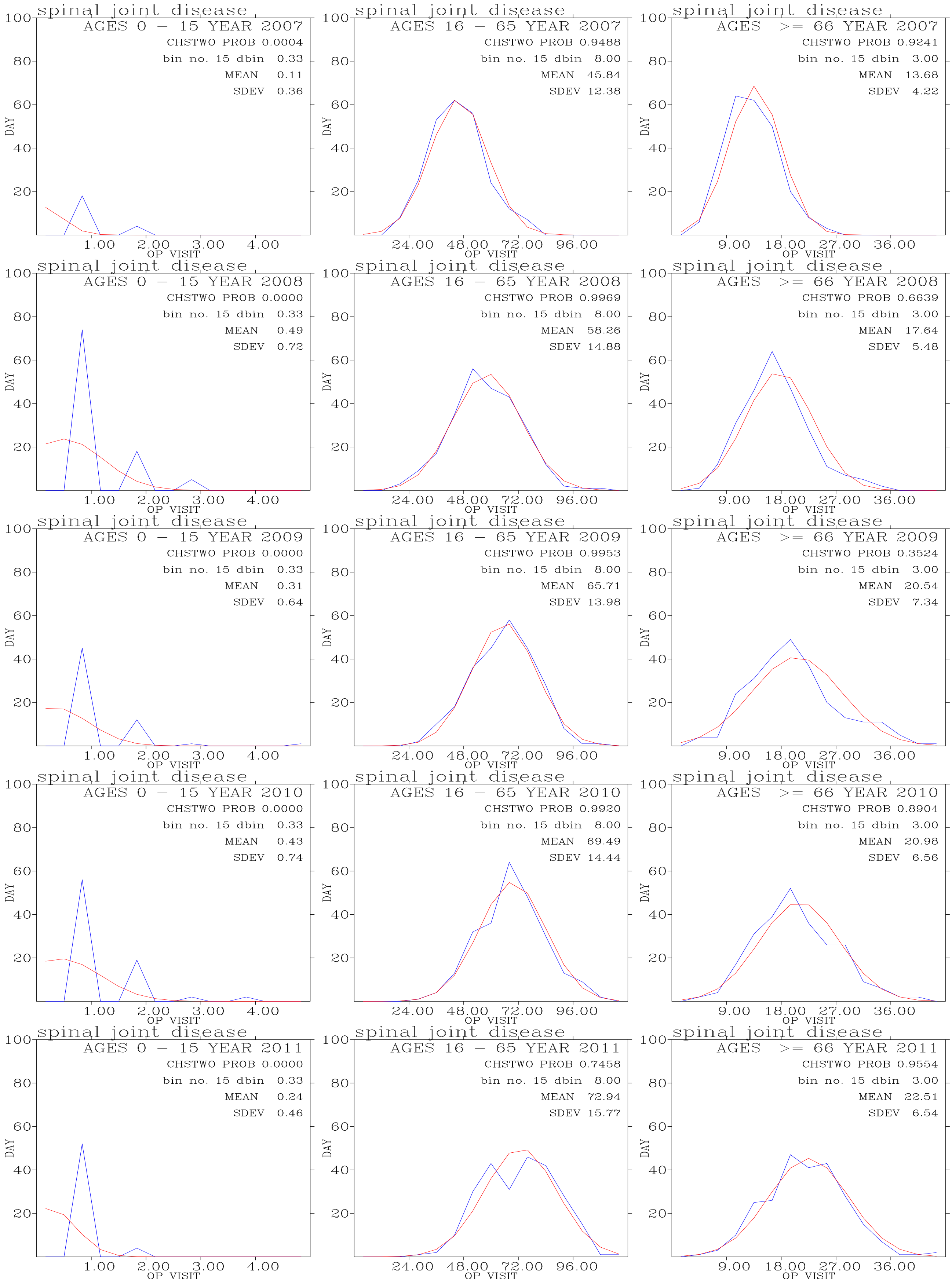

Figure 12. Test of normality distribution for spondylosis disease op.

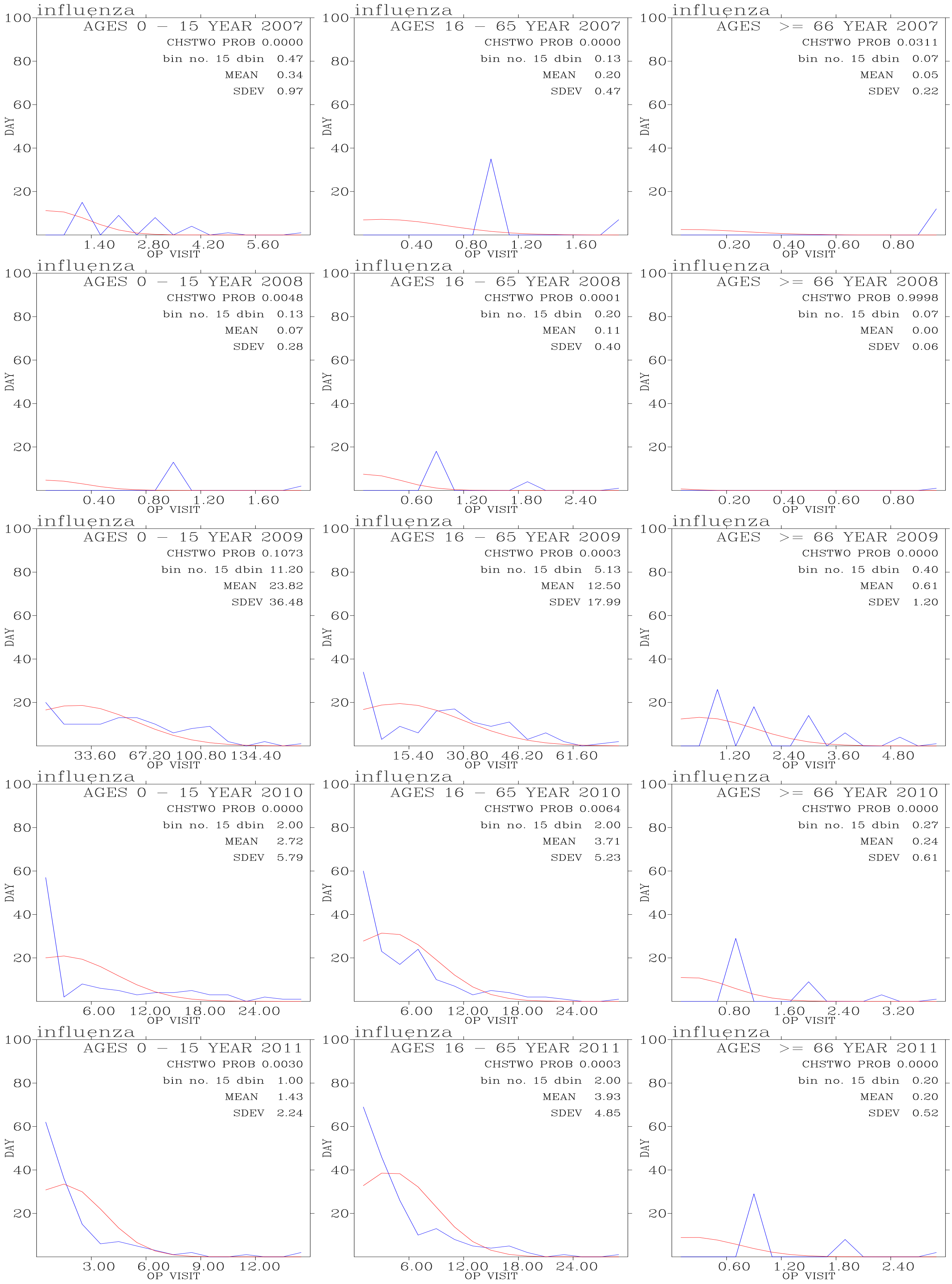

Figure 13. Test of normality distribution for influenza disease op.
