# Supplementary material for: An association between air pollution and daily most frequently visits of eighteen outpatient diseases in an industrial city
Source: Sci Rep. 2020 Feb 11;10:2321. doi: 10.1038/s41598-020-58721-0 (PMC7012860; doi:10.1038/s41598-020-58721-0)
Supplement: Supplementary file 2 — Supporting information 2 [file 41598_2020_58721_MOESM2_ESM.pdf]

An association between air pollution and daily most frequently visits of eighteen outpatient diseases in an industrial city: Supporting information S2 Appendix

Tang-Tat Chau
Department of Family Medicine, Taiwan Landseed Hospital, Ping-Jen, Taiwan

Kuo-Ying Wang
Department of Atmospheric Sciences, National Central University, Chung-Li, Taiwan

Short title: AIR POLLUTION HEALTH RISKS

Abstract. In this S2 Appendix, we present detailed distribution of association coefficients β ($P < 0.05$) per disease.

Allergic Rhinitis — Figure 1.

Asthma — Figure 2.

Pneumonia — Figure 3.

COPD — Figure 4.

Accidents — Figure 5.

Mental Disorders — Figure 6.

Peptic Ulcer — Figure 7.

Chronic Liver — Figure 8.

Cerebravascular Disease — Figure 9.

Heart Disease — Figure 10.

Hypertensive Disease — Figure 11.

Diabetes Mellitus — Figure 12.

Malignant Neoplasm — Figure 13.

Genitourinary System — Figure 14.

Other Disorders of Soft Tissue — Figure 15.

Osteoarthrosis — Figure 16.

Spondylosis — Figure 17.

Influenza — Figure 18.

Figures

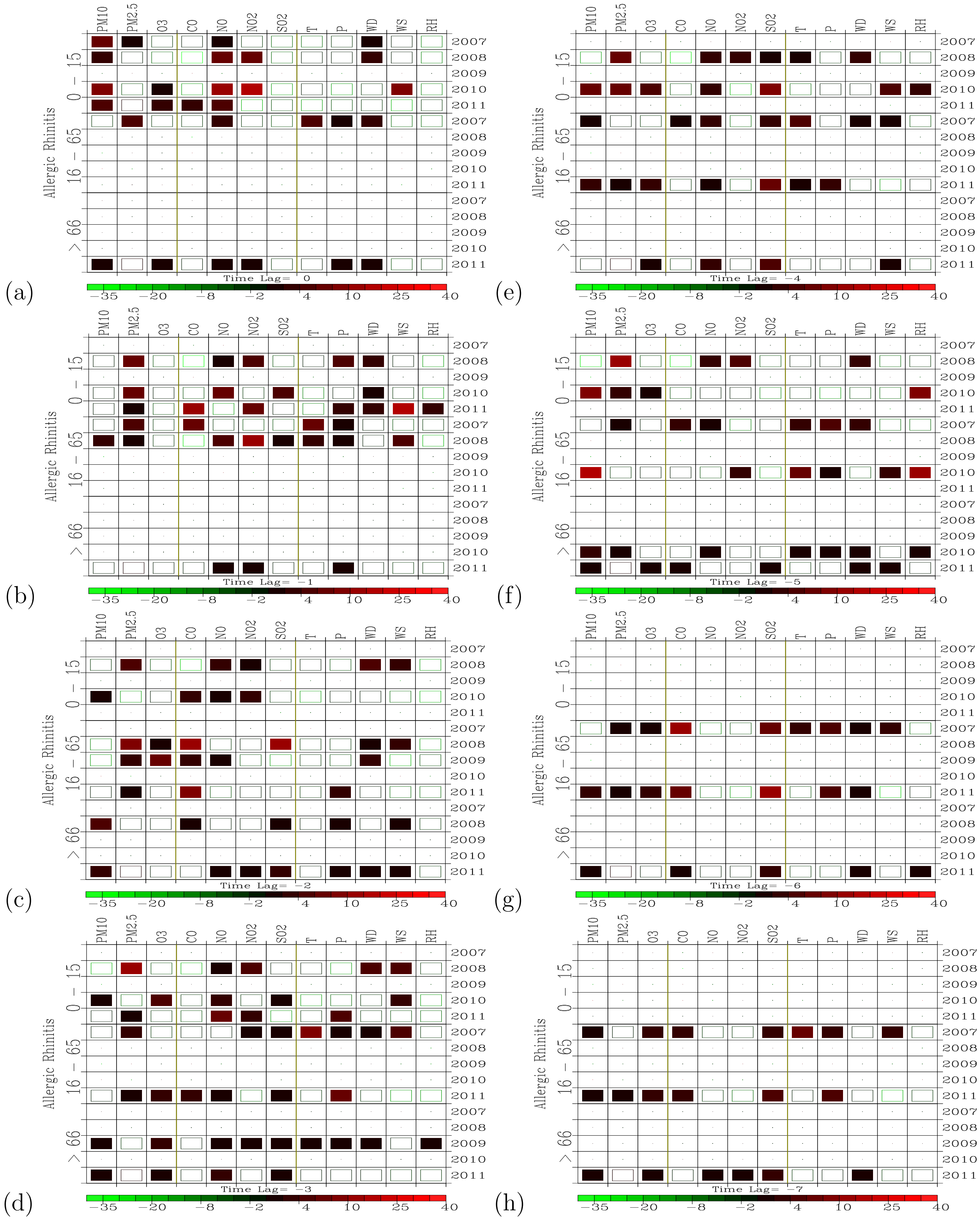

Figure 1. Distributrion of association coefficients $\beta_{i,j}$ calculated for allergic rhinitis and 3 ages group of outpatients with respect to the 12 variables: (a) 0-, (b) 1-, (c) 2-, (d) 3-, (e) 4-, (f) 5-, (g) 6-, and (h) 7-day of time lags. Positive association coefficients are shown as red colored filled squares, while negative association coefficients are shown as green colored open squares.

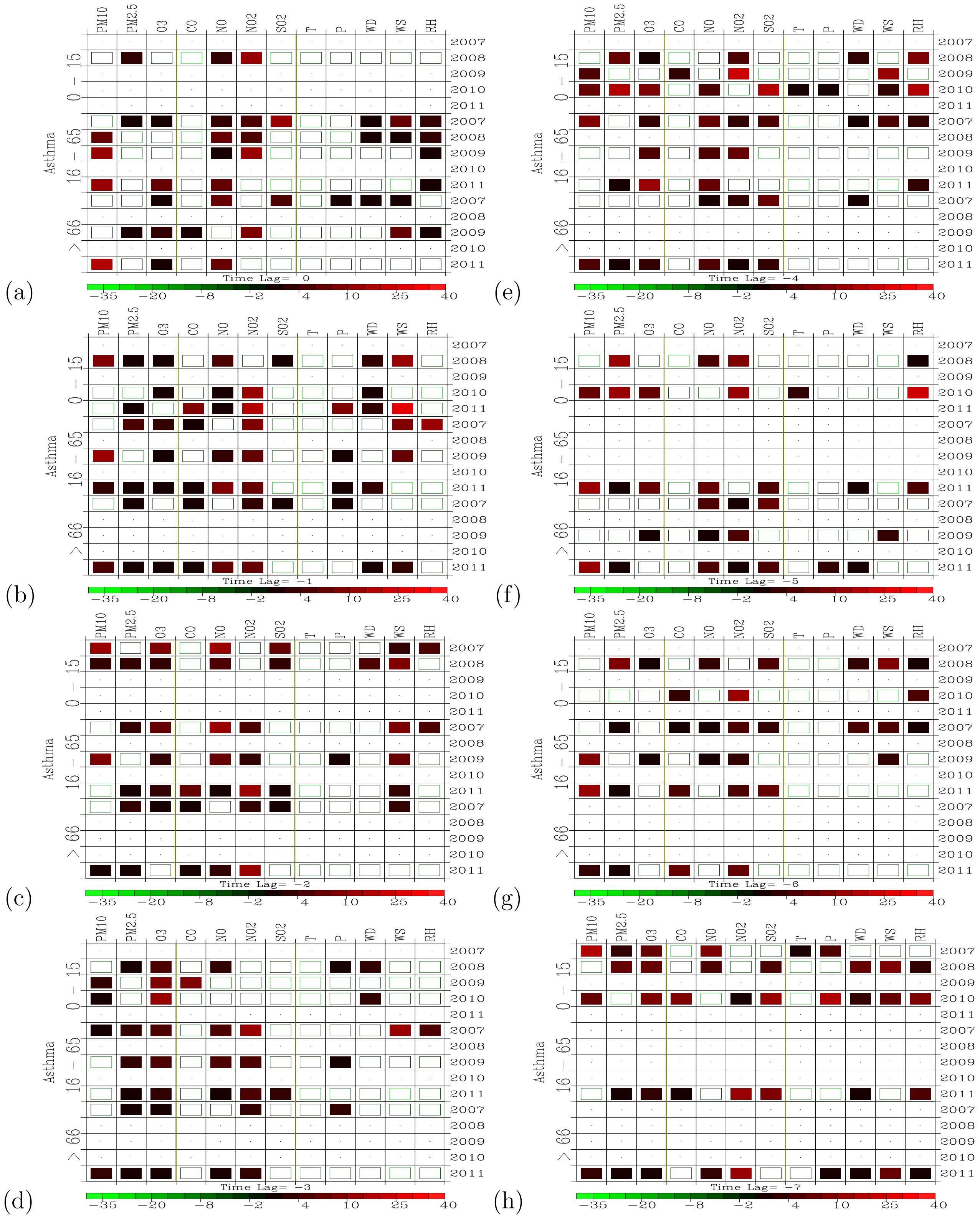

Figure 2. Distributrion of association coefficients $\beta_{i,j}$ calculated for asthma and 3 ages group of outpatients with respect to the 12 variables: (a) 0-, (b) 1-, (c) 2-, (d) 3-, (e) 4-, (f) 5-, (g) 6-, and (h) 7-day of time lags. Positive association coefficients are shown as red colored filled squares, while negative association coefficients are shown as green colored open squares.

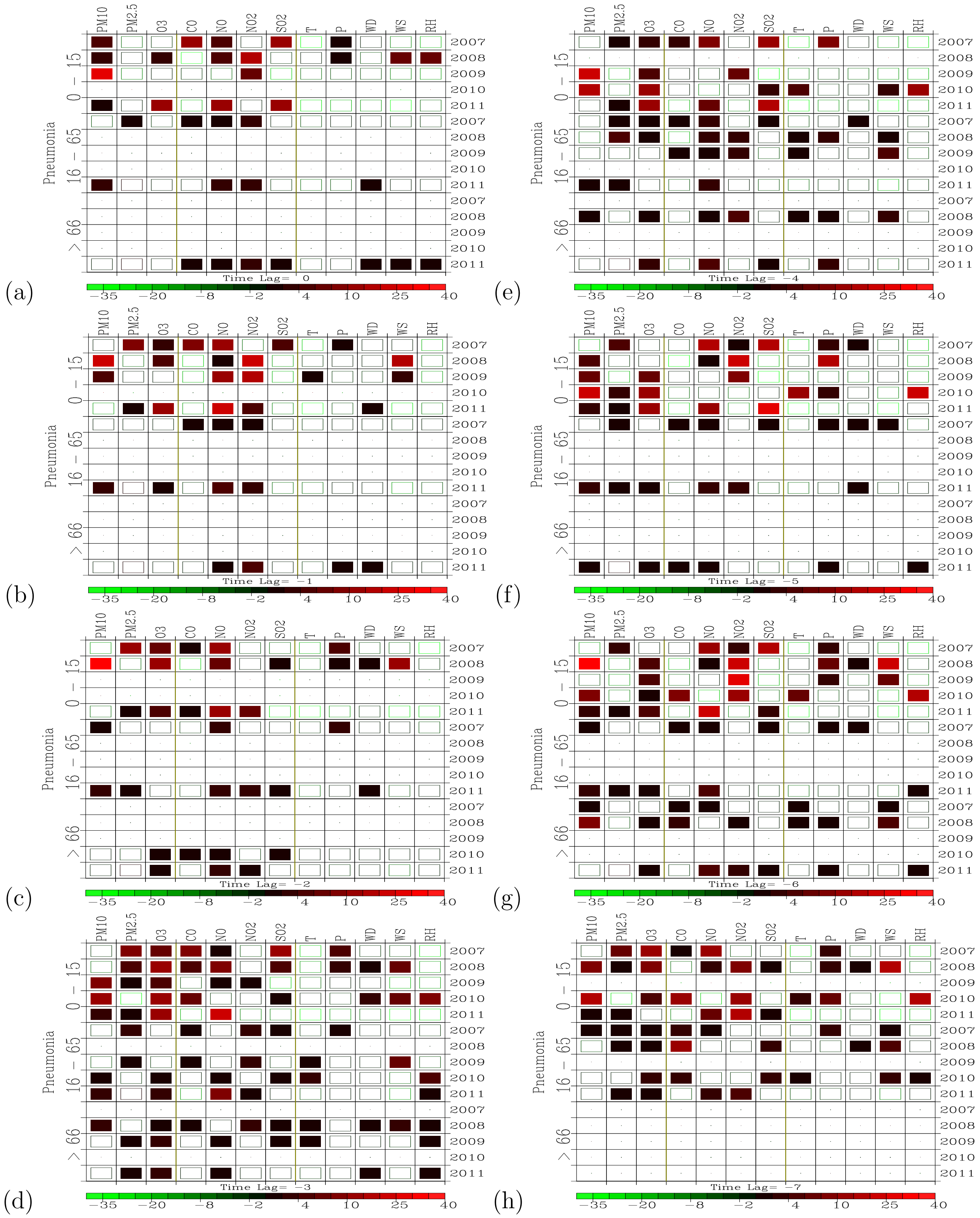

Figure 3. Distributrion of association coefficients $\beta_{i,j}$ calculated for pneumonia and 3 ages group of outpatients with respect to the 12 variables: (a) 0-, (b) 1-, (c) 2-, (d) 3-, (e) 4-, (f) 5-, (g) 6-, and (h) 7-day of time lags. Positive association coefficients are shown as red colored filled squares, while negative association coefficients are shown as green colored open squares.

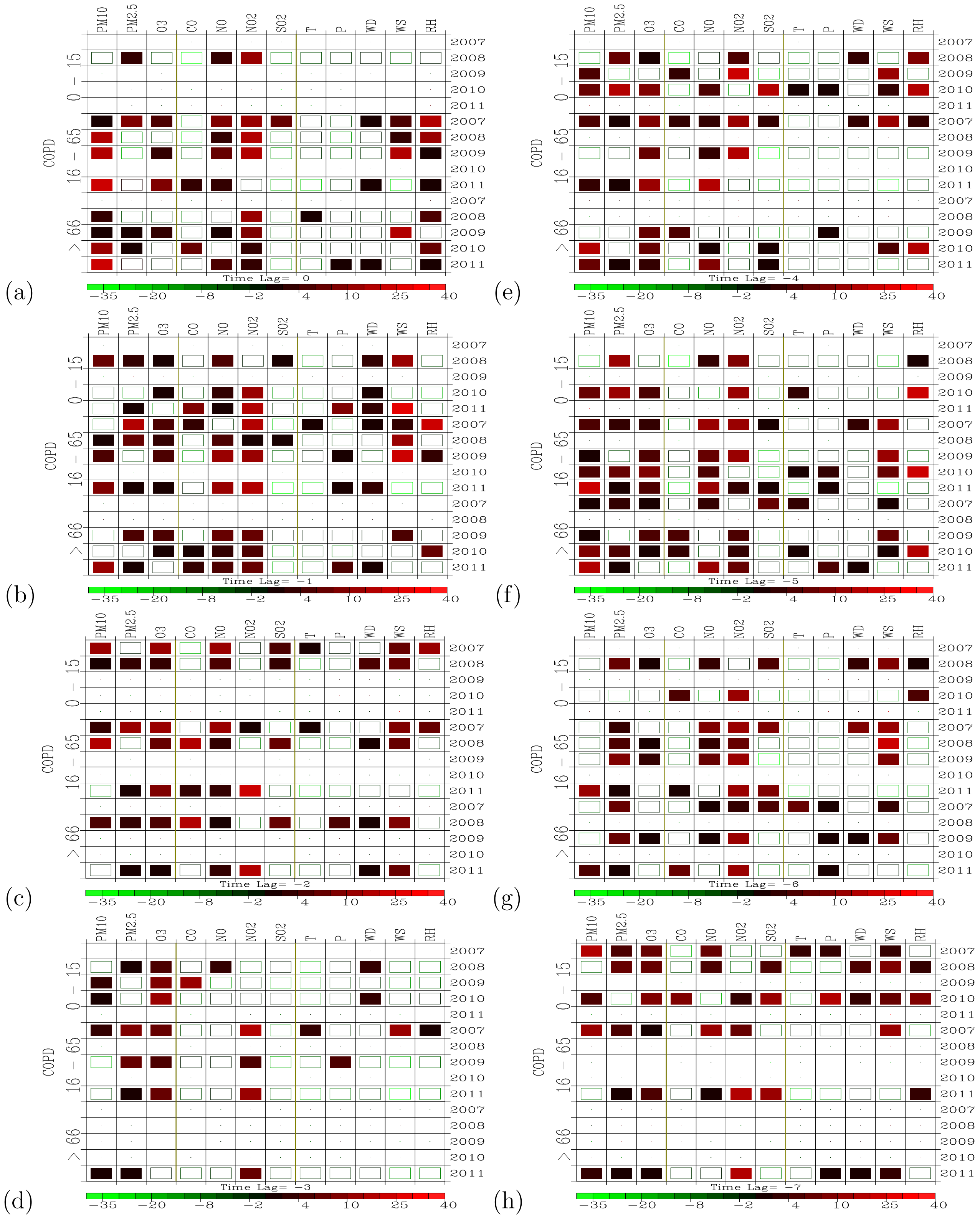

Figure 4. Distributrion of association coefficients $\beta_{i,j}$ calculated for COPD and 3 ages group of outpatients with respect to the 12 variables: (a) 0-, (b) 1-, (c) 2-, (d) 3-, (e) 4-, (f) 5-, (g) 6-, and (h) 7-day of time lags. Positive association coefficients are shown as red colored filled squares, while negative association coefficients are shown as green colored open squares.

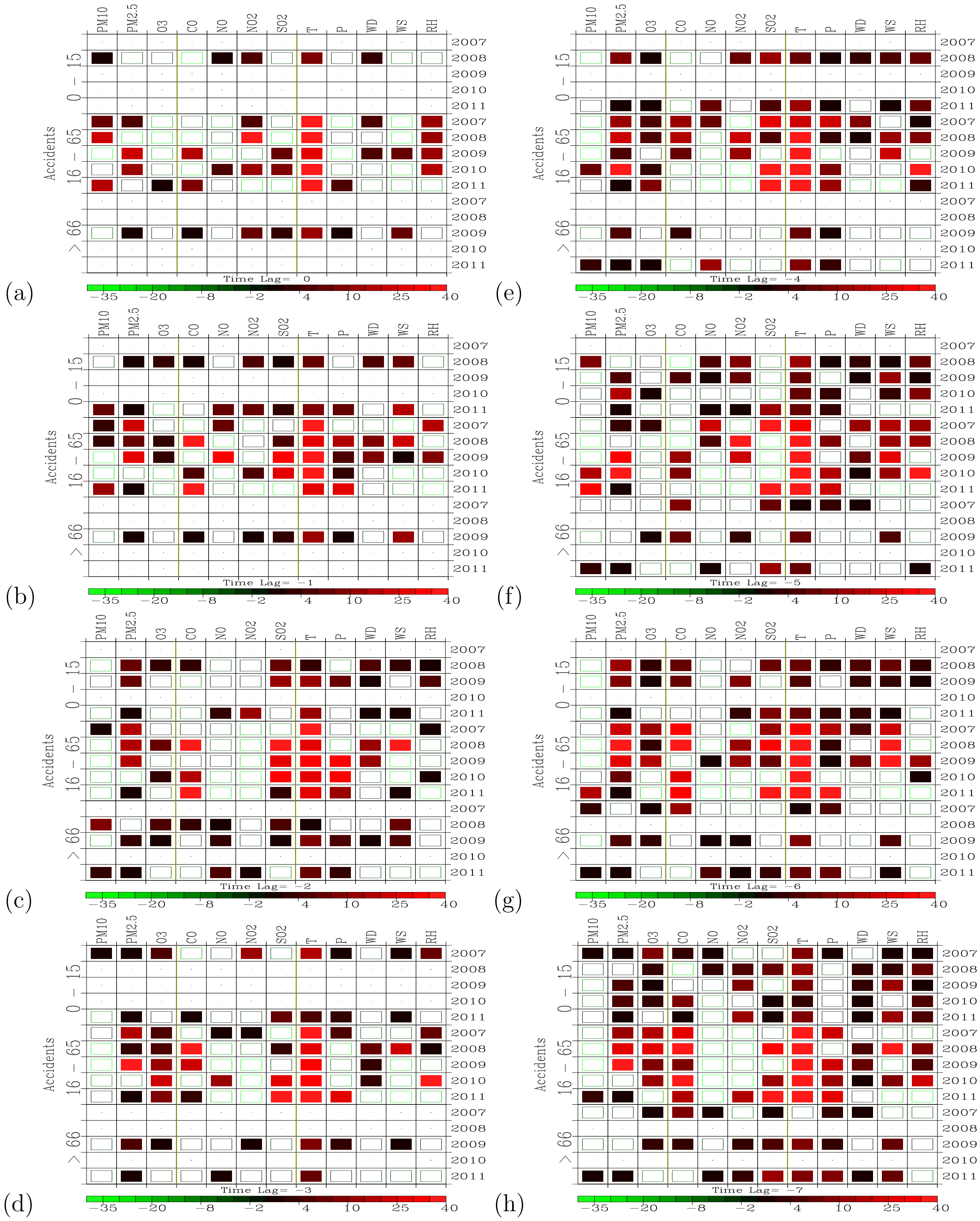

Figure 5. Distributrion of association coefficients $\beta_{\mathrm{i,j}}$ calculated for accidents and 3 ages group of outpatients with respect to the 12 variables: (a) 0-, (b) 1-, (c) 2-, (d) 3-, (e) 4-, (f) 5-, (g) 6-, and (h) 7-day of time lags. Positive association coefficients are shown as red colored filled squares, while negative association coefficients are shown as green colored open squares.

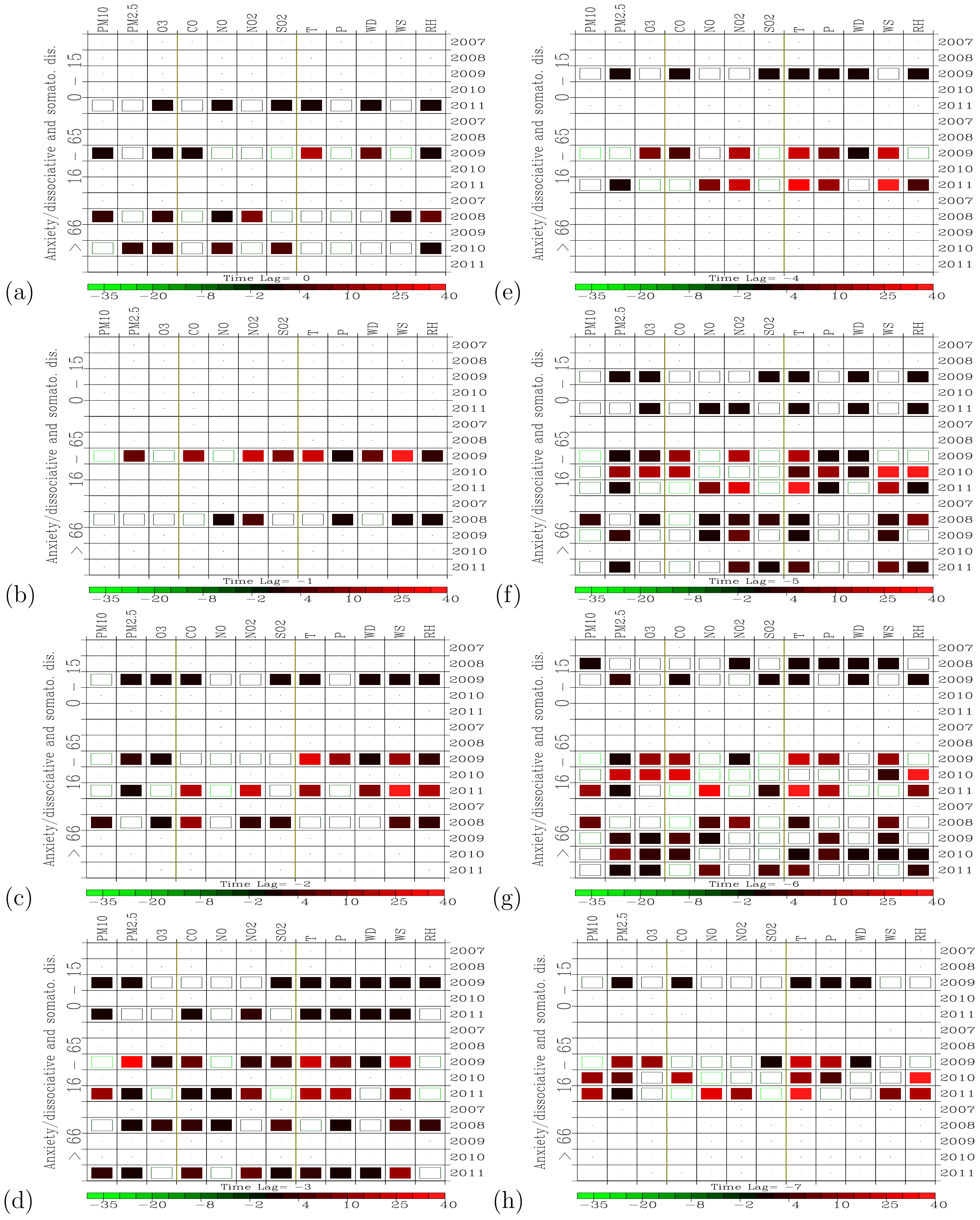

Figure 6. Distributrion of association coefficients $\beta_{i,j}$ calculated for mental disorders and 3 ages group of outpatients with respect to the 12 variables: (a) 0-, (b) 1-, (c) 2-, (d) 3-, (e) 4-, (f) 5-, (g) 6-, and (h) 7-day of time lags. Positive association coefficients are shown as red colored filled squares, while negative association coefficients are shown as green colored open squares.

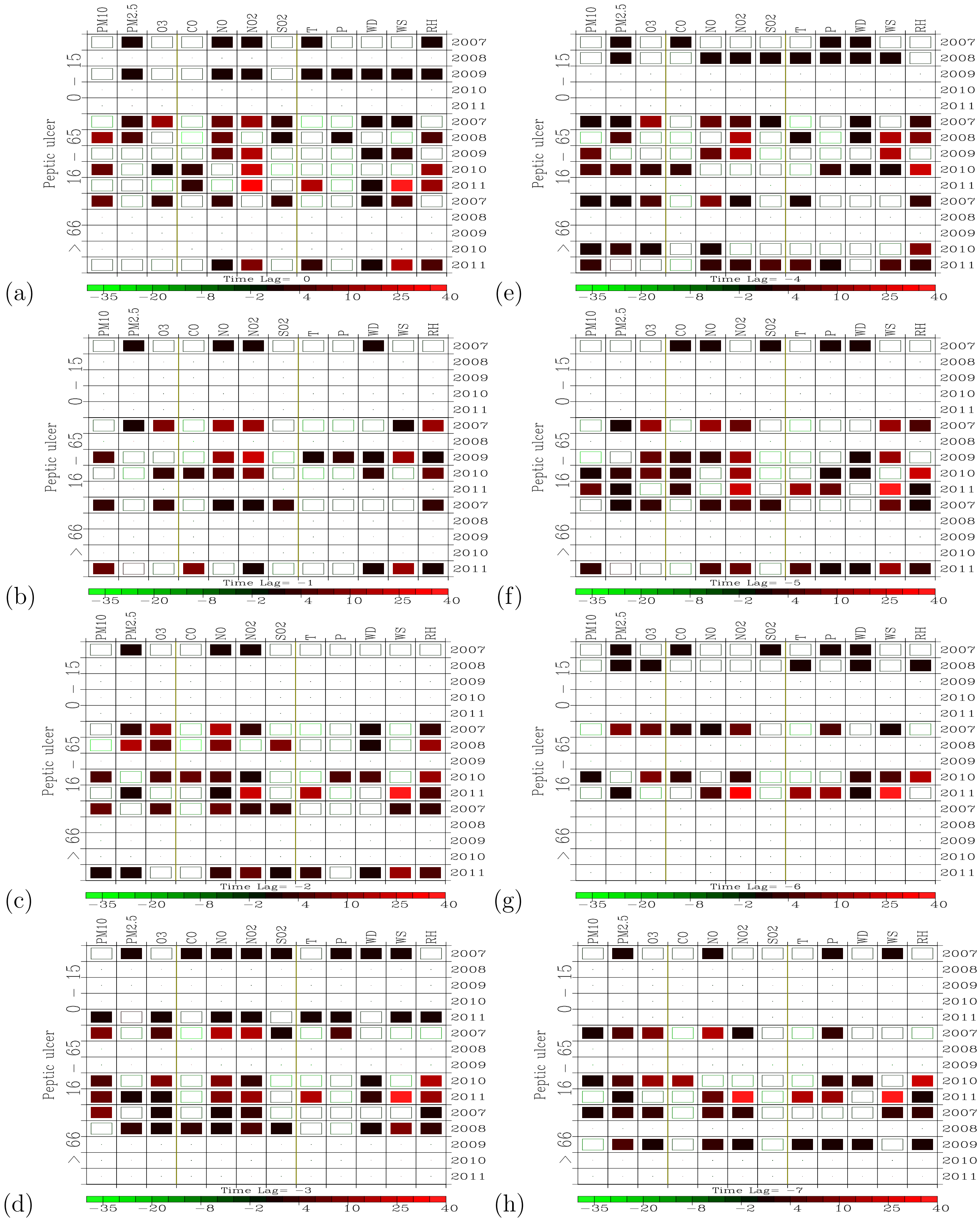

Figure 7. Distributrion of association coefficients $\beta_{i,j}$ calculated for peptic ulcer and 3 ages group of outpatients with respect to the 12 variables: (a) 0-, (b) 1-, (c) 2-, (d) 3-, (e) 4-, (f) 5-, (g) 6-, and (h) 7-day of time lags. Positive association coefficients are shown as red colored filled squares, while negative association coefficients are shown as green colored open squares.

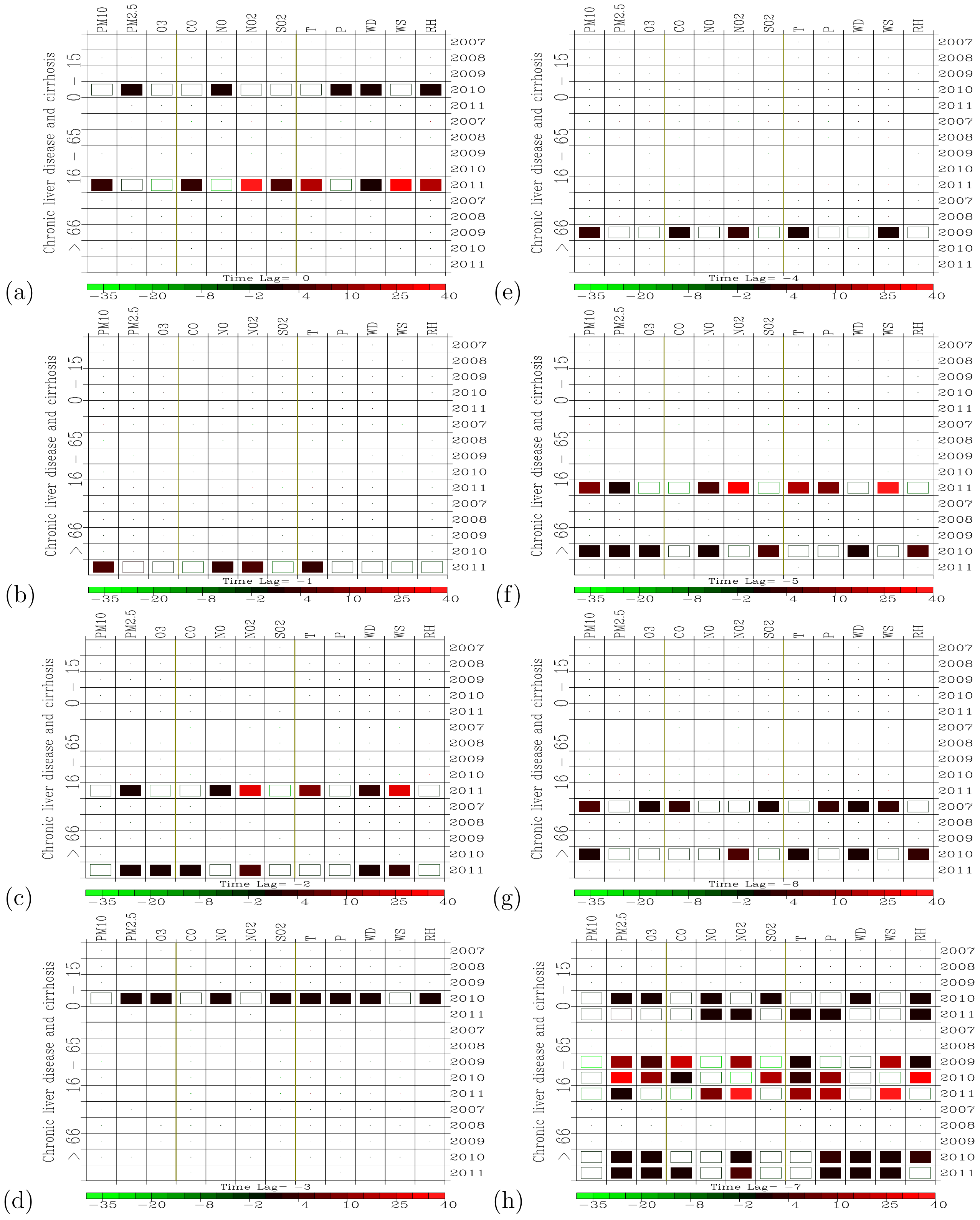

Figure 8. Distributrion of association coefficients $\beta_{i,j}$ calculated for chronic liver and 3 ages group of outpatients with respect to the 12 variables: (a) 0-, (b) 1-, (c) 2-, (d) 3-, (e) 4-, (f) 5-, (g) 6-, and (h) 7-day of time lags. Positive association coefficients are shown as red colored filled squares, while negative association coefficients are shown as green colored open squares.

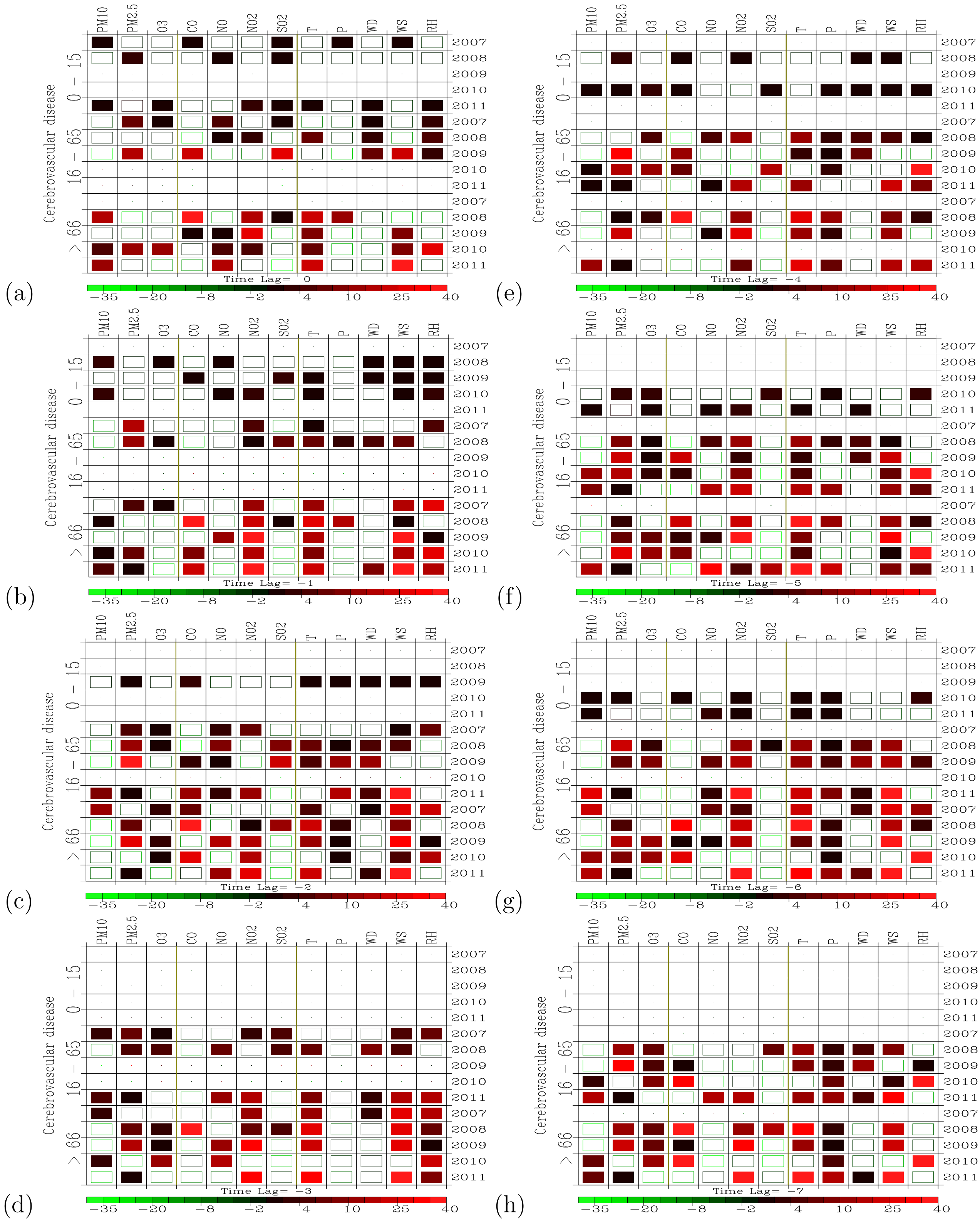

Figure 9. Distributrion of association coefficients $\beta_{i,j}$ calculated for cerebravascular disease and 3 ages group of outpatients with respect to the 12 variables: (a) 0-, (b) 1-, (c) 2-, (d) 3-, (e) 4-, (f) 5-, (g) 6-, and (h) 7-day of time lags. Positive association coefficients are shown as red colored filled squares, while negative association coefficients are shown as green colored open squares.

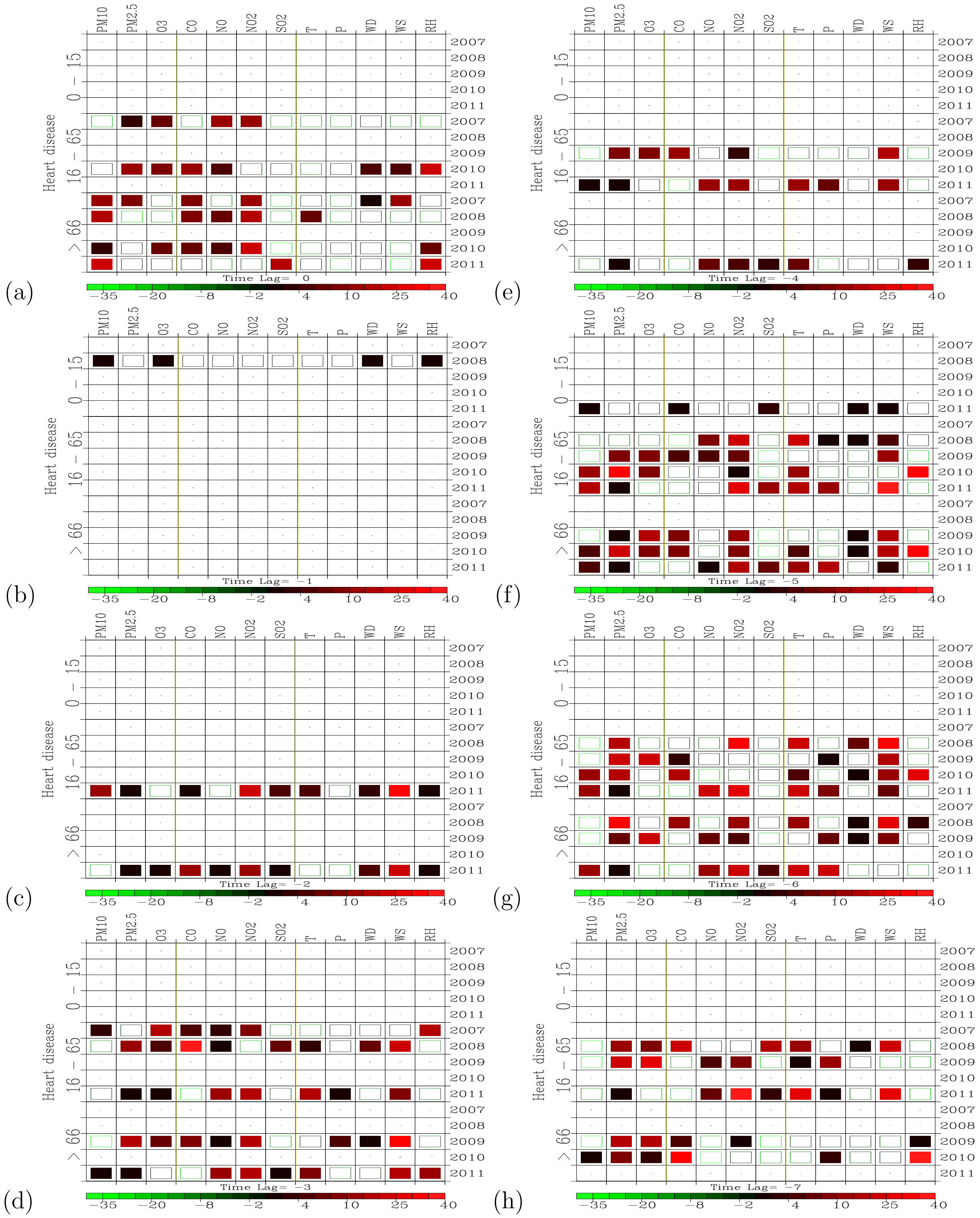

Figure 10. Distributrion of association coefficients $\beta_{i,j}$ calculated for heart disease and 3 ages group of outpatients with respect to the 12 variables: (a) 0-, (b) 1-, (c) 2-, (d) 3-, (e) 4-, (f) 5-, (g) 6-, and (h) 7-day of time lags. Positive association coefficients are shown as red colored filled squares, while negative association coefficients are shown as green colored open squares.

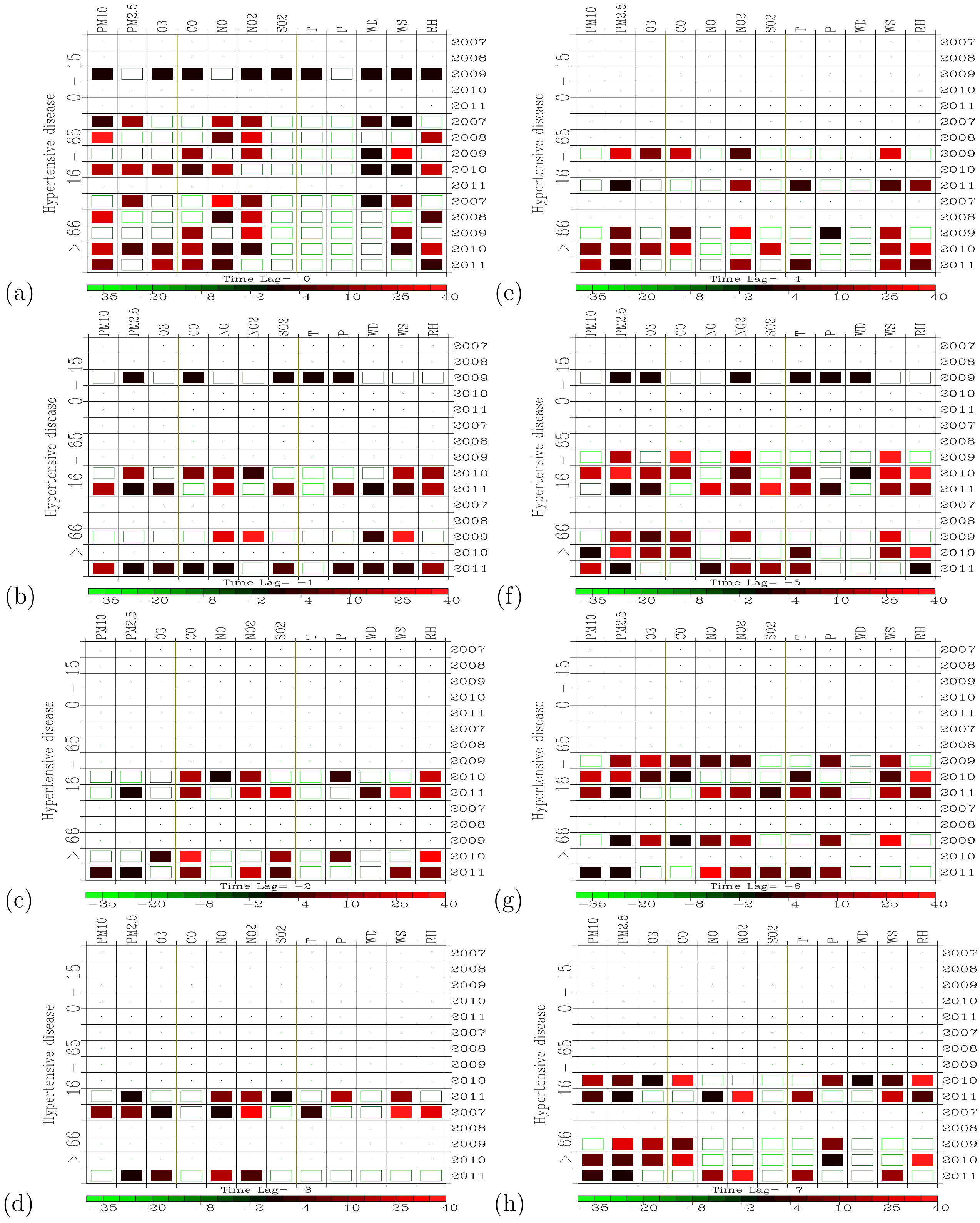

Figure 11. Distributrion of association coefficients $\beta_{i,j}$ calculated for hypertensive disease and 3 ages group of outpatients with respect to the 12 variables: (a) 0-, (b) 1-, (c) 2-, (d) 3-, (e) 4-, (f) 5-, (g) 6-, and (h) 7-day of time lags. Positive association coefficients are shown as red colored filled squares, while negative association coefficients are shown as green colored open squares.

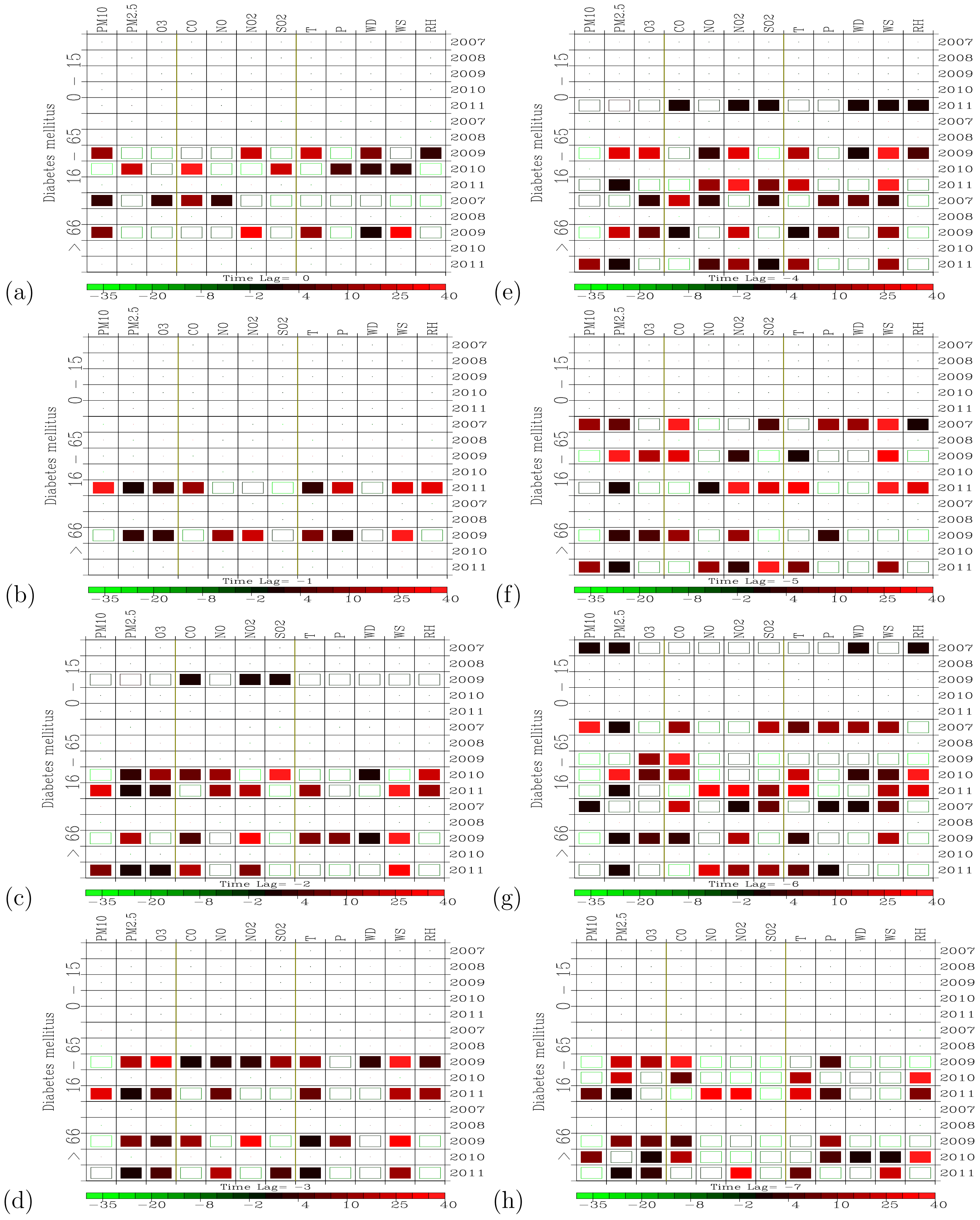

Figure 12. Distributrion of association coefficients $\beta_{i,j}$ calculated for diabetes mellitus and 3 ages group of outpatients with respect to the 12 variables: (a) 0-, (b) 1-, (c) 2-, (d) 3-, (e) 4-, (f) 5-, (g) 6-, and (h) 7-day of time lags. Positive association coefficients are shown as red colored filled squares, while negative association coefficients are shown as green colored open squares.

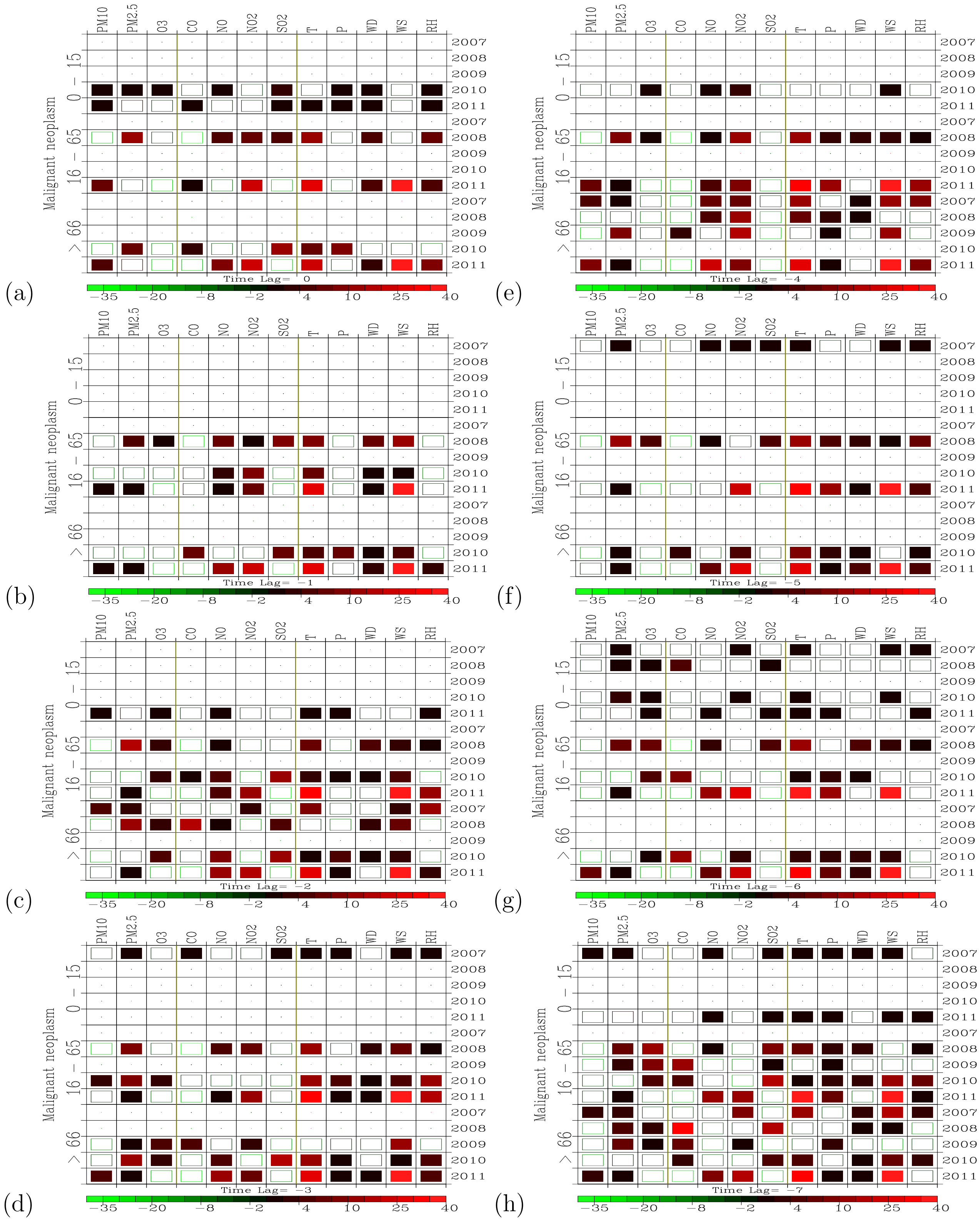

Figure 13. Distributron of association coefficients $\beta_{i,j}$ calculated for malignant neoplasm and 3 ages group of outpatients with respect to the 12 variables: (a) 0-, (b) 1-, (c) 2-, (d) 3-, (e) 4-, (f) 5-, (g) 6-, and (h) 7-day of time lags. Positive association coefficients are shown as red colored filled squares, while negative association coefficients are shown as green colored open squares.

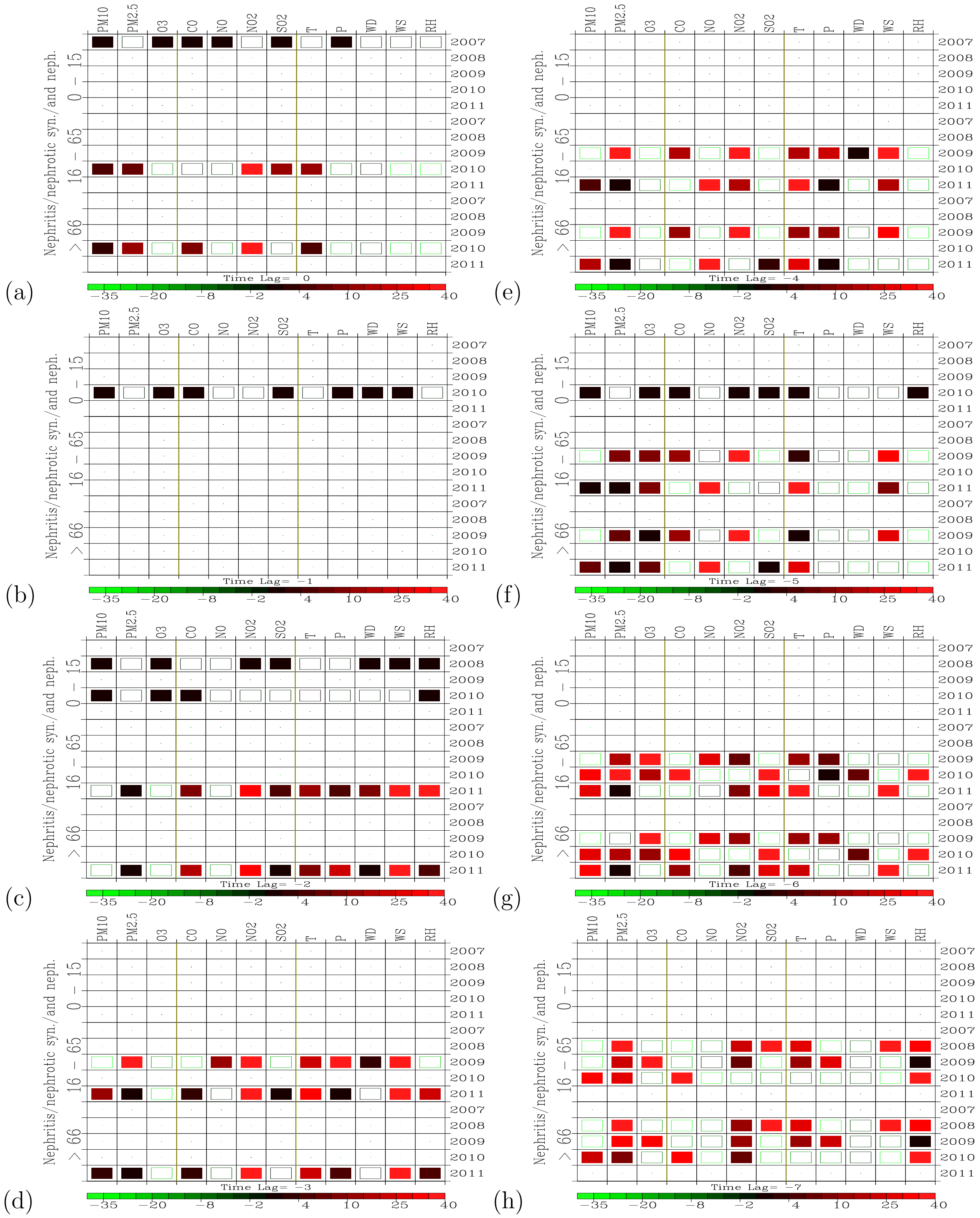

Figure 14. Distributrion of association coefficients $\beta_{i,j}$ calculated for genitourinary system and 3 ages group of outpatients with respect to the 12 variables: (a) 0-, (b) 1-, (c) 2-, (d) 3-, (e) 4-, (f) 5-, (g) 6-, and (h) 7-day of time lags. Positive association coefficients are shown as red colored filled squares, while negative association coefficients are shown as green colored open squares.

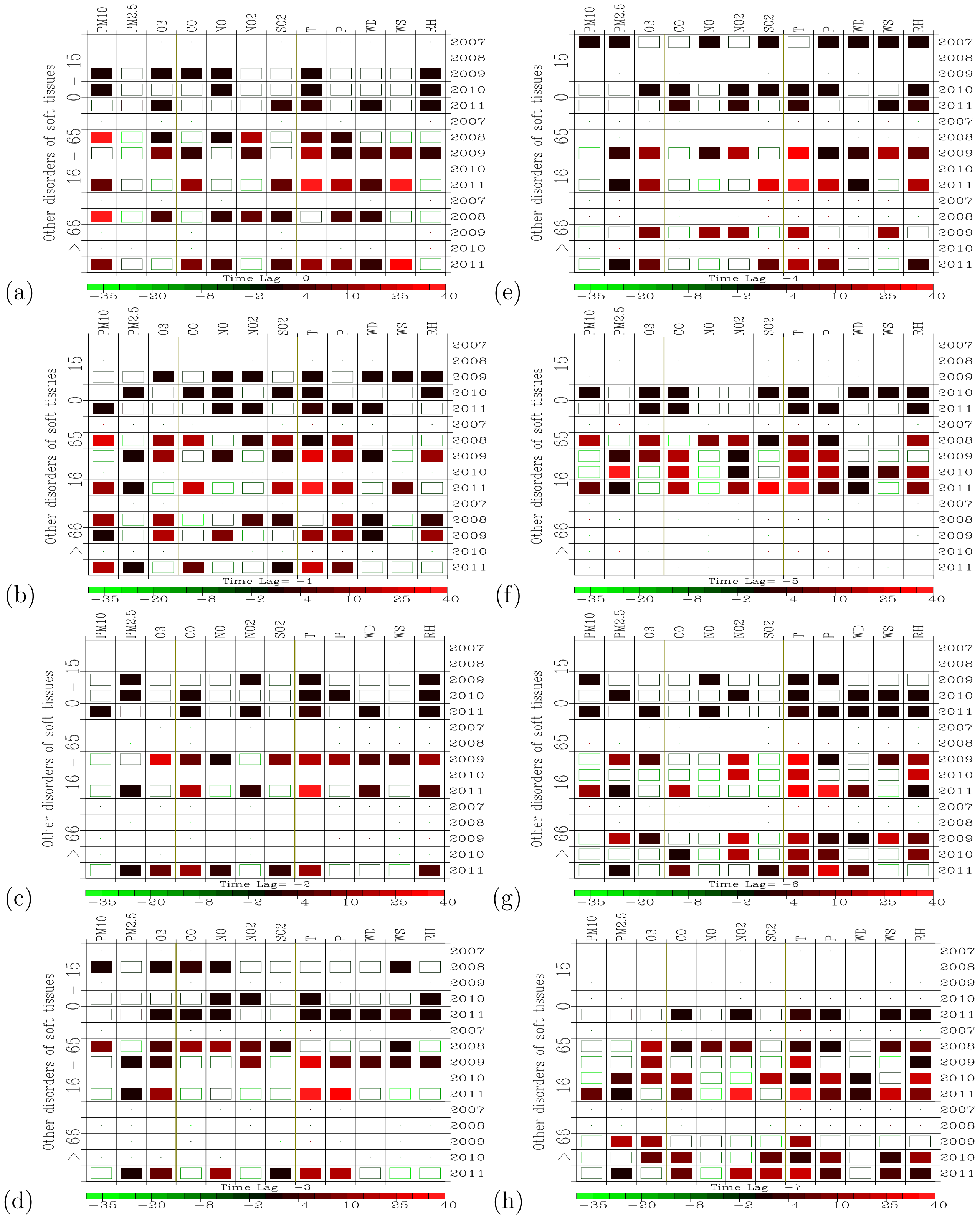

Figure 15. Distributrion of association coefficients $\beta_{i,j}$ calculated for other disorders of soft tissue and 3 ages group of outpatients with respect to the 12 variables: (a) 0-, (b) 1-, (c) 2-, (d) 3-, (e) 4-, (f) 5-, (g) 6-, and (h) 7-day of time lags. Positive association coefficients are shown as red colored filled squares, while negative association coefficients are shown as green colored open squares.

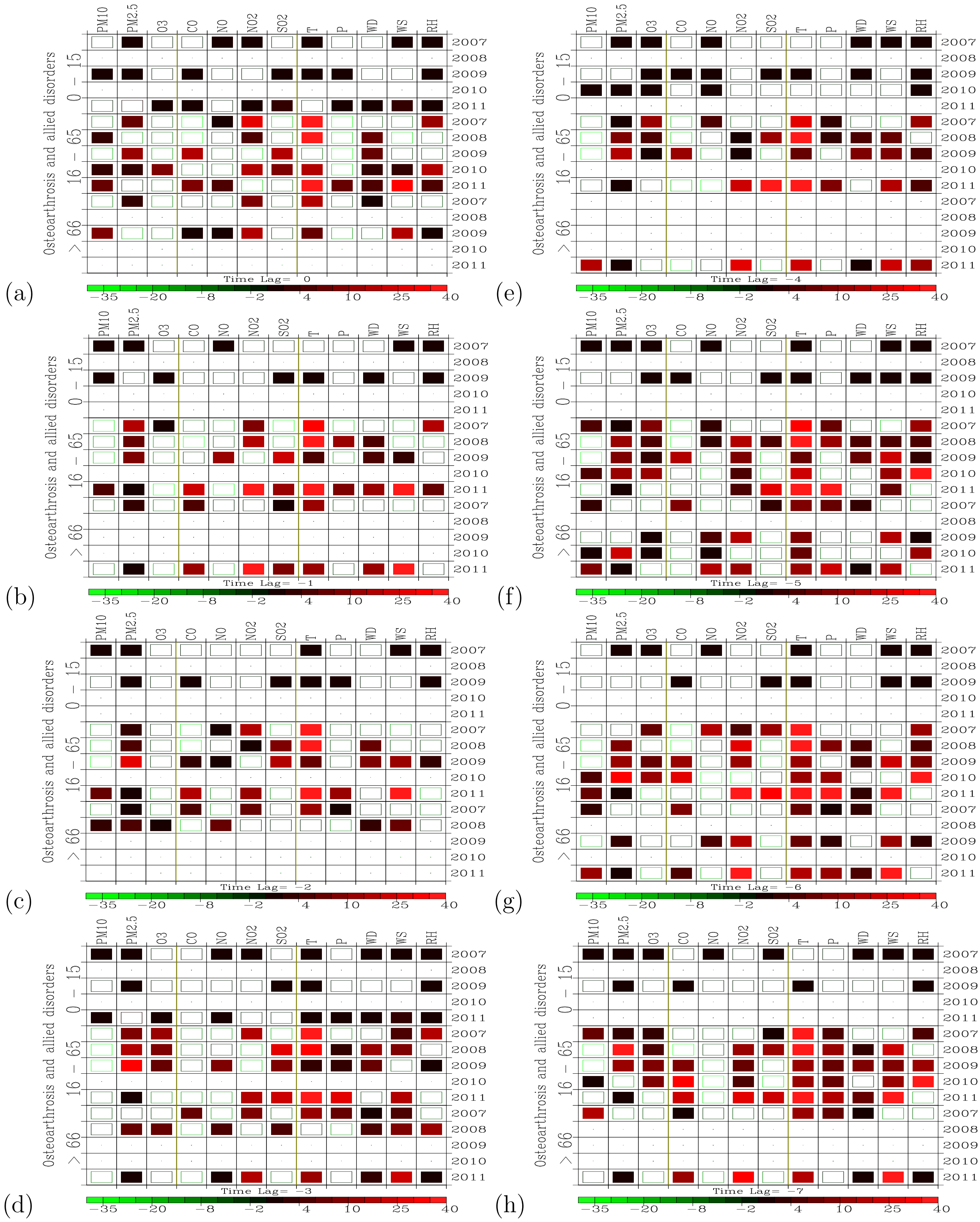

Figure 16. Distributrion of association coefficients $\beta_{i,j}$ calculated for osteoarthrosis and 3 ages group of outpatients with respect to the 12 variables: (a) 0-, (b) 1-, (c) 2-, (d) 3-, (e) 4-, (f) 5-, (g) 6-, and (h) 7-day of time lags. Positive association coefficients are shown as red colored filled squares, while negative association coefficients are shown as green colored open squares.

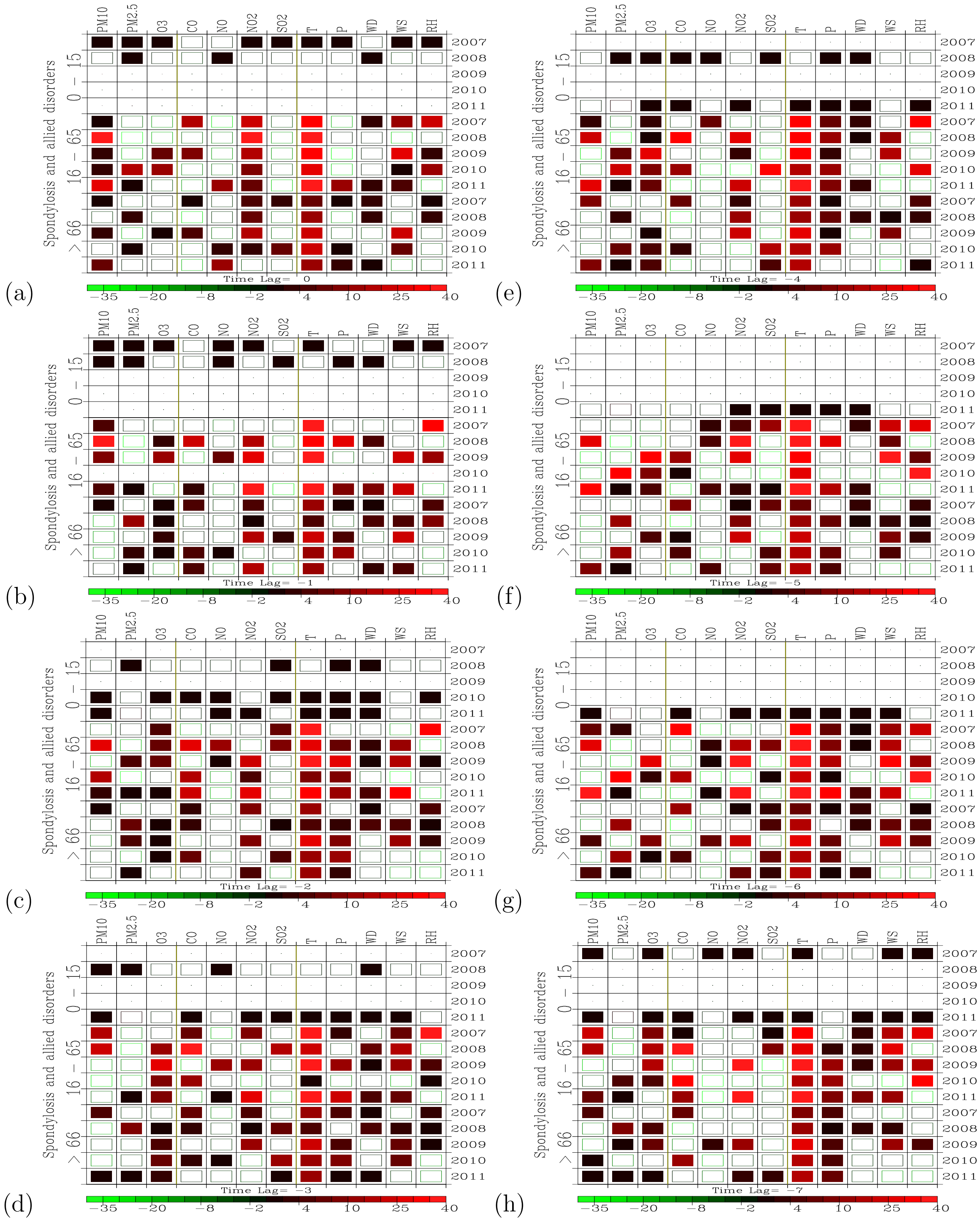

Figure 17. Distributrion of association coefficients $\beta_{i,j}$ calculated for spondylosis and 3 ages group of outpatients with respect to the 12 variables: (a) 0-, (b) 1-, (c) 2-, (d) 3-, (e) 4-, (f) 5-, (g) 6-, and (h) 7-day of time lags. Positive association coefficients are shown as red colored filled squares, while negative association coefficients are shown as green colored open squares.

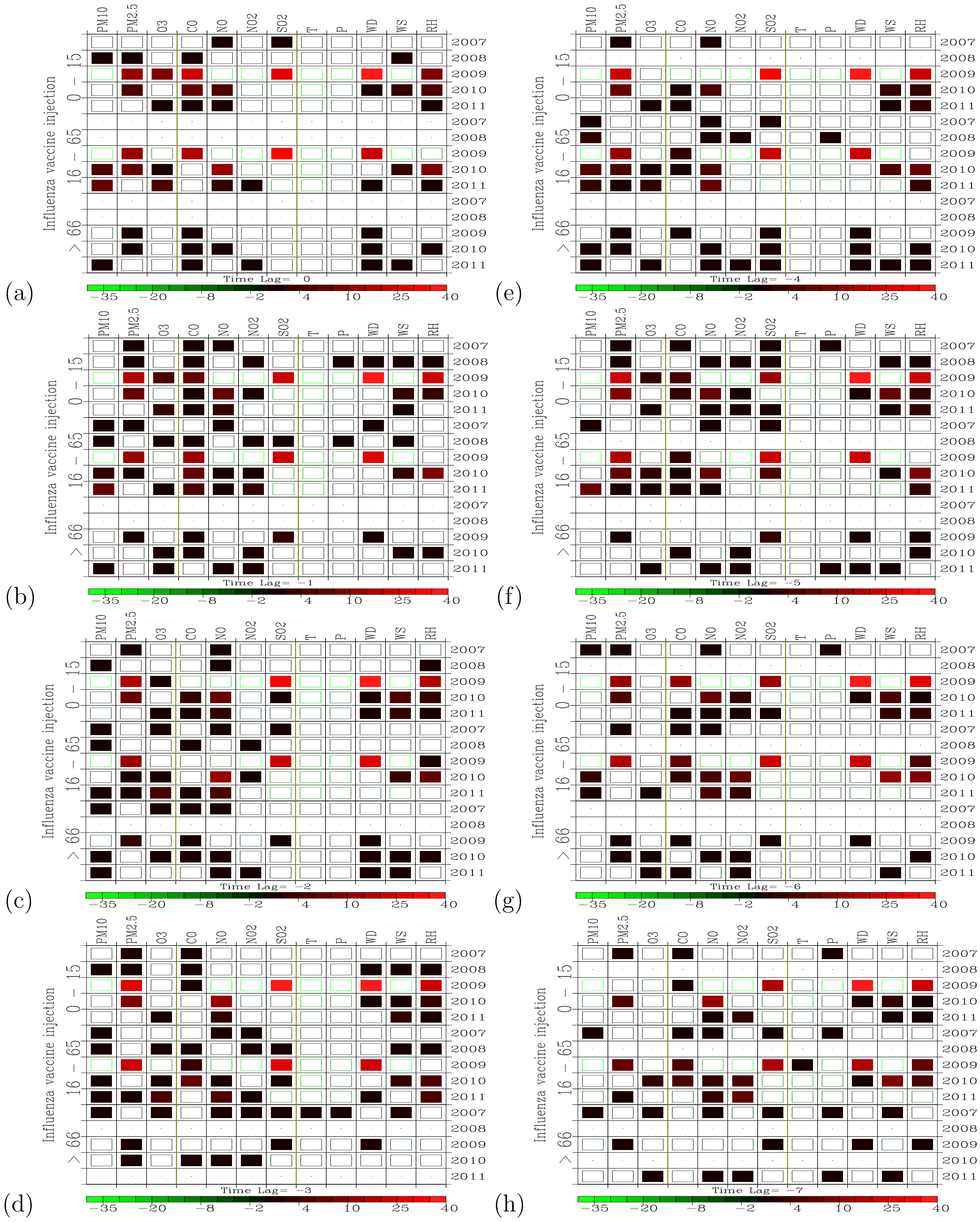

Figure 18. Distributrion of association coefficients $\beta_{i,j}$ calculated for influenza and 3 ages group of outpatients with respect to the 12 variables: (a) 0-, (b) 1-, (c) 2-, (d) 3-, (e) 4-, (f) 5-, (g) 6-, and (h) 7-day of time lags. Positive association coefficients are shown as red colored filled squares, while negative association coefficients are shown as green colored open squares.
