# Supplementary material for: An association between air pollution and daily most frequently visits of eighteen outpatient diseases in an industrial city
Source: Sci Rep. 2020 Feb 11;10:2321. doi: 10.1038/s41598-020-58721-0 (PMC7012860; doi:10.1038/s41598-020-58721-0)
Supplement: Supplementary file 3 — Supporting information 3 [file 41598_2020_58721_MOESM3_ESM.pdf]

An association between air pollution and daily most frequently visits of eighteen outpatient diseases in an industrial city: Supporting information S3 Appendix

Tang-Tat Chau
Department of Family Medicine, Taiwan Landseed Hospital, Ping-Jen, Taiwan

Kuo-Ying Wang
Department of Atmospheric Sciences, National Central University, Chung-Li, Taiwan

Short title: AIR POLLUTION HEALTH RISKS

Abstract. In this S3 Appendix, we present detailed distribution of association coefficients β ($P < 0.05$) per air pollutant.

PM10 **Figure 1.**

PM2.5 **Figure 2.**

O3 **Figure 3.**

CO **Figure 4.**

NO **Figure 5.**

NO2 **Figure 6.**

SO2 **Figure 7.**

T **Figure 8.**

P **Figure 9.**

WD **Figure 10.**

WS **Figure 11.**

RH **Figure 12.**

Figures

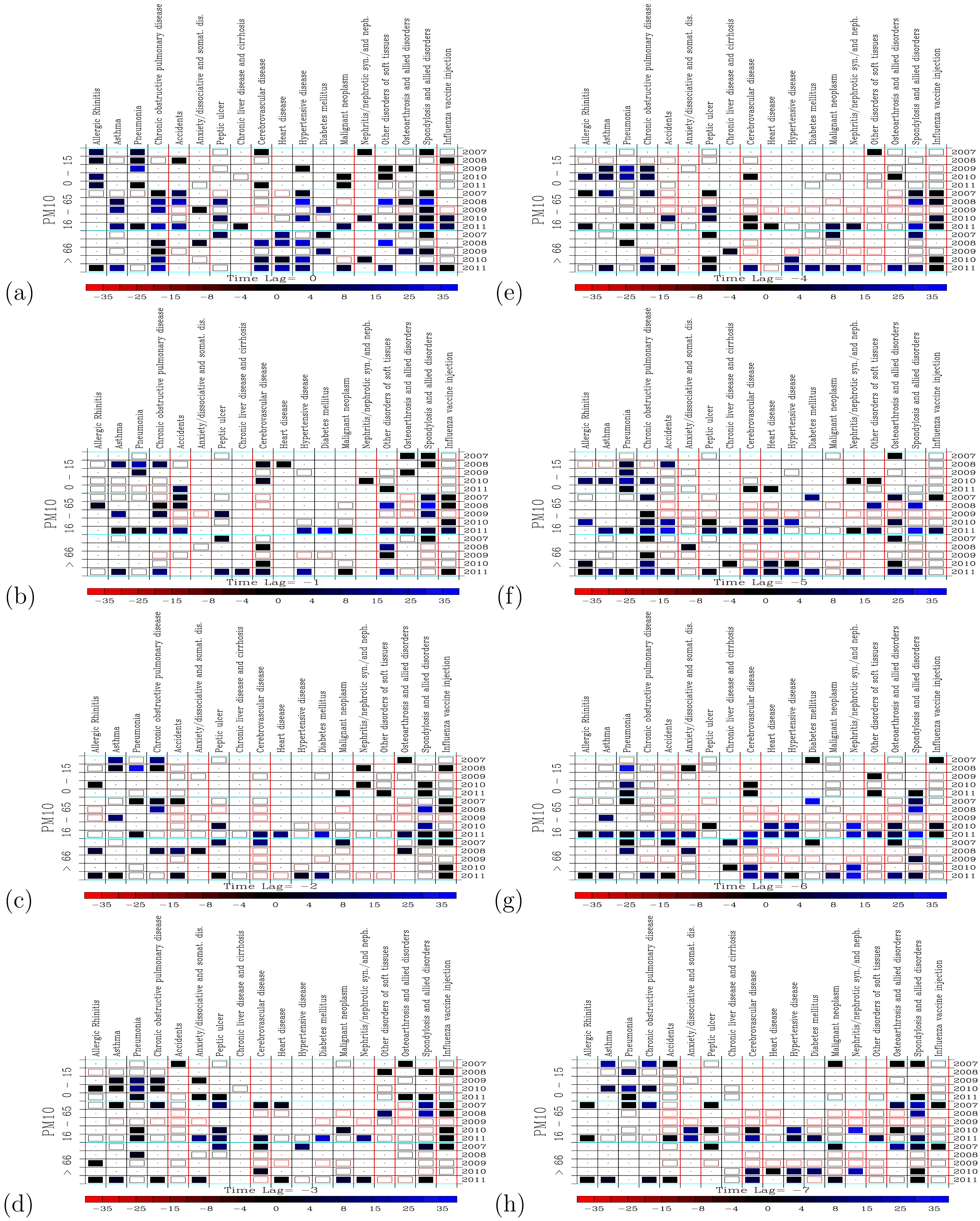

Figure 1. Distributrion of association coefficients $\beta_{\mathrm{i,j}}$ calculated for PM10 and 3 ages group of outpatients with respect to the 12 variables: (a) 0-, (b) 1-, (c) 2-, (d) 3-, (e) 4-, (f) 5-, (g) 6-, and (h) 7-day of time lags. Positive association coefficients are shown as blue colored filled squares, while negative association coefficients are shown as red colored open squares.

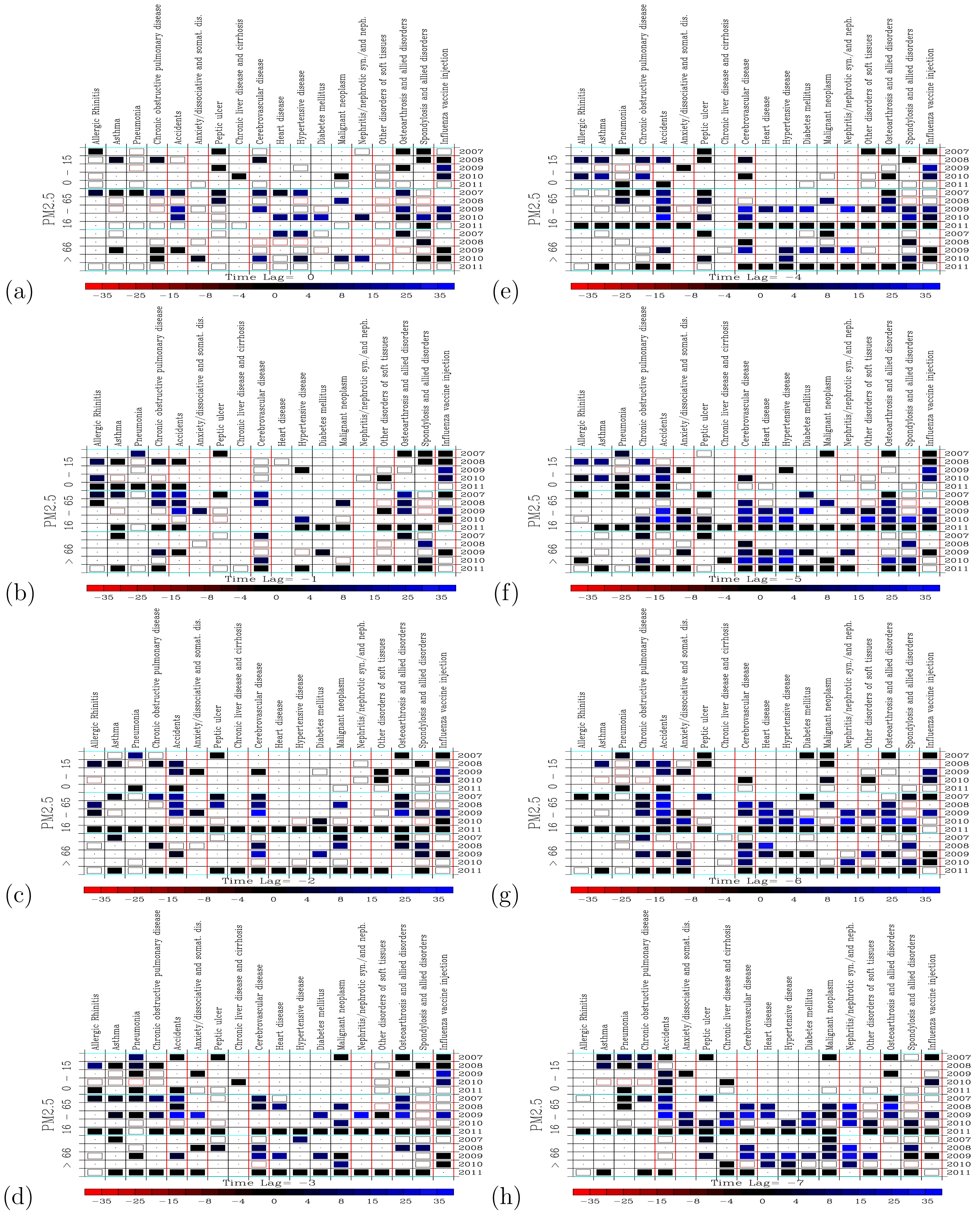

Figure 2. Distributrion of association coefficients $\beta_{\mathrm{i,j}}$ calculated for PM2.5 and 3 ages group of outpatients with respect to the 12 variables: (a) 0-, (b) 1-, (c) 2-, (d) 3-, (e) 4-, (f) 5-, (g) 6-, and (h) 7-day of time lags. Positive association coefficients are shown as blue colored filled squares, while negative association coefficients are shown as red colored open squares.

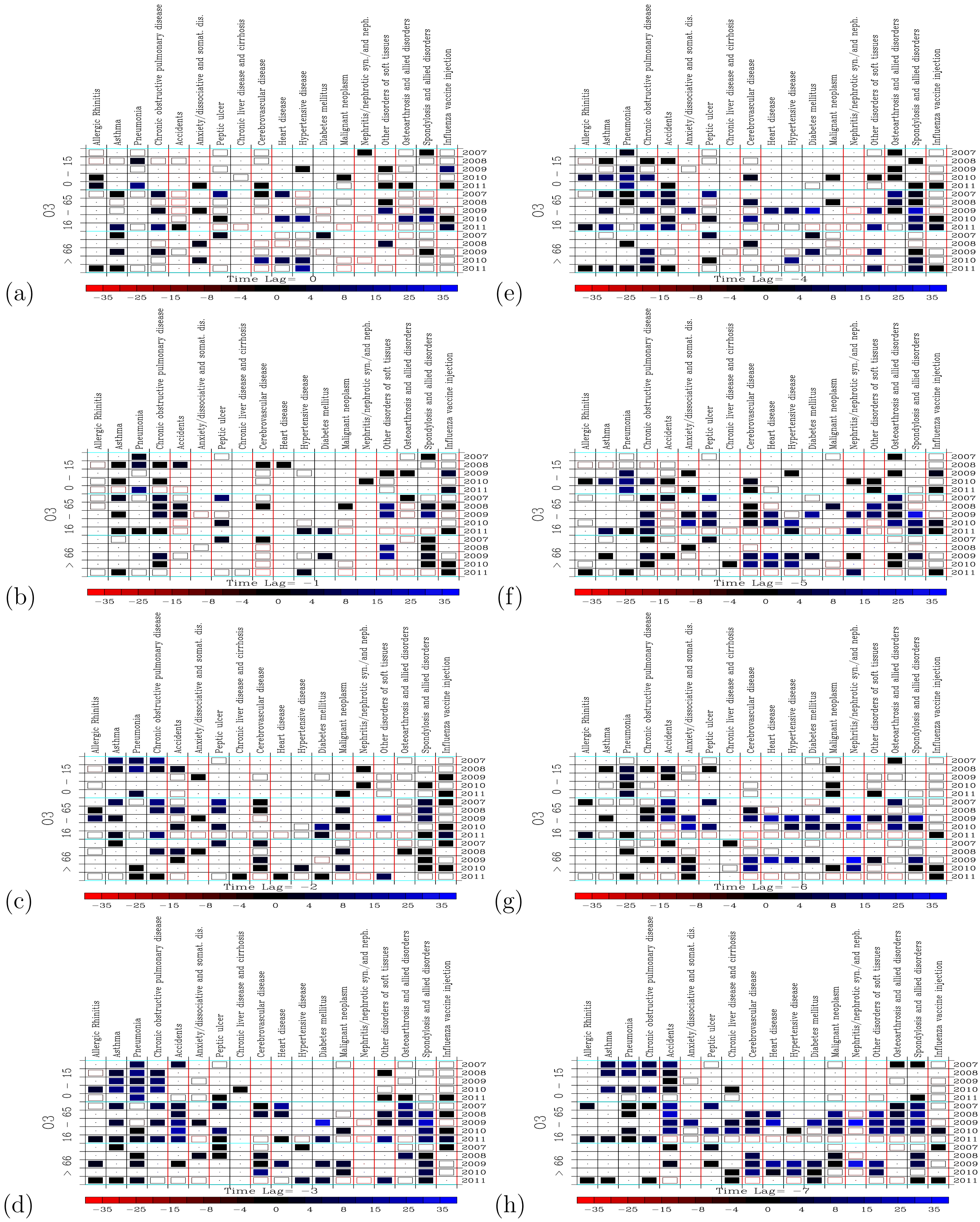

Figure 3. Distributrion of association coefficients $\beta_{i,j}$ calculated for O3 and 3 ages group of outpatients with respect to the 12 variables: (a) 0-, (b) 1-, (c) 2-, (d) 3-, (e) 4-, (f) 5-, (g) 6-, and (h) 7-day of time lags. Positive association coefficients are shown as blue colored filled squares, while negative association coefficients are shown as red colored open squares.

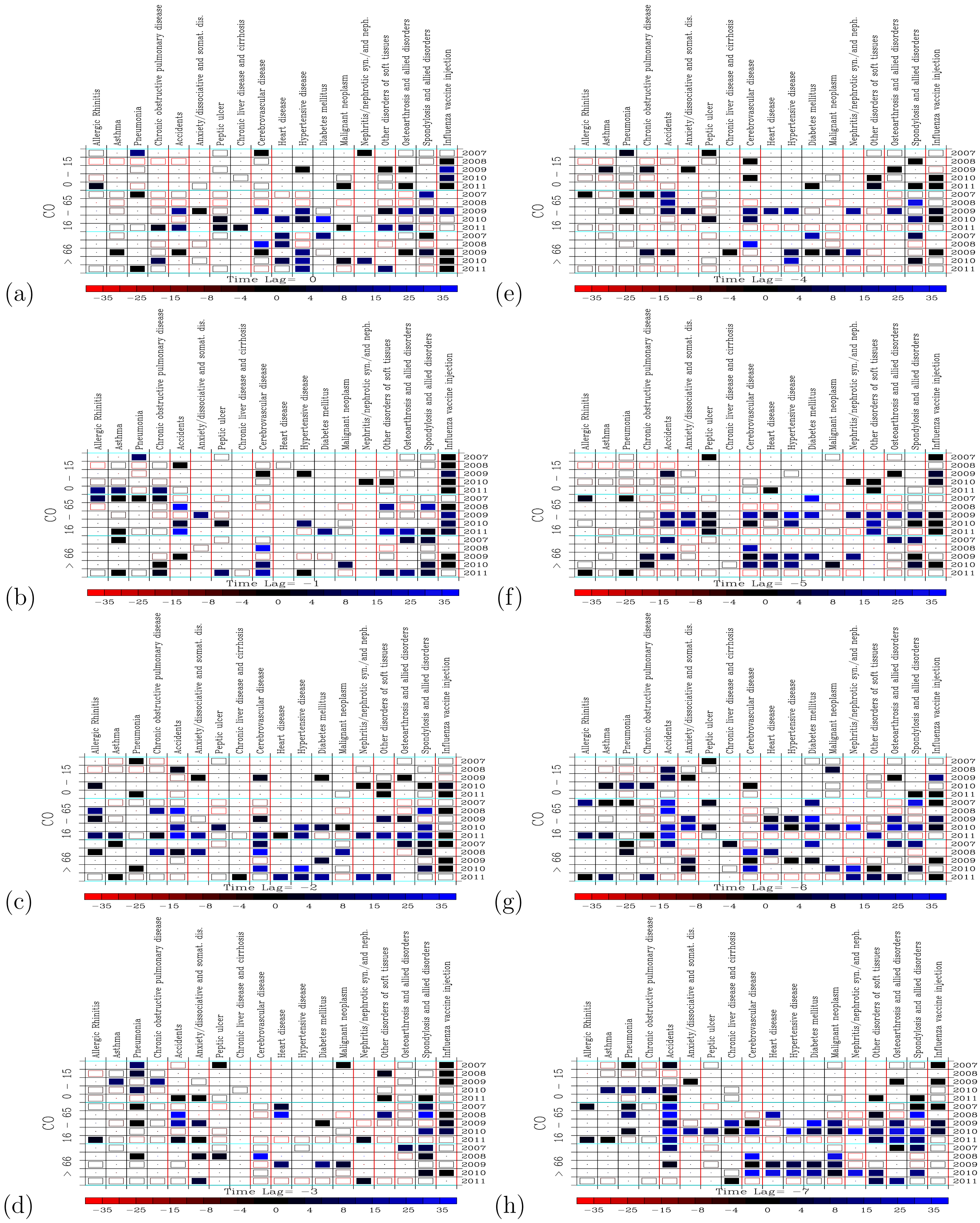

Figure 4. Distributrion of association coefficients $\beta_{\mathrm{i,j}}$ calculated for CO and 3 ages group of outpatients with respect to the 12 variables: (a) 0-, (b) 1-, (c) 2-, (d) 3-, (e) 4-, (f) 5-, (g) 6-, and (h) 7-day of time lags. Positive association coefficients are shown as blue colored filled squares, while negative association coefficients are shown as red colored open squares.

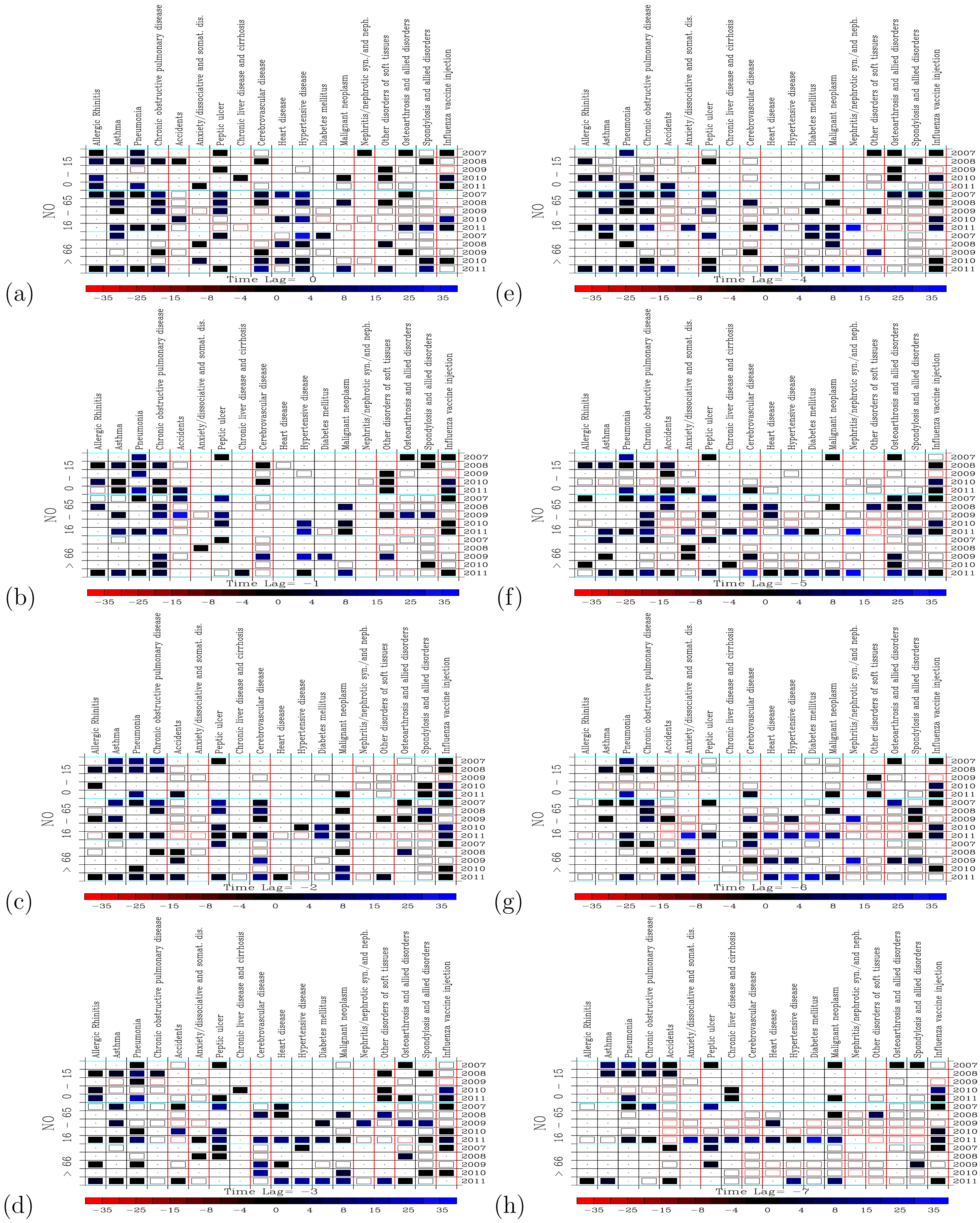

Figure 5. Distributrion of association coefficients $\beta_{\mathrm{i,j}}$ calculated for NO and 3 ages group of outpatients with respect to the 12 variables: (a) 0-, (b) 1-, (c) 2-, (d) 3-, (e) 4-, (f) 5-, (g) 6-, and (h) 7-day of time lags. Positive association coefficients are shown as blue colored filled squares, while negative association coefficients are shown as red colored open squares.

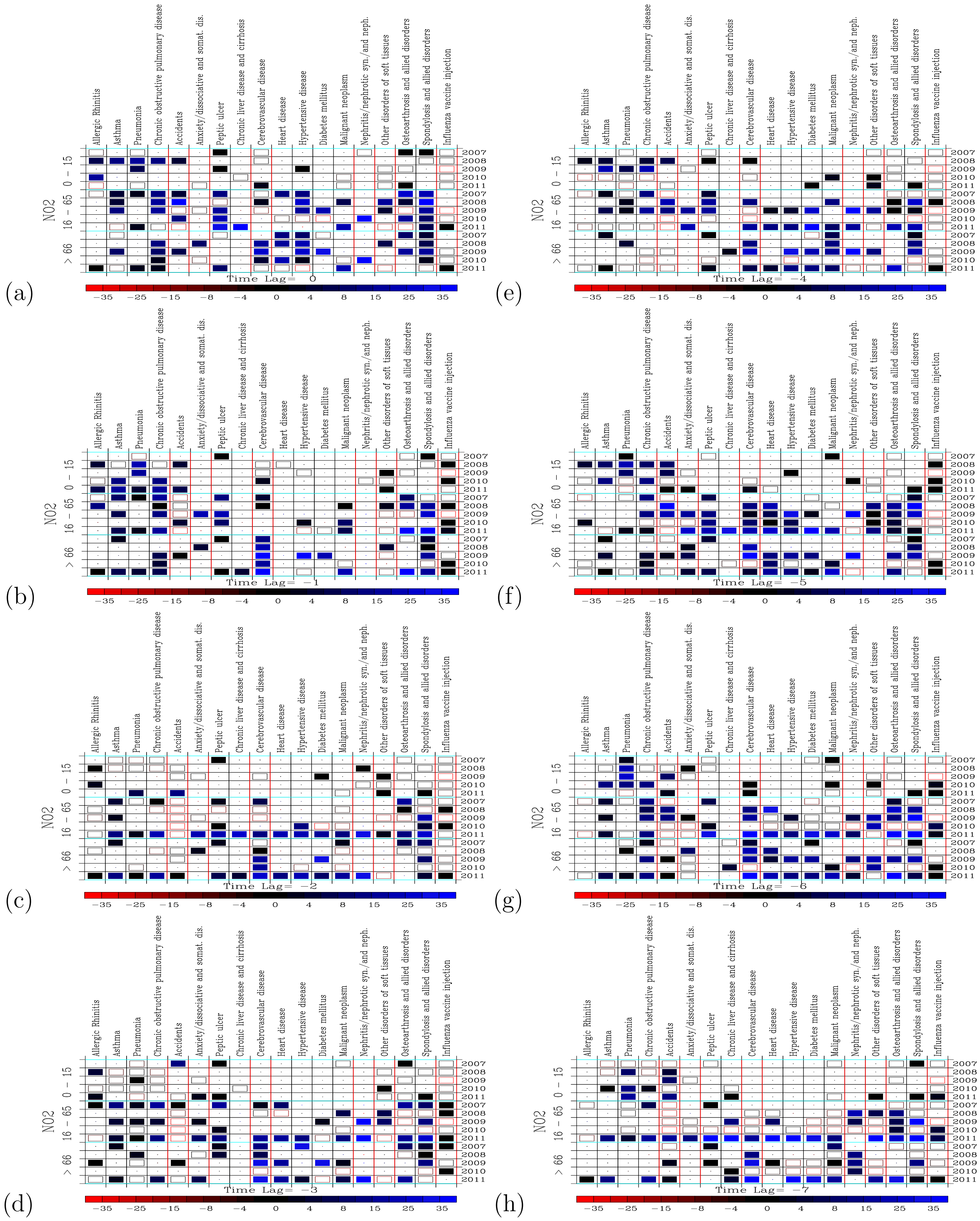

Figure 6. Distributrion of association coefficients $\beta_{i,j}$ calculated for NO2 and 3 ages group of outpatients with respect to the 12 variables: (a) 0-, (b) 1-, (c) 2-, (d) 3-, (e) 4-, (f) 5-, (g) 6-, and (h) 7-day of time lags. Positive association coefficients are shown as blue colored filled squares, while negative association coefficients are shown as red colored open squares.

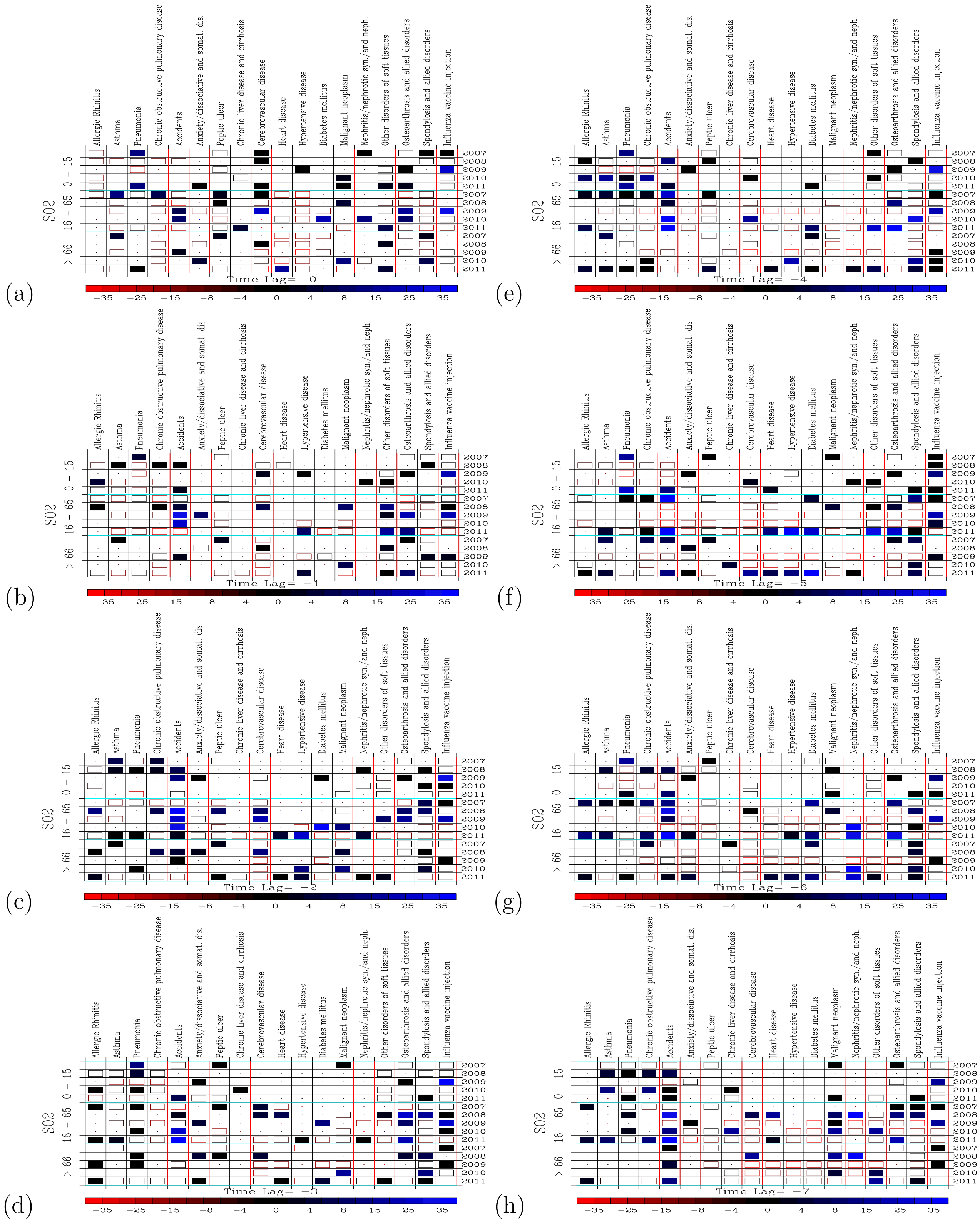

Figure 7. Distributrion of association coefficients $\beta_{\mathrm{i,j}}$ calculated for SO2 and 3 ages group of outpatients with respect to the 12 variables: (a) 0-, (b) 1-, (c) 2-, (d) 3-, (e) 4-, (f) 5-, (g) 6-, and (h) 7-day of time lags. Positive association coefficients are shown as blue colored filled squares, while negative association coefficients are shown as red colored open squares.

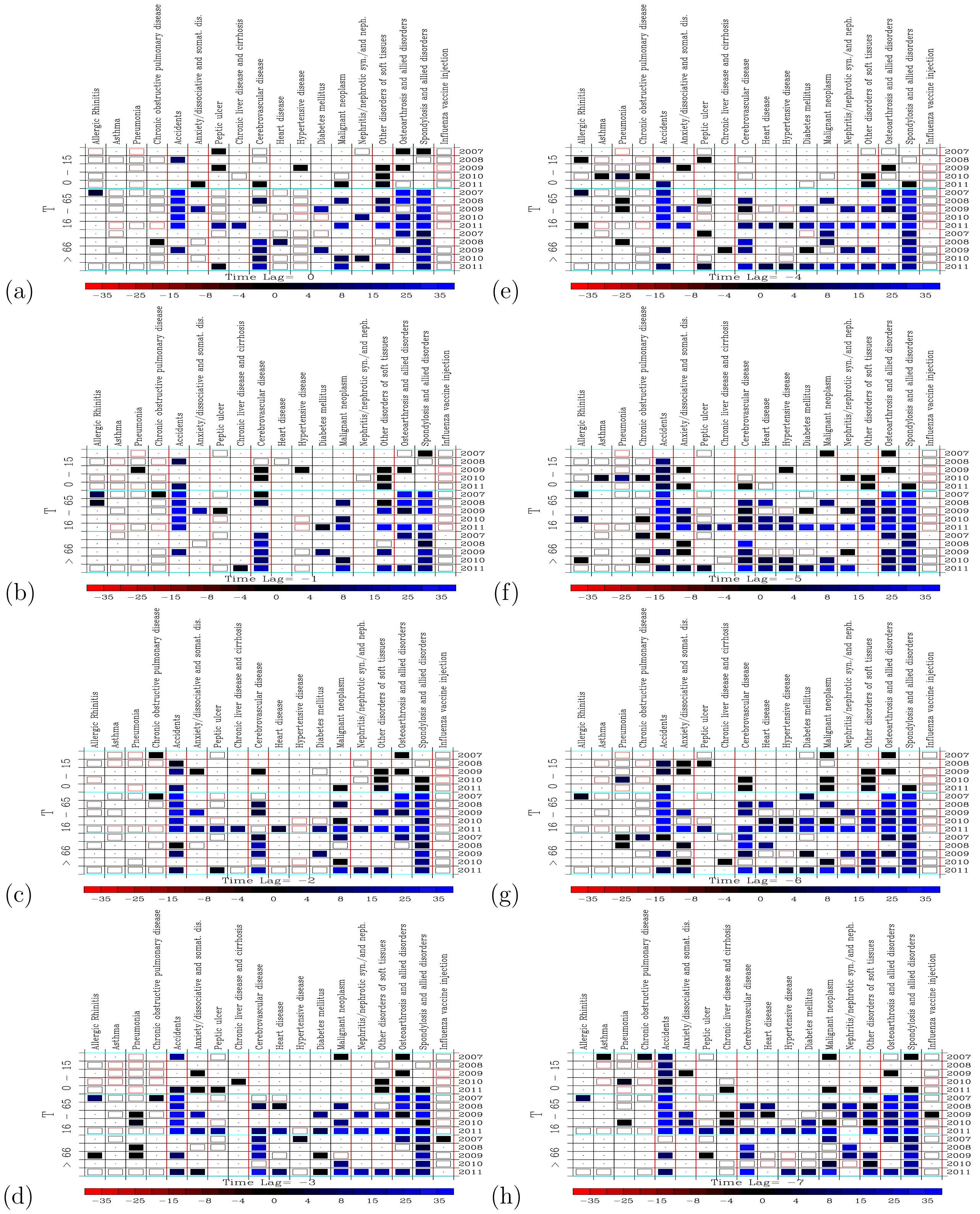

Figure 8. Distributrion of association coefficients $\beta_{i,j}$ calculated for T and 3 ages group of outpatients with respect to the 12 variables: (a) 0-, (b) 1-, (c) 2-, (d) 3-, (e) 4-, (f) 5-, (g) 6-, and (h) 7-day of time lags. Positive association coefficients are shown as blue colored filled squares, while negative association coefficients are shown as red colored open squares.

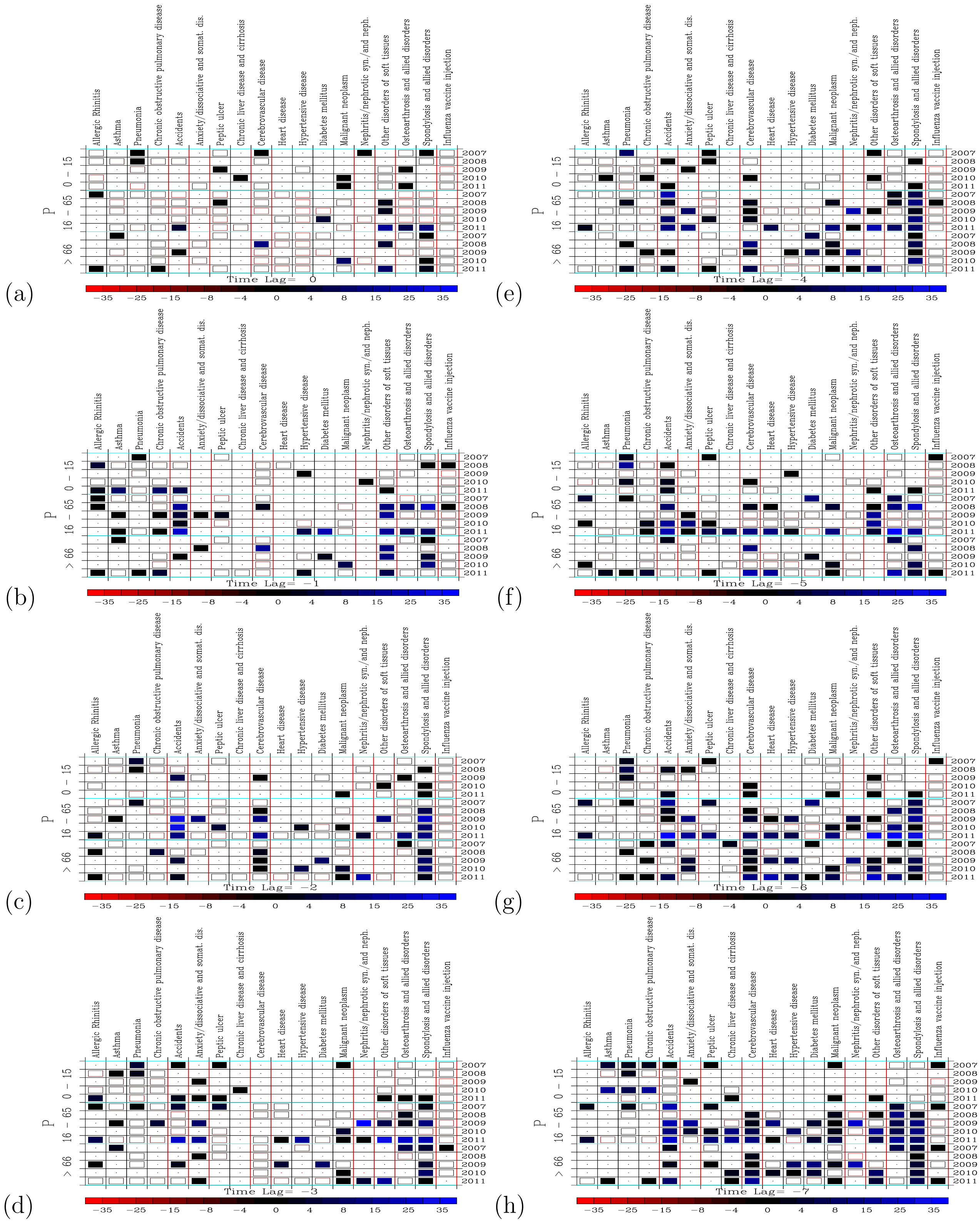

Figure 9. Distributrion of association coefficients $\beta_{i,j}$ calculated for P and 3 ages group of outpatients with respect to the 12 variables: (a) 0-, (b) 1-, (c) 2-, (d) 3-, (e) 4-, (f) 5-, (g) 6-, and (h) 7-day of time lags. Positive association coefficients are shown as blue colored filled squares, while negative association coefficients are shown as red colored open squares.

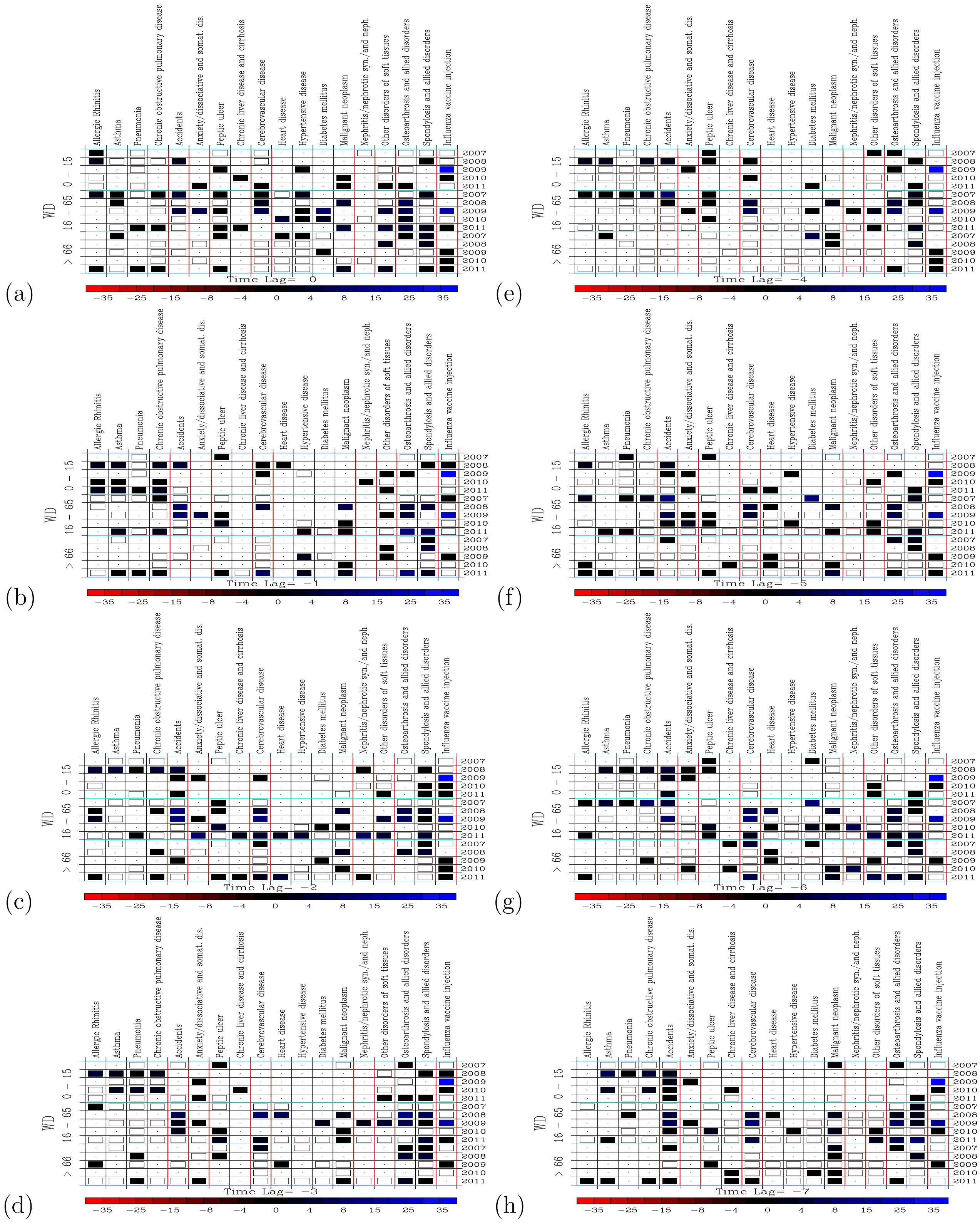

Figure 10. Distributrion of association coefficients $\beta_{\mathrm{i,j}}$ calculated for WD and 3 ages group of outpatients with respect to the 12 variables: (a) 0-, (b) 1-, (c) 2-, (d) 3-, (e) 4-, (f) 5-, (g) 6-, and (h) 7-day of time lags. Positive association coefficients are shown as blue colored filled squares, while negative association coefficients are shown as red colored open squares.

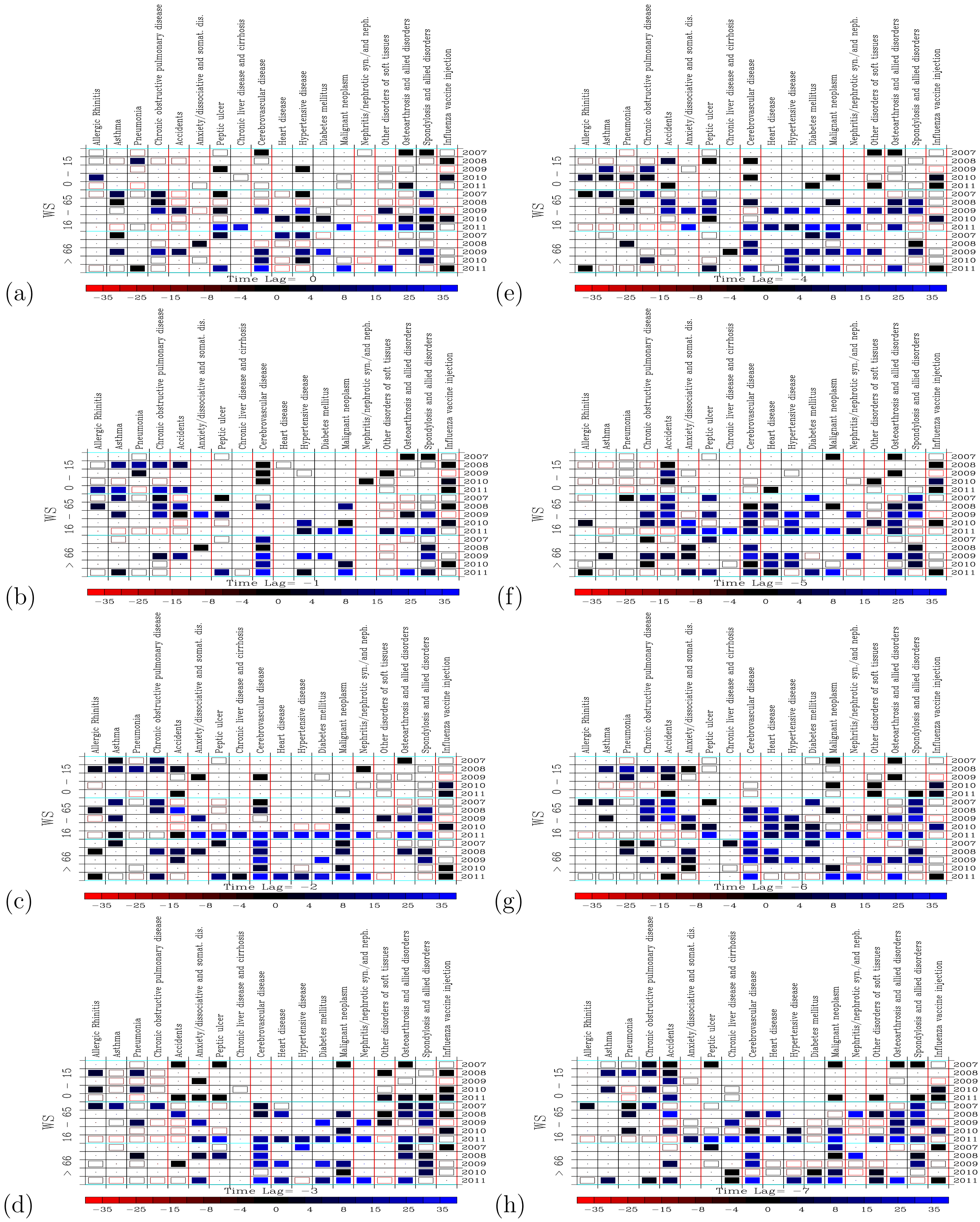

Figure 11. Distributrion of association coefficients $\beta_{i,j}$ calculated for WS and 3 ages group of outpatients with respect to the 12 variables: (a) 0-, (b) 1-, (c) 2-, (d) 3-, (e) 4-, (f) 5-, (g) 6-, and (h) 7-day of time lags. Positive association coefficients are shown as blue colored filled squares, while negative association coefficients are shown as red colored open squares.

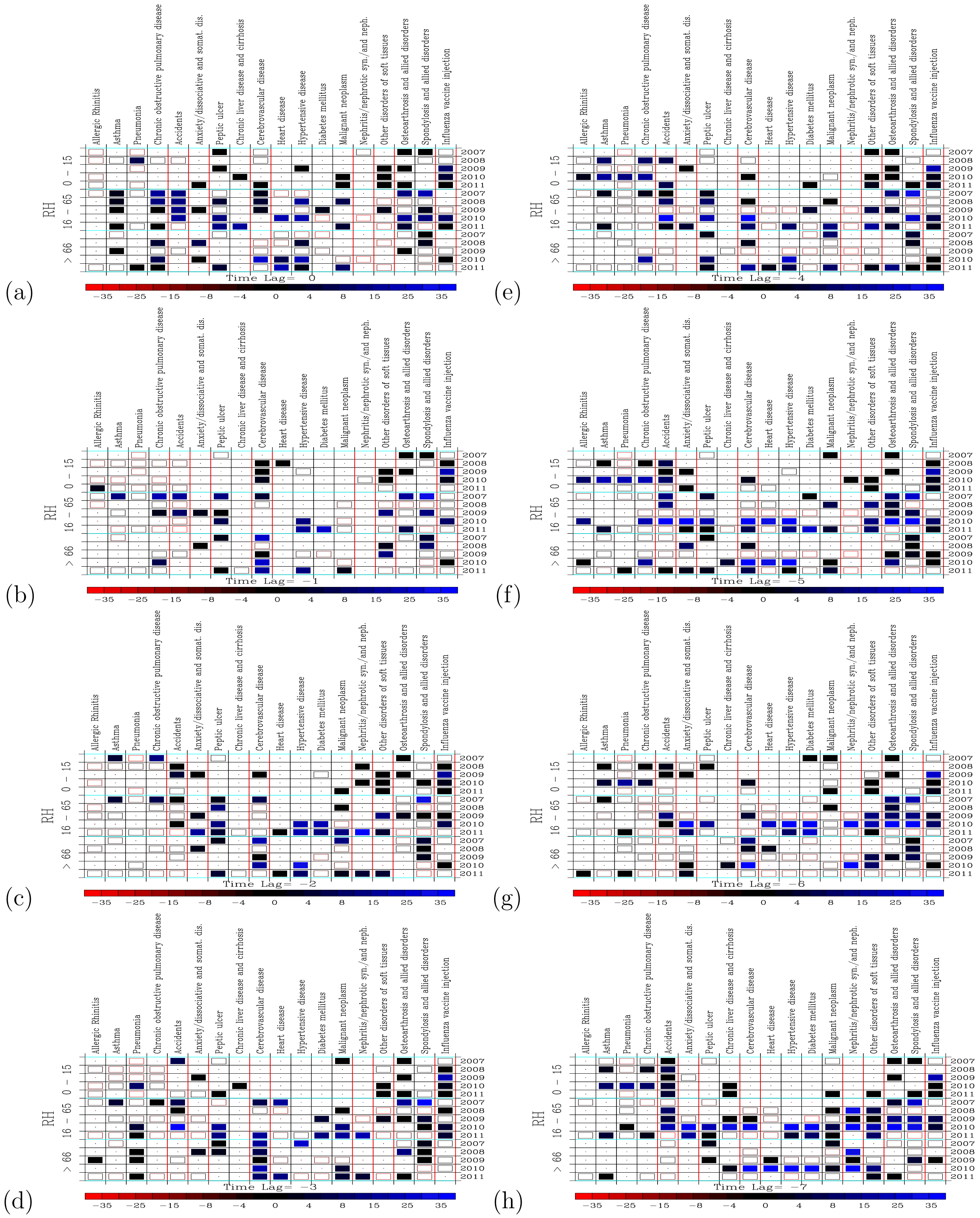

Figure 12. Distributrion of association coefficients $\beta_{i,j}$ calculated for RH and 3 ages group of outpatients with respect to the 12 variables: (a) 0-, (b) 1-, (c) 2-, (d) 3-, (e) 4-, (f) 5-, (g) 6-, and (h) 7-day of time lags. Positive association coefficients are shown as blue colored filled squares, while negative association coefficients are shown as red colored open squares.
